# Association between two-component systems gene mutation and *Mycobacterium tuberculosis* transmission revealed by whole genome sequencing

**DOI:** 10.1186/s12864-023-09788-2

**Published:** 2023-11-28

**Authors:** Yameng Li, Xianglong Kong, Yifan Li, Ningning Tao, Yawei Hou, Tingting Wang, Yingying Li, Qilin Han, Yao Liu, Huaichen Li

**Affiliations:** 1 Shandong University of Traditional Chinese Medicine, Jinan, Shandong 250014 People’s Republic of China; 2 Artificial Intelligence Institute, Qilu University of Technology (Shandong Academy of Sciences), Jinan, Shandong 250011 People’s Republic of China; 3 Department of Respiratory and Critical Care Medicine, The Third Affiliated Hospital of Shandong First Medical University (Affiliated Hospital of Shandong Academy of Medical Sciences), Jinan, Shandong 250031 People’s Republic of China; 4 Department of Respiratory and Critical Care Medicine, Shandong Provincial Hospital Affiliated to Shandong University, Shandong Provincial Hospital Affiliated to Shandong First Medical University, Jingwuweiqi Road, Huaiyin District, Jinan, Shandong 250021 People’s Republic of China; 5 Shandong First Medical University & Shandong Academy of Medical Sciences, Jinan, Shandong 250117 People’s Republic of China

**Keywords:** Two-component systems, *Mycobacterium tuberculosis*, Transmission, Whole genome sequencing

## Abstract

**Background:**

Two-component systems (TCSs) play a crucial role in the growth of *Mycobacterium tuberculosis* (*M. tuberculosis*). However, the precise regulatory mechanism of their contribution remain to be elucidated, and only a limited number of studies have investigated the impact of gene mutations within TCSs on the transmission of *M. tuberculosis*. Therefore, this study aims to explore the relationship between TCSs gene mutation and the global transmission of *M. tuberculosis*.

**Results:**

A total of 13531 *M.tuberculosis* strains were enrolled in the study. Most of the *M.tuberculosis* strains belonged to lineage4 (n=6497,48.0%), followed by lineage2 (n=5136,38.0%). Our results showed that a total of 36 single nucleotide polymorphisms (SNPs) were positively correlated with clustering of lineage2, such as *Rv0758 (phoR*, C820G), *Rv1747*(T1102C), and *Rv1057*(C1168T). A total of 30 SNPs showed positive correlation with clustering of lineage4, such as *phoR*(C182A, C1184G, C662T, T758G), *Rv3764c (tcrY*, G1151T), and *Rv1747* C20T. A total of 19 SNPs were positively correlated with cross-country transmission of lineage2, such as *phoR* A575C, *Rv1028c (kdpD*, G383T, G1246C), and Rv1057 G817T. A total of 41 SNPs were positively correlated with cross-country transmission of lineage4, such as *phoR*(T758G, T327G, C284G), *kdpD*(G1755A, G625C), *Rv1057* C980T, and *Rv1747* T373G.

**Conclusions:**

Our study identified that SNPs in genes of two-component systems were related to the transmission of *M. tuberculosis*. This finding adds another layer of complexity to *M. tuberculosis* virulence and provides insight into future research that will help to elucidate a novel mechanism of *M. tuberculosis* pathogenicity.

**Supplementary Information:**

The online version contains supplementary material available at 10.1186/s12864-023-09788-2.

## Background

Tuberculosis is a serious global health problem caused by *Mycobacterium tuberculosis* (*M. tuberculosis*), a pathogen that lives and thrives inside human cells [1]. It is a highly contagious and often fatal disease that affects millions of people worldwide, making it a significant burden on public health systems and societies. However, despite its enormous global burden, the factors that contribute to tuberculosis transmission are still poorly understood. Therefore, developing a better understanding of *M. tuberculosis* transmission is critical for guiding effective tuberculosis control strategies and reducing the disease’s burden on society.

Bacterial two-component systems (TCSs) are the most important sensing mechanisms that respond to a diverse range of ligands, including ions, gases, and metabolites. In pathogenic bacteria, TCSs play a crucial role in promoting pathogenesis by regulating bacterial gene expression in response to hostile host environments or metabolic stresses [2, 3]. The traditional two-component sensing system comprises a sensor kinase located in the cell membrane, which detects an extracellular ligand and subsequently activates through autophosphorylation on a cytoplasmic histidine residue. The *M. tuberculosis H37Rv* genome contains 190 transcriptional regulators, including 12 pairs of TCSs and 4 orphan proteins that belong to the two-component system family. These regulators play a role in regulating various aspects of *M. tuberculosis*, such as virulence, dormancy, persistence, and drug resistance. Some studies have suggested that TCSs may regulate the spread of *M. tuberculosis* through various pathways [4, 5]. For example, they can influence the growth, metabolism and environmental adaptation of the bacterium by regulating cell wall synthesis and degradation, maintaining intracellular redox balance, and modulating metabolic pathways [6, 7]. However, further research is needed to determine the specific regulatory mechanisms of TCSs in the process of *M. tuberculosis* transmission.

Whole genome sequencing (WGS) technology has significant implications for the study and treatment of *M. tuberculosis* [8]. This technique provides comprehensive information on the *M. tuberculosis* genome, including gene structure, function, regulation, and mutations. Such information can provide critical insights into the biological characteristics of the bacterium, its transmission patterns, drug resistance mechanisms, and new therapeutic targets. Additionally, WGS can help us understand *M. tuberculosis* evolution by identifying genetic differences and correlations between different strains, studying human-host co-adaptation and coevolution, and discovering new drugs and treatments for tuberculosis [9, 10]. In our research, WGS was used to study the influence of gene mutations in two-component systems on the worldwide transmission of *M. tuberculosis*. Specifically, the genome cluster was used to represent the transmission of *M. tuberculosis*.

## Results

### Characteristics of study samples

A total of 13,531 strains were used in this study including 5136(38.0%) strains belonged to lineage2 and 6497(48.0%) belonged to lineage 4. Lineage 2.2.1 was the dominant sub-lineage, accounting for 41.9%, followed by lineage 4.3 (16.6%), lineage 4.1 (13.9%) and lineage 4.8 (9.4%). The highest clustering rate observed within the lineage was lineage 4 at 0.704, while within sub-lineages, lineage 4.3 exhibited the greatest number of clustered strains. Among the clustered strains, lineage 4 had the most strains of cross-country and cross-regional distribution, while within sub-lineages, lineage 2.2.1 had the highest number of such strains (Table 1).

**Table 1:** Fundamental information of *Mycobacterium tuberculosis*

Characteristic		Number (%)	
Lineage	Lineage1	851(6.3)	
	Lineage2	5136(38.0)	
	Lineage3	970(7.2)	
	Lineage4	6497(48.0)	
	Lineage5	38(0.3)	
	Lineage6	10(0.1)	
	Lineage7	29(0.2)	
Sub-lineage	Lineage2.1	46(0.4)	
	Lineage2.2.1	4832(41.7)	
	Lineage2.2.2	258 (2.2)	
	Lineage4.1	1614(13.9)	
	Lineage4.2	427(3.7)	
	Lineage4.3	1919(16.6)	
	Lineage4.4	626(5.4)	
	Lineage4.8	1086(9.4)	
	Other sub-lineage4	781(6.7)	
Clustered strains	Lineage1	Clustered strains	319(37.5)
		No-clustered strains	532(62.5)
	Lineage2	Clustered strains	2999(58.4)
		No-clustered strains	2137(21.6)
	Lineage3	Clustered strains	468(48.2)
		No-clustered strains	502(51.8)
	Lineage4	Clustered strains	4574(70.4)
		No-clustered strains	1923(29.6)
Clustered strains _size	Lineage2	Large clustered strains	663(22.1)
		Medium clustered strains	1264(42.1)
		Small clustered strains	1072(35.7)
	Lineage4	Large clustered strains	1361(29.8)
		Medium clustered strains	2017(44.1)
		Small clustered strains	1196(26.1)
Cross country	Lineage2	Cross country	330(11.0)
		Within country	2669(89.0)
	Lineage4	Cross country	374(8.2)
		Within country	4200(91.8)
Cross regional	Lineage2	Cross regional	321(10.7)
		Within regional	2678(89.3)
	Lineage4	Cross regional	338(7.4)
		Within regional	4236(92.6)

### Relationship between TCSs gene mutation and lineage transmission

**Fig. 1:**
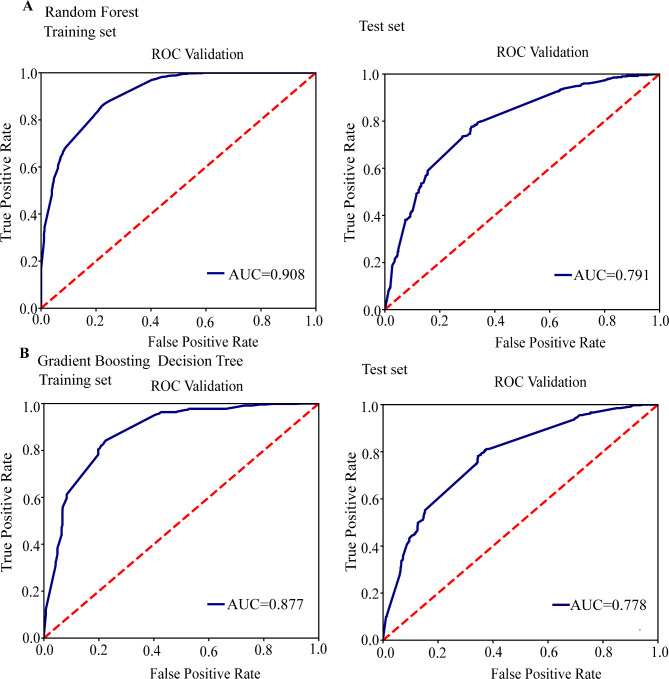
ROC curve analysis was conducted to evaluate the performance of models for cluster analysis within lineage 2. (**A**) ROC analysis showing the performance of the random forest model. (**B**) ROC analysis showing the performance of the gradient boosting decision tree

We studied the relationship of gene mutation in the two-component system and lineage transmission. The random forest and gradient boosting decision tree models of lineage 1 were successfully established. For further details see Additional file 2: Table S9 and Additional file 1: Fig. S6. Subsequently, a generalized linear mixed model was established to analyze 60 variables that represented the intersection of random forest and gradient boosting decision tree (Additional file 2: Tables S4 and S5). A total of 31 SNPs showed a positive correlation with clustering of lineage1(*P* < 0.05), including 15 synonymous SNPs and 16 nonsynonymous SNPs, such as *Rv3764c* (*tcrY*, T1354C, OR,1.975; 95%CI,1.456–2.680), *Rv1747*(C980T, OR, 2.344; 95%CI,1.723–3.19), *Rv1057*(C177T, OR,1.539, 95%CI, 1.24–1.91), and *Rv3245c* (*mtrB*, C831T, G300T) (Additional file 2: Table S24). The results showed that 31 SNPs increased the risk of lineage1 transmission. For lineage2, the random forest and gradient boosting decision tree models were successfully established (Table 2; Fig. 1). Subsequently, a generalized linear mixed model was established to examine 60 variables that contributed to the gradient boosting decision tree and random forest models (Additional file 2: Tables S4 and S5). A total of 36 SNPs showed a positive correlation with clustering of lineage2(*P* < 0.05), including 12 synonymous SNPs and 24 nonsynonymous SNPs, such as *Rv0758* (*phoR*, C820G), *Rv1747* T1102C, *Rv1057* C1168T, *Rv3764c* (*tcrY*, C284A), *Rv0982*(*mprB*, G910A), *Rv2247*(accD6, G567A, T600C), *Rv1027c* (*KdpE*, G178A, C626A), *Rv3245c* (*mtrB*, A971G, G1110A), and Rv3765c (tcrX, G293C) (Additional file 2: Table S25). The results showed that 36 SNPs increased the risk of lineage2 transmission. For lineage3, the random forest and gradient boosting decision tree models of lineage 3 were successfully established (Additional file 2: Table S10 and Additional file 1: Fig. S7). Subsequently, a generalized linear mixed model was established to analyze 60 variables that represented the common features from both gradient boosting decision tree and random forest models (Additional file 2: Tables S4 and S5). A total of 29 SNPs showed a positive correlation with clustering of lineage3(*P* < 0.05), including 11 synonymous SNPs and 18 nonsynonymous SNPs, such as *Rv0758* (*phoR*, G448T, G694T), *Rv3764c* (*tcrY*, C278T), *Rv1747*(G2188A, C460T), *Rv3765c* (tcrX, G415A), *Rv1057* G186A, *Rv0982*(*mprB*, G1477A), *Rv1032c* (*trcS*, T946C), *Rv1027c* (*KdpE*, C45T), and *Rv3245c* (*mtrB*, C24T) (Additional file 2: Table S23). The results showed that 29 SNPs increased the risk of transmission of lineage3. For lineage4, the random forest and gradient boosting decision tree models of lineage 4 were successfully established (Additional file 2: Table S11 and Additional file 1: Fig. S8). Subsequently, a generalized linear mixed model was established to analyze a total of 60 variables that represented the intersection of random forest and gradient boosting decision tree (Additional file 2: Tables S4 and S5). A total of 30 SNPs showed a positive correlation with clustering of lineage4(*P* < 0.05), including 15 synonymous SNPs and 15 nonsynonymous SNPs, such as *Rv0758(phoR*, C182A, C1184G, C662T, T758G), *Rv3764c* (*tcrY*, G1151T), *Rv1747* C20T, *Rv3765c* (*tcrX*, C45G), *Rv1057* C585A, *Rv1032c* (*trcS*, G977T), *Rv2247*(*accD6*, G957A), and *Rv3245c* (*mtrB*, T33C) (Additional file 2: Table S7). The results showed that 30 SNPs increased the risk of transmission of lineage4.

**Table 2:** The performance of various models for discriminating clustered strains from non-clustered strains in the lineage2 cohort

Parameters	Training set (n = 3595, 2081 clustered strains, 1514 non-clustered strains)		Test set (n = 1541, 918 clustered strains, 623 non-clustered strains)	
	**Random Forest**	**Gradient Boosted Classification Tree**	**Random Forest**	**Gradient Boosted Classification Tree**
Kappa	0.641	0.613	0.454	0.442
AUC (95% CI)	0.908 (0.899, 0.917)	0.877 (0.866, 0.888)	0.791 (0.771, 0.811)	0.778 (0.757, 0.799)
Sensitivity (95% CI)	0.873 (0.862, 0.884)	0.836 (0.824, 0.848)	0.786 (0.766, 0.806)	0.807 (0.787, 0.827)
Specificity (95% CI)	0.762 (0.748, 0.776)	0.779 (0.765, 0.793)	0.666 (0.642, 0.690)	0.628 (0.604, 0.652)
PPV (95% CI)	0.837 (0.825, 0.849)	0.845 (0.833, 0.857)	0.771 (0.750, 0.792)	0.741 (0.719, 0.763)
NPV (95% CI)	0.811 (0.798, 0.824)	0.767 (0.753, 0.781)	0.686 (0.663, 0.709)	0.712 (0.689, 0.735)
PLR (95% CI)	4.437 (4.415, 4.459)	3.625 (3.597, 3.653)	2.451 (2.402, 2.50)	2.571 (2.528, 2.614)
NIR (95% CI)	0.225 (0.15, 0.30)	0.276 (0.198, 0.354)	0.408 (0.313, 0.503)	0.389 (0.301, 0.477)
Accuracy (95% CI)	0.827 (0.815, 0.839)	0.813 (0.8, 0.826)	0.737 (0.715, 0.759)	0.730 (0.708, 0.752)

AUC, area under the curve; PPV, positive predictive value; NPV, negative predictive value; PLR, positive likelihood ratio; NLR, negative likelihood ratio; CI, confidence

### Relationship between TCSs gene mutation and sub-lineage transmission

The random forest and gradient boosting decision tree models of lineage 2.2.1, lineage 2.2.2, lineage 4.1, lineage 4.2, lineage 4.4 and lineage 4.8 were successfully established (Additional file 2: Tables S4, S5, S12–S18) (Additional file 1: Figs. S9–S15). The results of the generalized linear mixed model showed that 30 SNPs were positively correlated with clustering of lineage2.2.1(*P* < 0.05), including 10 synonymous SNPs and 20 nonsynonymous SNPs, such as *Rv2027c* (*dosT*, T874C), *Rv1028c* (*kdpD*, G2453A), *Rv1057* C29G, Rv0982 (mprB, G1477A), Rv1032c (trcS, T946C), Rv1027c (KdpE, G178A, C626A), Rv3245c (mtrB, A971G), and *Rv2247*(*accD6*, G567A) (Additional file 2: Table S28). The results showed that 30 SNPs increased the risk of transmission of lineage2.2.1. For lineage2.2.2, a total of 16 SNPs showed a positive correlation with clustering (*P* < 0.05), including 8 synonymous SNPs and 8 nonsynonymous SNPs, such as *Rv2027c* (*dosT*, C215T), *Rv1028c* (*kdpD*, G2085), *Rv0982* (*mprB*, G910A), and *Rv3245c* (*mtrB*, T33C) (Table 3). The results showed that 16 SNPs increased the risk of transmission of lineage2.2.2.

**Table 3:** Generalized linear mixed model analysis on clustered and non-clustered strains in the lineage2.2.2 cohort

Gene	Position	SNP	Amino acid changes		*P* value	OR(95%CI)
Rv0982	1,098,417	G910A	Asp304Asn	< 0.001		2.092(1.958,2.234)
Rv1743	1,969,405	G402A	Gly134Gly	< 0.001		2.063(1.808,2.354)
Rv2027c	2,274,294	C215T	Thr72Ile	< 0.001		2.442(1.952,3.056)
Rv1747	1,973,911	C282T	Pro94Pro	< 0.001		2.442(2.081,2.866)
Rv1626	1,828,320	C141T	Gly47Gly	< 0.001		1.853(1.637,2.098)
Rv0014c	16,352	C1119T	Ala373Ala	< 0.001		2.098(1.815,2.425)
Rv2247	2,520,964	G222A	Thr74Thr	0.009		0.436(0.317,0.6)
Rv0758	852,994	G599T	Gly200Val	0.005		0.414(0.302,0.566)
Rv0014c	16,465	G1006C	Val336Leu	< 0.001		2.462(1.968,3.08)
Rv0845	942,450	C1261T	Leu421Leu	< 0.001		2.524(2.014,3.165)
Rv1266c	1,414,305	C1536G	Asp512Glu	< 0.001		2.535(2.022,3.177)
Rv0490	580,362	C1014G	Ala338Ala	0.004		0.402(0.293,0.55)
Rv3132c	3,498,065	C1201A	Arg401Arg	< 0.001		2.43(1.943,3.04)
Rv1028c	1,151,173	G514C	Asp172His	0.001		0.492(0.399,0.608)
Rv1028c	1,149,602	G2085A	Ser695Ser	< 0.001		2.838(2.199,3.662)
Rv3765c	4,211,244	A541C	Ser181Arg	0.051		0.526(0.378,0.731)
Rv0981	1,096,890	T69C	Asn23Asn	0.006		0.51(0.399,0.651)
Rv1675c	1,900,608	G368A	Gly123Glu	< 0.001		2.442(1.952,3.056)
Rv1743	1,969,938	C935T	Pro312Leu	0.008		1.505(1.289,1.758)
Rv1743	1,970,586	C1583T	Ala528Val	0.024		0.469(0.335,0.656)
Rv0845	941,473	T284A	Phe95Tyr	< 0.001		2.032(1.684,2.452)
Rv0602c	699,629	T171C	His57His	0.001		1.855(1.542,2.232)
Rv2984	3,339,998	C145G	Pro49Ala	0.031		0.487(0.349,0.679)
Rv0845	942,410	T1221C	Val407Val	0.214		1.357(1.062,1.733)
Rv1027c	1,148,482	C626A	Ser209*	0.095		0.838(0.754,0.931)
Rv1027c	1,148,459	G649T	Glu217*	0.188		1.166(1.038,1.311)
Rv0601c	698,968	C27T	Gly9Gly	< 0.001		0.532(0.452,0.626)
Rv0758	853,066	G671T	Gly224Val	0.377		1.115(0.986,1.261)
Rv0014c	16,119	G1352T	Arg451Leu	0.042		1.379(1.177,1.614)
Rv1813c	2,055,937	G176A	Gly59Glu	0.701		1.063(0.908,1.245)
Rv1747	1,974,919	C1290T	Arg430Arg	0.556		0.898(0.747,1.079)
Rv1368	1,541,426	G407T	Arg136Leu	0.538		0.893(0.743,1.074)
Rv0981	1,097,238	G417A	Pro139Pro	0.254		0.86(0.754,0.981)

OR, odds ratio; CI, confidence interval

For lineage4.1, a total of 22 SNPs showed a positive correlation with clustering (*P* < 0.05), including 12 synonymous SNPs and 10 nonsynonymous SNPs, such as *Rv1028c* (*kdpD*, G943A, G2136A), *Rv2027c* (*dosT*, G1256A), *Rv1032c (trcS*, G857A), and *Rv1747* C20T (Additional file 2: Table S29). Our results showed that these 22 SNPs increased the risk of transmission of lineage4.1. For lineage4.2, the result of the generalized linear mixed model showed that 7 SNPs were positively correlated with clustering (*P* < 0.05), including 2 synonymous SNPs and 5 nonsynonymous SNPs, such as *Rv0758* (*phoR*, C182A), *Rv0930*(*pstA1*, G895T, C913T), *Rv3245c* (*mtrB*, C1113A), and *Rv2247*(*accD6*, G957A, T600C) (Additional file 2: Table S30). The results showed that 7 SNPs increased the risk of transmission of lineage4.2. For lineage4.4, the result of the generalized linear mixed model showed that 18 SNPs were positively correlated with clustering (*P* < 0.05), including 9 synonymous SNPs and 9 nonsynonymous SNPs, such as *Rv0982*(*mprB*, G901A, G230C), *Rv1028c* (*kdpD*, C1102T), *Rv0758* (*phoR*, C662T, T758G, A341C), *Rv0982* (*mprB*, G901A. G230C), and *Rv3245c* (*mtrB*, C1083T), (Additional file 2: Table S31). The results showed that 18 SNPs increased the risk of transmission of lineage4.4. For lineage4.8, the result of the generalized linear mixed model showed that 15 SNPs were positively correlated with clustering (*P* < 0.05), including 5 synonymous SNPs and 10 nonsynonymous SNPs, such as *Rv1028c* (*kdpD*, C643T), *Rv3764c* (*tcrY*, G1151T), *Rv1032c* (*trcS*, C1375G), *Rv0758(phoR*, T148G), and *Rv2247*(*accD6*, T600C), (Additional file 2: Table S32). The results showed that 15 SNPs increased the risk of transmission of lineage4.8.

### Relationship between TCSs gene mutation and cluster size

For analyzing the relationship of gene mutation in the two-component system and cluster size, the random forest and gradient boosting decision tree models for lineage2 and lineage4 were successfully established.

The random forest and gradient boosting decision tree models of lineage 2 and lineage4 were successfully established. (Additional file 2: Tables S6, S19, and S20). The results of the generalized linear mixed model indicated that 25 SNPs were positively correlated with cluster size of lineage2(*P* < 0.05), including 14 synonymous SNPs and 11 nonsynonymous SNPs, such as *Rv0758*(*phoR*, C820G), *Rv1747* C696A, *Rv1028c* (*kdpD*, G383T), *Rv1057* C653T, *Rv1032c* (*trcS*, C1202T), *Rv2247*(*accD6*, G567A), *Rv3245c* (*mtrB*, A1660G), *Rv0982*(*mprB*, G910A, C780T), and *Rv1027c* (*KdpE*, C626A) (Additional file 2: Table S33). The results showed that 25 SNPs increased the risk of small clusters, medium clusters, and large clusters of lineage2. The results of the generalized linear mixed model indicated that a total of 30 significant SNPs were positively correlated with cluster size of lineage4(*P* < 0.05), including 13 synonymous SNPs and 17 nonsynonymous SNPs, such as *Rv0758*(*phoR*, C182A, C1184G, C662T), *Rv1028c*(*kdpD*, C2320T, A1982T, G943A, A214G), *Rv3132c*(*devS*,C552G), *Rv0982*(*mprB*, G901A), *Rv3764c*(*tcrY*, G1151T), *Rv1747* C20T, *Rv0982*(*mprB*, G910A, C1191G), *Rv1032c* (*trcS*, G977T, C1445T), *Rv2247*(*accD6*, G957A, T600C), and *Rv3245c* (*mtrB*, A778G) (Additional file 2: Table S34). The results showed that 30 SNPs increased the risk of small clusters, medium clusters, and large clusters of lineage4.

### Relationship between TCSs gene mutation and cross-country transmission

Random forest and gradient boosting decision tree models were successfully implemented to analyze the cross-country transmission of *M. tuberculosis* via gene mutations in TCSs, specifically focusing on lineage2 and lineage4. (Additional file 2: Tables S7, S2, and S22; Additional file 1: Figs. S16 and S17)

The results of the generalized linear mixed model showed that a total of 19 SNPs were positively correlated with cross-country transmission of lineage2(*P* < 0.05), including 6 synonymous SNPs and 13 nonsynonymous SNPs, such as *Rv0758*(*phoR*, A575C), *Rv1028c* (*kdpD*, G383T, G1246C), *Rv1057* G817T, *Rv0982*(*mprB*, G910A), *Rv1747* T373G. *Rv0982*(*mprB*, G910A, C1317G), *Rv1027c* (*KdpE*, G178A) (Additional file 2: Table S35). The results showed that 19 SNPs increased the risk of cross-country transmission of lineage2. A total of 41 SNPs were positively correlated with cross-country transmission of lineage4 (*P* < 0.05), including 20 synonymous SNPs and 21 nonsynonymous SNPs, such as *Rv0758*(*phoR*, T758G, T327G, C284G), *Rv1028c*(*kdpD*, G1755A, G625C), *Rv1057* (C980T, *Rv1747* T373G), *Rv3764c* T736C, *Rv0982*(*mprB*, G1323A), *Rv2247*(*accD6*, G181A, C700T, G36A), *Rv1027c* (*KdpE*, G381A), *Rv1032c* (*trcS*, T188G, G977T, G571A),and *Rv3245c*(*mtrB*, T354C, G1011A) (Additional file 2: Table S36). The results showed that 41 SNPs increased the risk of cross-country transmission of lineage4.

### Relationship between TCSs gene mutation and cross-regional transmission

The random forest and gradient boosting decision tree models were successfully established for analyzing the cross-regional transmission of *M. tuberculosis* through gene mutations in TCSs within lineage 2 and lineage 4. (Table 4, Additional file 2: Tables S8 and S23) (Additional file 1: Figs. S18 and S19).

**Table 4:** The performance of various models for discriminating cross-regional from within-regional in the lineage4 cohort

Parameters	Training set (n = 3201, 245 cross-regional strains,2956 within-regional trains)		Test set (n = 1373, 93 cross-regional strains, 1280 within-regional strains)	
	**Random Forest**	**Gradient Boosted Classification Tree**	**Random Forest**	**Gradient Boosted Classification Tree**
Kappa	0.649	0.553	0.472	0.435
AUC (95% CI)	0.954 (0.947, 0.961)	0.941 (0.933, 0.949)	0.927 (0.913, 0.941)	0.922 (0.908, 0.936)
Sensitivity (95% CI)	0.981 (0.976, 0.986)	0.458 (0.441, 0.475)	0.971 (0.962, 0.980)	0.363 (0.338, 0.388)
Specificity (95% CI)	0.981 (0.976, 0.986)	0.990 (0.987, 0.993)	0.971 (0.962, 0.980)	0.984 (0.977, 0.991)
PPV (95% CI)	0.732 (0.717, 0.747)	0.783 (0.769, 0.797)	0.543 (0.517, 0.569)	0.649 (0.624, 0.674)
NPV (95% CI)	0.969 (0.963, 0.975)	0.958 (0.951, 0.965)	0.962 (0.952, 0.972)	0.951 (0.940, 0.962)
PLR (95% CI)	23.808 (23.802, 23.814)	18.728 (18.721, 18.735)	14.323 (14.315, 14.331)	13.142 (13.131, 13.153)
NIR (95% CI)	0.042 (-0.017, 0.101)	0.053 (-0.014, 0.120)	0.070 (0.006, 0.134)	0.076 (0, 0.152)
Accuracy (95% CI)	0.954 (0.947, 0.961)	0.951 (0.944, 0.958)	0.937 (0.924, 0.950)	0.938 (0.925, 0.951)

AUC, area under the curve; PPV, positive predictive value; NPV, negative predictive value; PLR, positive likelihood ratio; NLR, negative likelihood ratio; CI, confidence

The results of the generalized linear mixed model showed that a total of 22 SNPs were positively correlated with cross-regional transmission of lineage2(*P* < 0.05), including 9 synonymous SNPs and 13 nonsynonymous SNPs, such as *Rv0758*(*PhoR*, C385A), *Rv1057*(G817T, A1136G), *Rv2027c* (*dosT*, C1343T), *Rv1028c* (*kdpD*, G383T), *Rv1747*(T373G). *Rv0982*(*mprB*, G910A, C1317G), *Rv1027c* (*KdpE*, G178A), and *Rv1032c* (*trcS*, A886G, G748A, G561A) (Additional file 2: Table S37). The results showed that 22 SNPs increased the risk of cross-regional transmission of lineage2. A total of 34 SNPs were positively correlated with cross-regional transmission of lineage4(*P* < 0.05), including 13 synonymous SNPs and 21 nonsynonymous SNPs, such as *Rv0758*(*phoR*, T758G, C805T, C294A, C1184G), *Rv1057* C980T, *Rv1028c* (*kdpD*, G1755A, G1266C, G625C), *Rv1747* C2112T, *Rv2247*(*accD6*, G36A), *Rv1027c* (*KdpE*, G381A), *Rv1032c* (*trcS*, T188G, G977T, G571A), and *Rv3245c* (*mtrB*, G390A), (Additional file 2: Table S38). The results showed that 34 SNPs increased the risk of cross-regional transmission of lineage4.

**Fig. 2:**
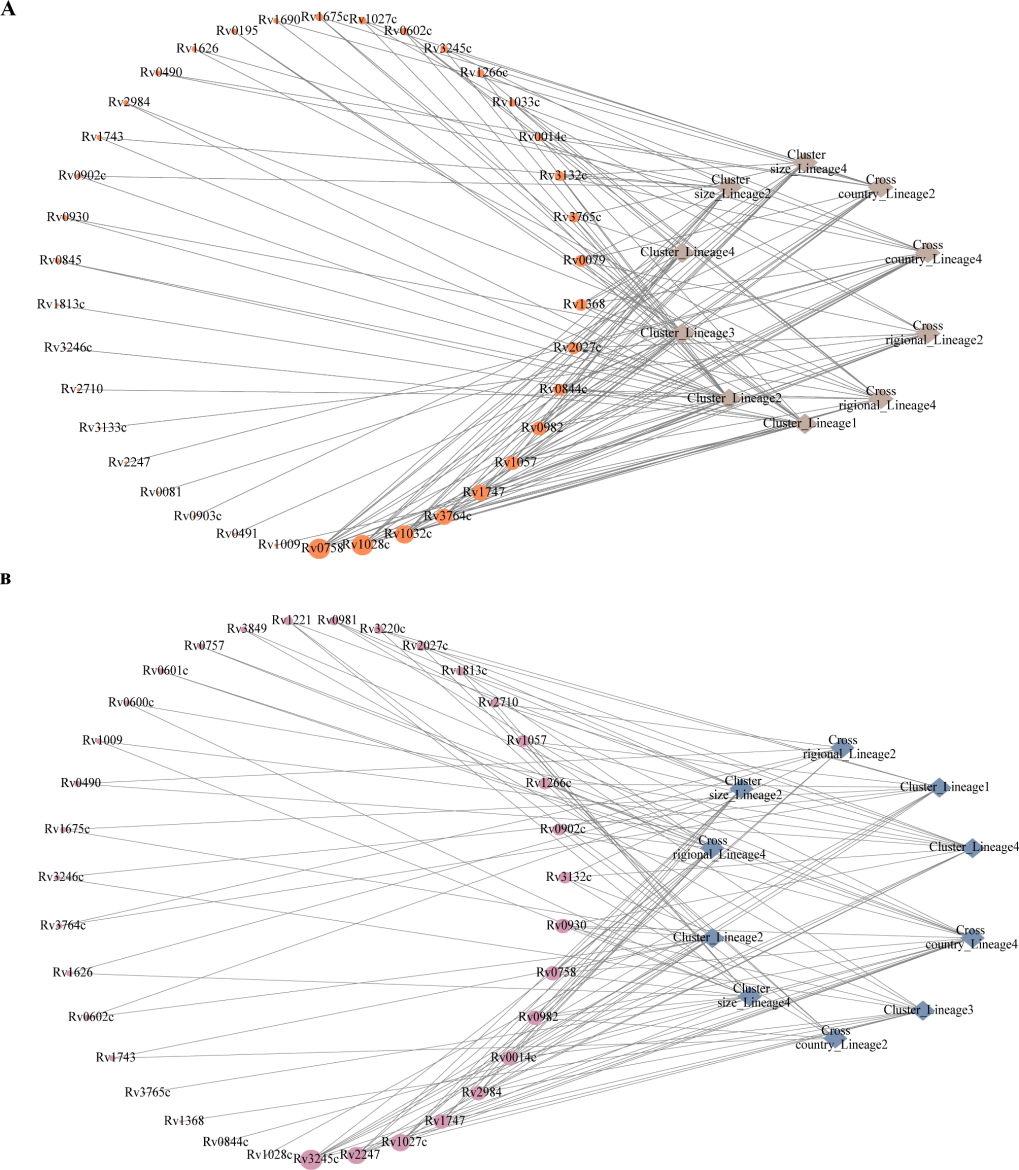
The effect of two-component system gene mutations on various lineages. (**A**) The effect of missense mutations in two-component system genes on various lineages. (**B**) The effect of synonymous mutations in two-component system genes on various lineages

The above findings revealed that synonymous SNPs and nonsynonymous SNPs in *PhoR, mprB* and *Rv1747* were significantly related to the transmission of various lineages of *M.tuberculosis*, including cross-country and cross-regional transmission. In addition, missense mutations in *KdpD* and *trcS*, as well as synonymous mutations in *Rv3245c* (*mtrB*), *Rv2247* (*accD6*) and *Rv1027c* (*KdpE*), were also significantly related to the transmission of various lineages of *M.tuberculosis*, including cross-country and cross-regional transmission (Fig. 2). These mutations increased the risk of transmission of *M.tuberculosis*.

## Discussion

The transmission factors of pathogenic bacteria have always been elusive, and the transmission factors of *M. tuberculosis* were also very complicated. Until now, there has been no research on whether the gene mutation in TCSs is related to the transmission of *M. tuberculosis*. Our research showed that there were SNPs in the genes of the two-component system, which increased the risk of the transmission of *M. tuberculosis*. With the continuous discovery of new two-component systems, these findings showed that the gene mutation of TCSs has universal and extensive significance for the transmission of *M. tuberculosis*.

Our study revealed that SNPs in *PhoR* increased the risk of transmission of *M. tuberculosis*, including C820G, G448T, G694T, C182A, C1184G, C662T, T758G, C820G, C182A, C1184G, and C662T. *PhoP*, part of the two-component system *PhoR*-*PhoP*, is the response regulator protein that activates or represses the genes of the regulon [11]. The *PhoR* gene encodes a kinase and is considered one of the main signaling pathways involved in regulating phosphate metabolism in *M. tuberculosis*. This gene senses changes in extracellular phosphate concentration, activating the *PhoP-PhoR* two-component system to regulate the adaptive response of *M. tuberculosis* [12, 13]. The increased risk of *M. tuberculosis* transmission associated with multiple SNPs in *PhoR* can be attributed to their impact on gene expression, thereby influencing crucial cellular processes including metabolism, virulence, and adaptation in *M. tuberculosis* [14]. Furthermore, several studies have indicated that mutations occurring at specific sites in the *PhoR* gene can affect various biological aspects of *M. tuberculosis*, such as growth, metabolism, and drug resistance, thus indirectly influencing the pathogen’s survival rate and infectivity within the host [15, 16].

The *Rv1027c-Rv1028c* genes in *M. tuberculosis* are predicted to encode the *kdpDE* two-component system, which exhibits a high degree of conservation among bacterial species [17]. This system has been extensively studied and found to regulate virulence and stress resistance in various human pathogens. Our results reveal that multiple SNPs in *kdpD* increase the risk of transmission of *M. tuberculosis*. These SNPs may change the function of *kdpD* protein by affecting the domain of *kdpD*, and trigger the expression regulation of *kdpD*. This regulation of gene expression further promotes the adaptive response of *kdpDE* system [18]. Additionally, research has demonstrated the essential role of *kdpD* in the pathogen’s survival within the host, and a mutant strain with *kdpDE* exhibited a hyper-virulent phenotype in SCID mice [19, 20]. The expression of the *trcR-trcS* two-component system is induced upon the adaptation of the organism to the intracellular milieu and potentially during extracellular replication of *M. tuberculosis* within the liquefaction cavity after rupture of the wall granuloma [21]. Our results revealed that multiple SNPs in *trcS* increased the risk of transmission of *M. tuberculosis.* The *trcS* gene in *M. tuberculosis* serves multiple functions, acting as a regulatory gene encoding a sensor kinase protein involved in the two-component signal transduction system. This system enables the bacterium to detect and respond to environmental changes. Moreover, studies have demonstrated that deletion of the *trcS* gene in this bacterium reduces its survival rate in mouse lungs and increases host clearance [22, 23]. Furthermore, mutations in the *trcS* gene may be associated with drug resistance in *M. tuberculosis* by regulating intracellular metabolic and virulence processes.

Moreover, our research also elucidates the association between SNPs in other two-component system genes and the dissemination of *M.tuberculosis*. These genetic mutations have the potential to alter diverse physiological functions of the bacterium that are intricately linked to its transmission. Mutations of these gene may change various physiological functions of the bacterium related to their transmission. It is worth noting that although we have confirmed the impact of these SNPs on the transmission of *M. tuberculosis*, further research is still needed to determine how these mutations affect the function of the TCSs and the mechanism through which they influence the transmission of *M. tuberculosis*. Additionally, it should be noted that factors influencing the transmission of *M. tuberculosis* are highly complex, involving not only genetic mutations but also various aspects such as the environment, host immune system, and genotype. Therefore, in formulating prevention and treatment strategies, it is necessary to comprehensively consider all possible factors, objectively evaluate their contributions to disease transmission, and thus more effectively control and prevent the occurrence and transmission of tuberculosis.

## Conclusion

The two-component system is a widely distributed signal transduction system in bacteria that regulates a variety of biological processes, including metabolism, virulence, pathogenicity, and adaptation. The SNPs in TCSs gene increase the risk of transmission of *M.tuberculosis*, which reflects the important role of TCSs in the life activities of *M.tuberculosis*. Therefore, in-depth research on the function and regulatory mechanism of these genes can help us better understand the molecular biology characteristics of *M. tuberculosis*, providing new ideas and methods for the prevention and control of tuberculosis. In summary, this study provides new clues for us to understand the transmission mechanism of *M. tuberculosis* and also serves as a reference for related research. In the future, we will further deepen research in this area to provide more effective means for controlling tuberculosis.

## Method

### Sample Collection

A total of 1550 *M. tuberculosis* culture-positive cases were collected from two medical institutions from 2011 to 2018 in China: Shandong Public Health Clinical Research Center (SPHCC) and Weifang Respiratory Clinical Hospital (WRCH). The study did not include *M. tuberculosis* culture-positive cases who had previously undergone evaluation and were subsequently being treated.

### DNA extraction and sequencing

Genomic DNA was extracted from 1447 strains using Cetyltrimethylammonium Bromide (CTAB) and underwent quality control (QC). In total, 103 strains of *M.tuberculosis* were excluded because of improper handling during the DNA extraction and poor quality of extracted DNA. The genomes were sequenced using the Illumina HiSeq 4000 system, and the resulting sequence data were deposited in the National Center for Biotechnology Information (NCBI) under BioProject PRJNA1002108. In addition, this study included 13,267 strains of *M. tuberculosis* from 52 countries and 18 regions around the world [24–32]. We utilized BWA-MEM (version 0.7.17-r1188) to accurately map the reference genome of the standard isolate *M. tuberculosis H37Rv*. Our analysis only included samples exhibiting a coverage rate of 98% or higher and a minimum depth of at least 20% [33]. Finally, a total of 13,531 genomes were analyzed, please refer to Additional file 2: Tables S1-S2 for the specific sample numbers.

### Single nucleotide polymorphism (SNP) analysis

After variant calling using Samclip (version 0.4.0) and SAMtools (version 1.15), we applied further filtering to the resulting variants via Free Bayes (version 1.3.2) and Bcftools (version 1.15.1). We excluded Single Nucleotide Polymorphisms (SNPs) located within repeat regions, including polymorphic GC-rich sequences (PE/PPE genes) and direct repeat SNPs, as well as repeat bases identified through the use of Tandem Repeat Finder (version 4.09) and RepeatMask (version 4.1.2-P1) [34, 35]. Finally, SNP annotation was conducted via SnpEff v 4.1 l, with the resulting output obtained utilizing the Python programming language [36].

### Phylogenetic analysis

**Fig. 3:**
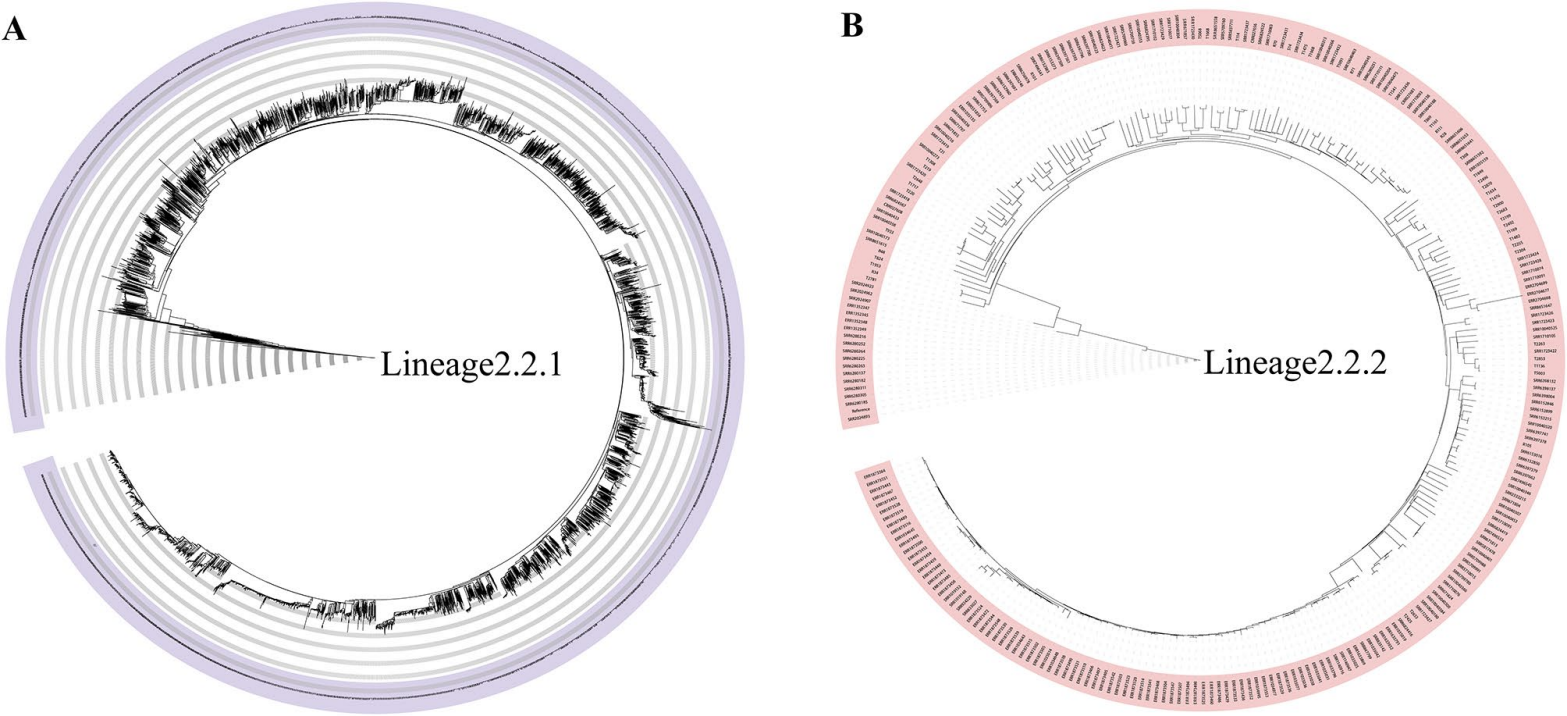
The phylogenetic tree analysis of lineage2.2. (**A**) the phylogenetic tree analysis of lineage2.2.1. (**B**) the phylogenetic tree analysis of lineage2.2.2

The strains were classified into different lineages according to Coll et al. [37](Additional file 2: Tables S1-S2). Construction of the maximum likelihood phylogenetic tree was conducted through the IQ-TREE software package (version 1.6.12), utilizing the JC nucleotide substitution model and gamma model of rate heterogeneity, with 100 bootstrap replicates included [38]. *Mycobacterium canettii CIPT140010059* was deemed to be an outlier. The resultant phylogenetic tree was visualized through the utilization of iTOL (https://itol.embl.de/) (Fig. 3, Additional file 1: Figs. S1–S7).

### Propagation analysis

Cluster analysis was utilized to investigate the influence of two-component system gene mutations on the transmission of *M. tuberculosis* [39]. Based on a previous study [40], we applied clustering to define transmission clusters and used a threshold of less than 25 SNPs. In addition, we chose the threshold of 25 SNPs because our isolates were spread in terms of location and time (1991–2019) and because we were probably missing several intermediary isolates (and cases) in our collection. (Additional file 2: Tables S1-S2). Additionally, according to the classification of transmission clusters by scholars, we also divided transmission clusters into large, medium, or small (large, over 75th percentile; medium, between 25th and 75th percentile; and small, under 25th percentile) [14]. To enhance understanding of the global distribution patterns and conduct an extensive analysis of the transmission dynamics of *M.tuberculosis* strains, we classified them into cross-country and within-country clusters. Furthermore, we categorized the *M. tuberculosis* strains into cross-regional and within-regional clusters based on geographic location utilizing the United Nations standard regions (UN M.49).

### Acquisition of two-component system genes

A total of 45 two-component system genes were obtained according to NCBI and literature search [2, 7, 41]. Python was utilized to detect mutations in genes associated with TCSs (Additional file 2: Table S3).

### Modeling and statistical analysis

Prediction models including gradient boosting decision tree and random forest were established by machine learning using the Scikit-learn Python package. We randomly divided all samples into training and test sets at a ratio of 7:3. Each of the models was evaluated with the metrics of Kappa, sensitivity, specificity, accuracy, positive predictive value (PPV), negative predictive value (NPV), positive likelihood ratio (PLR), negative likelihood ratio (NLR) and area under curve (AUC) [42]. After the model was fitted, we evaluated the importance of the input variables on the model. To enhance the precision of predicting risk factors, we utilized the score to assess the influence of each input feature of the models, and take the intersection of both conditions and obtain the top-performing accessions as the important features [43, 44]. Subsequently, we established the generalized linear mixed model by using the statsmodels.api Python package to further analyze the important features and obtain the final influencing factors. All statistical analyses were performed using SPSS 26.0. All statistical tests were two-tailed, and *P* values less than 0.05 were considered statistically significant.

### Electronic supplementary material

Below is the link to the electronic supplementary material.

Supplementary Material 1: Additional file 2: Tables S1–S3

Supplementary Material 2: Additional file 2: Tables S4–S8

Supplementary Material 3: Additional file 1: Fig. S2

Supplementary Material 4: Additional file 1: Fig. S1

Supplementary Material 5: Additional file 1: Fig. S4

Supplementary Material 6: Additional file 1: Fig. S3

Supplementary Material 7: Additional file 1: Fig. S6

Supplementary Material 8: Additional file 1: Fig. S5

Supplementary Material 9: Additional file 1: Fig. S7

Supplementary Material 10: Additional file 1: Fig. S8

Supplementary Material 11: Additional file 1: Fig. S9

Supplementary Material 12: Additional file 1: Fig. S12

Supplementary Material 13: Additional file 1: Fig. S11

Supplementary Material 14: Additional file 1: Fig. S10

Supplementary Material 15: Additional file 1: Fig. S13

Supplementary Material 16: Additional file 1: Fig. S14

Supplementary Material 17: Additional file 1: Fig. S16

Supplementary Material 18: Additional file 1: Fig. S15

Supplementary Material 19: Additional file 1: Fig. S18

Supplementary Material 20: Additional file 1: Fig. S19

Supplementary Material 21: Additional file 1: Fig. S17

Supplementary Material 22: Additional file 2: Tables S9–S38

Supplementary Material 23: Legends of Additional files

## Data Availability

The whole genome sequences have been submitted to the NCBI under the accession number PRJNA1002108.

## Abbreviations

*M. tuberculosis*: Mycobacterium tuberculosis
TCSs: Two-component systems
WGS: Whole genome sequencing
SPHCC: Shandong Public Health Clinical Research Center
WRCH: Weifang Respiratory Clinical Hospital
CTAB: Cetyltrimethylammonium Bromide
QC: Quality control
SNP: Single nucleotide polymorphism
SNPs: Single nucleotide polymorphisms
NCBI: National Center for Biotechnology Information
PPV: Positive Predictive Value
NPV: Negative Predictive Value
PLR: Positive Likelihood Ratio
NLR: Negative Likelihood Ratio
AUC: Area Under Curve
