# Correction: Association between two-component systems gene mutation and *Mycobacterium tuberculosis* transmission revealed by whole genome sequencing

**DOI:** 10.1186/s12864-023-09914-0

**Published:** 2024-01-08

**Authors:** Yameng Li, Xianglong Kong, Yifan Li, Ningning Tao, Yawei Hou, Tingting Wang, Yingying Li, Qilin Han, Yao Liu, Huaichen Li

**Affiliations:** 1 Shandong University of Traditional Chinese Medicine, Jinan, Shandong 250014 People’s Republic of China; 2 Artificial Intelligence Institute, Qilu University of Technology (Shandong Academy of Sciences), Jinan, Shandong 250011 People’s Republic of China; 3 Department of Respiratory and Critical Care Medicine, The Third Affiliated Hospital of Shandong First Medical University (Affiliated Hospital of Shandong Academy of Medical Sciences), Jinan, Shandong 250031 People’s Republic of China; 4 Department of Respiratory and Critical Care Medicine, Shandong Provincial Hospital, Shandong University, Shandong Provincial Hospital Affiliated to Shandong First Medical University, Jingwuweiqi Road, Huaiyin District, Jinan, Shandong 250021 People’s Republic of China; 5 Shandong First Medical University & Shandong Academy of Medical Sciences, Jinan, Shandong 250117 People’s Republic of China

**Correction to: BMC Genomics (2023) 24:718**

10.1186/s12864-023-09788-2

Following publication of the original article [1], it was reported that there was an error in affiliation 1 and that the Background section of the Abstract had to be revised.

## The incorrect version of affiliation 1 was:

Department of Chinese Medicine Integrated with Western Medicine, College of Traditional Chinese Medicine, Shandong University of Traditional Chinese Medicine, 16,369 Jingshi Road, Lixia District, Jinan, Shandong, 250355, People’s Republic of China.

## The corrected version of affiliation 1 is:

Shandong University of Traditional Chinese Medicine, Jinan, Shandong, 250014, People’s Republic of China.

## The incorrect background text was:

Two-component systems (TCSs) assume a pivotal function in *Mycobacterium tuberculosis* (*M.tuberculosis*) growth. However, the exact regulatory mechanism of this system needs to be elucidated, and only a few studies have investigated the effect of gene mutations within TCSs on *M.tuberculosis* transmission. This research explored the relationship between TCSs gene mutation and the global transmission of (*M.tuberculosis*).

## The corrected background text is:

Two-component systems (TCSs) play a crucial role in the growth of *Mycobacterium tuberculosis* (*M. tuberculosis*). However, the precise regulatory mechanism of their contribution remains to be elucidated, and only a limited number of studies have investigated the impact of gene mutations within TCSs on the transmission of *M. tuberculosis*. Therefore, this study aims to explore the relationship between TCSs gene mutation and the global transmission of *M. tuberculosis*.

The original article has been updated.
