# Supplementary figures and images for: Association between two-component systems gene mutation and Mycobacterium tuberculosis transmission revealed by whole genome sequencing
Source: BMC Genomics. 2023 Nov 28;24:718. doi: 10.1186/s12864-023-09788-2 (PMC10683263; doi:10.1186/s12864-023-09788-2)

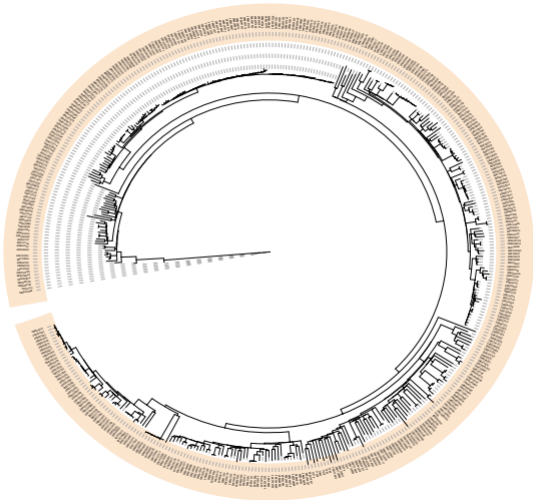

Supplement: Supplementary file 3 — Supplementary Material 3: Additional file 1: Fig. S2 [file 12864_2023_9788_MOESM3_ESM.pdf]

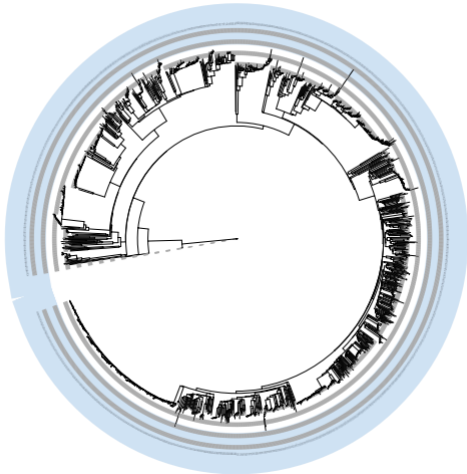

Supplement: Supplementary file 4 — Supplementary Material 4: Additional file 1: Fig. S1 [file 12864_2023_9788_MOESM4_ESM.pdf]

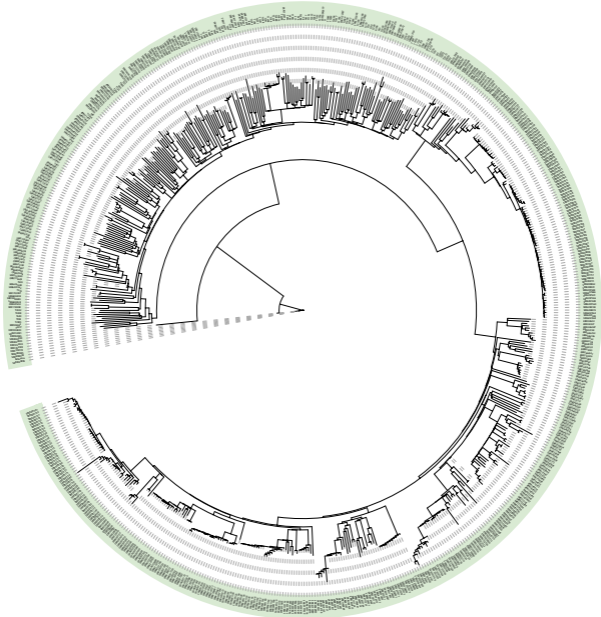

Supplement: Supplementary file 5 — Supplementary Material 5: Additional file 1: Fig. S4 [file 12864_2023_9788_MOESM5_ESM.pdf]

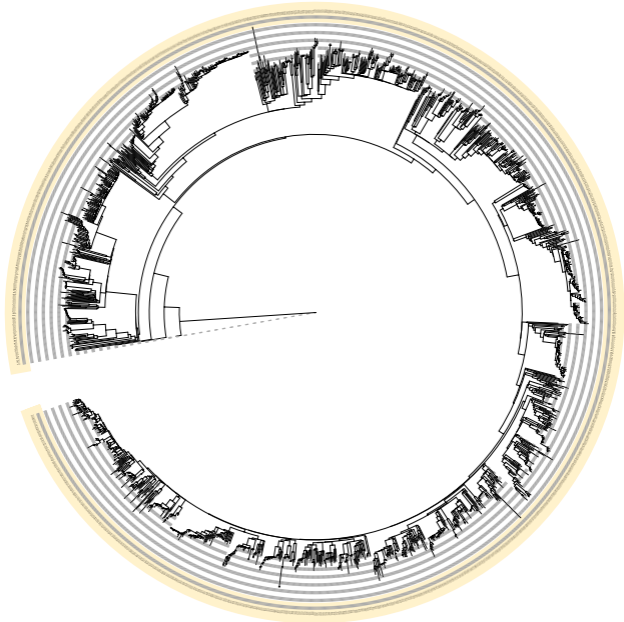

Supplement: Supplementary file 6 — Supplementary Material 6: Additional file 1: Fig. S3 [file 12864_2023_9788_MOESM6_ESM.pdf]

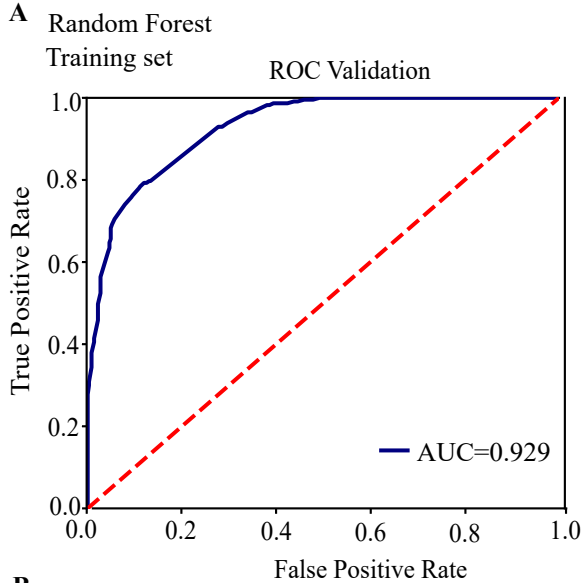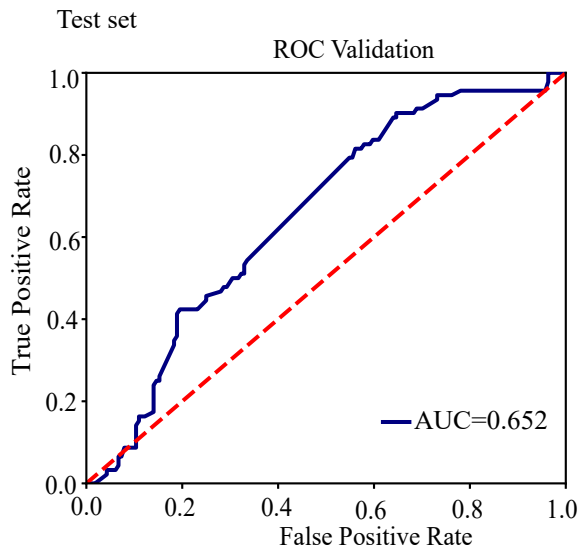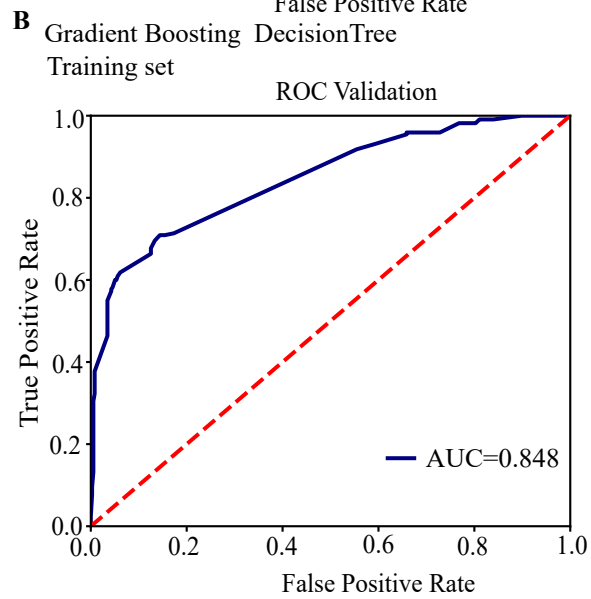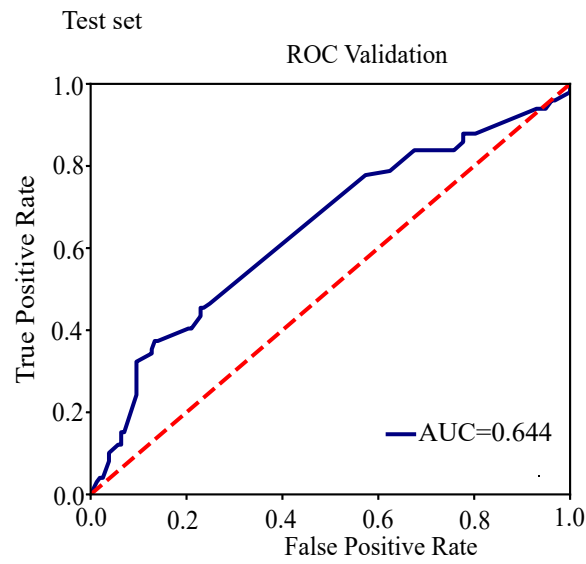

Supplement: Supplementary file 7 — Supplementary Material 7: Additional file 1: Fig. S6 [file 12864_2023_9788_MOESM7_ESM.pdf]

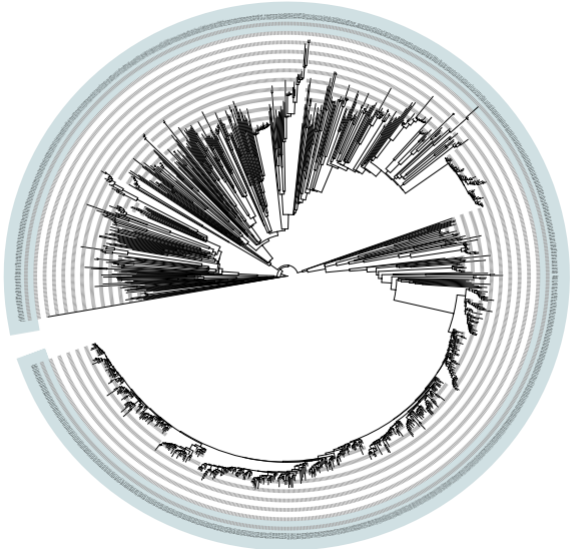

Supplement: Supplementary file 8 — Supplementary Material 8: Additional file 1: Fig. S5 [file 12864_2023_9788_MOESM8_ESM.pdf]

**A** Random Forest  
Training set

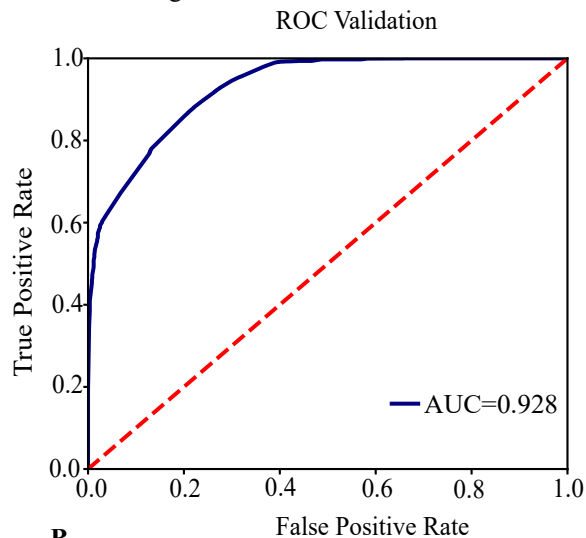

Test set

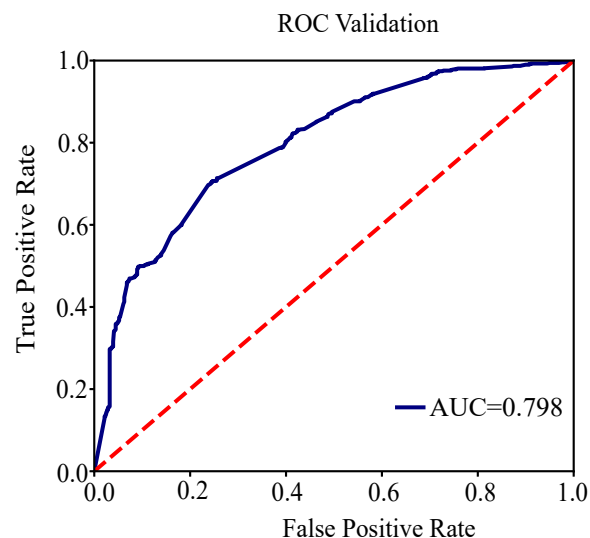

**B** Gradient Boosting Decision Tree  
Training set

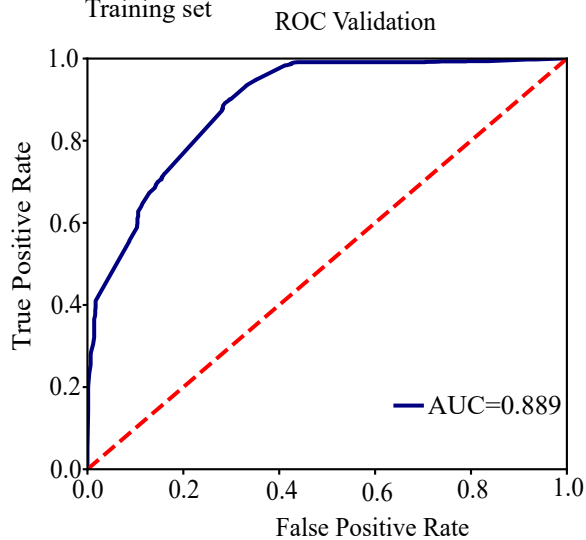

Test set

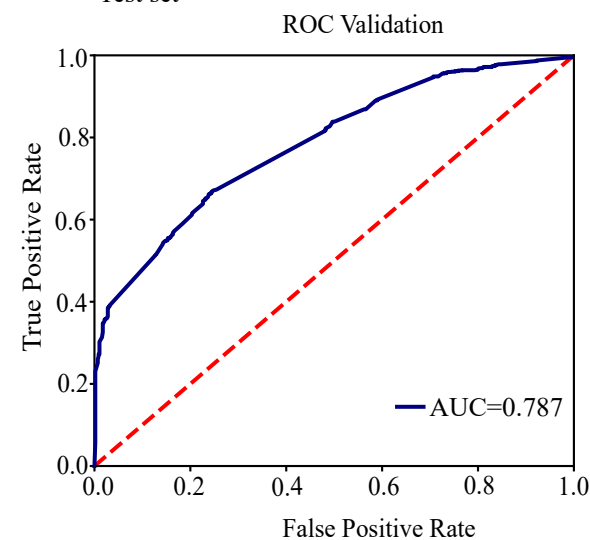

Supplement: Supplementary file 10 — Supplementary Material 10: Additional file 1: Fig. S8 [file 12864_2023_9788_MOESM10_ESM.pdf]

**A** Random Forest  
Training set

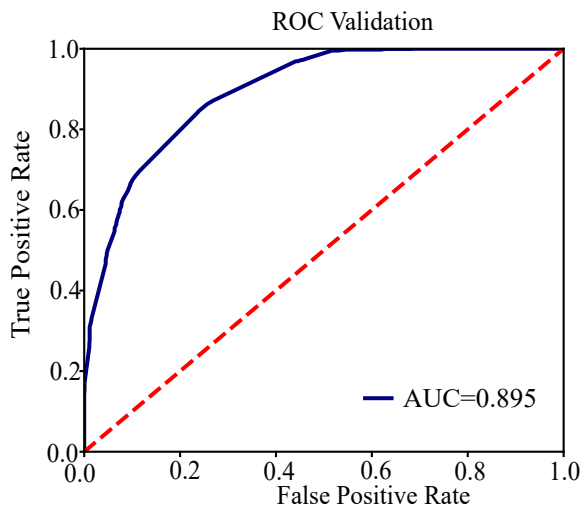

Test set

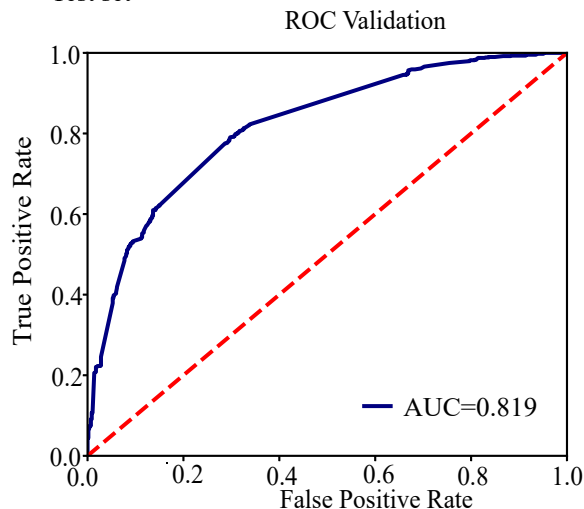

**B** Gradient Boosting Decision Tree  
Training set

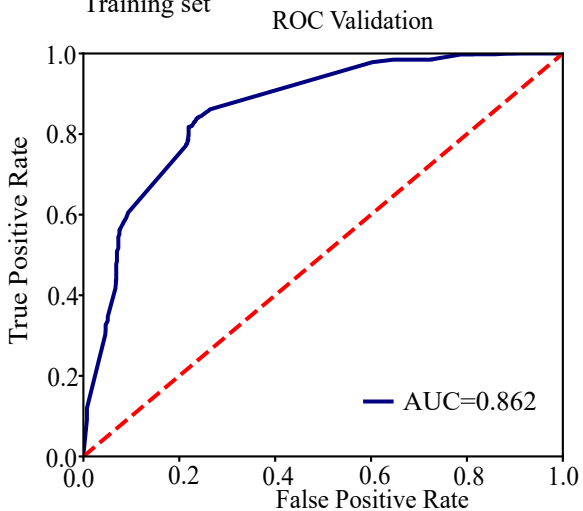

Test set

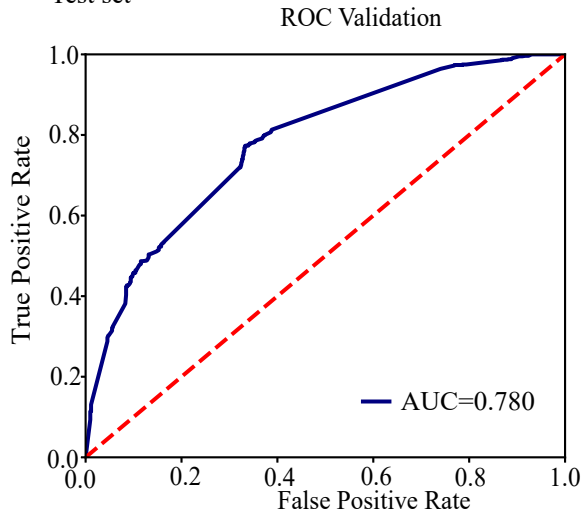

Supplement: Supplementary file 11 — Supplementary Material 11: Additional file 1: Fig. S9 [file 12864_2023_9788_MOESM11_ESM.pdf]

**A** Random Forest  
Training set

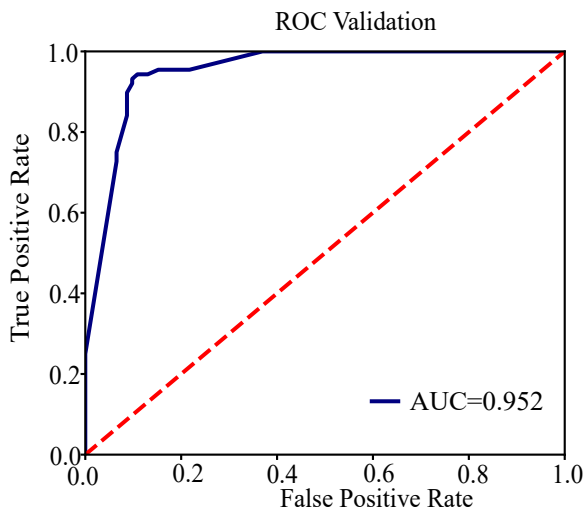

Test set

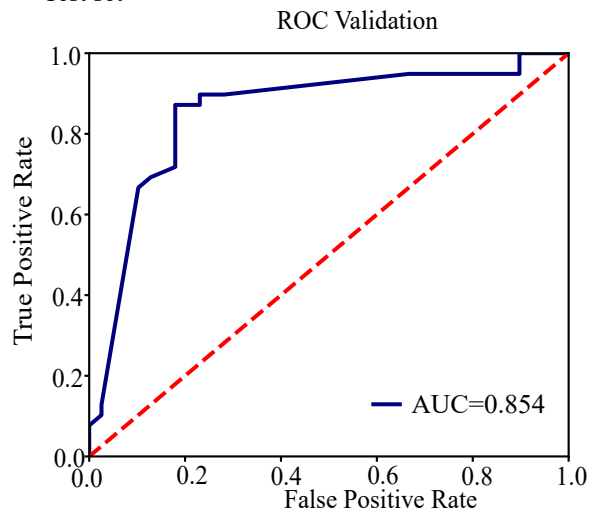

**B** Gradient Boosting Decision Tree  
Training set

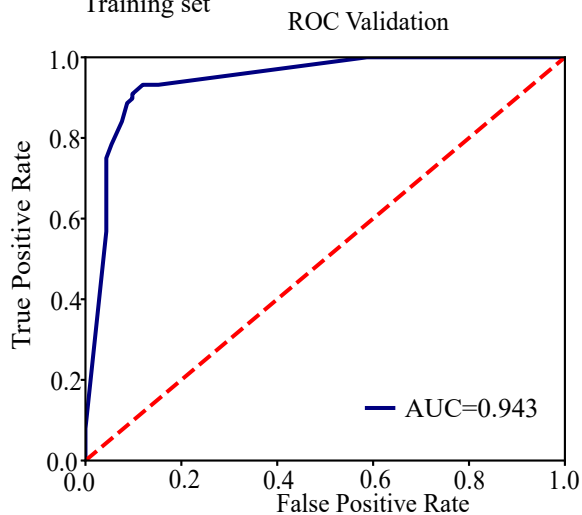

Test set

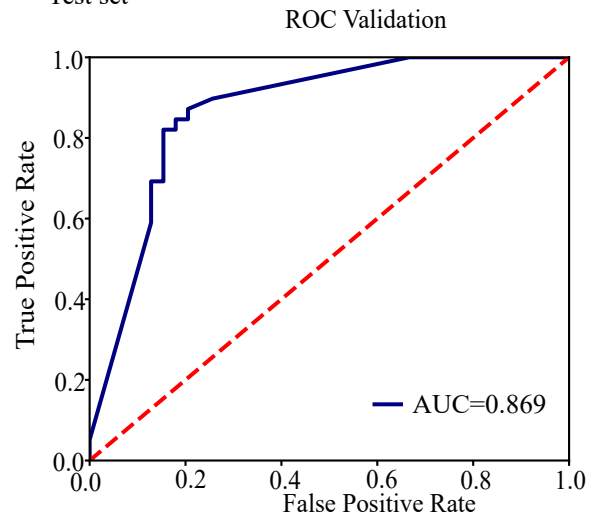

Supplement: Supplementary file 14 — Supplementary Material 14: Additional file 1: Fig. S10 [file 12864_2023_9788_MOESM14_ESM.pdf]

**A** Random Forest  
Training set

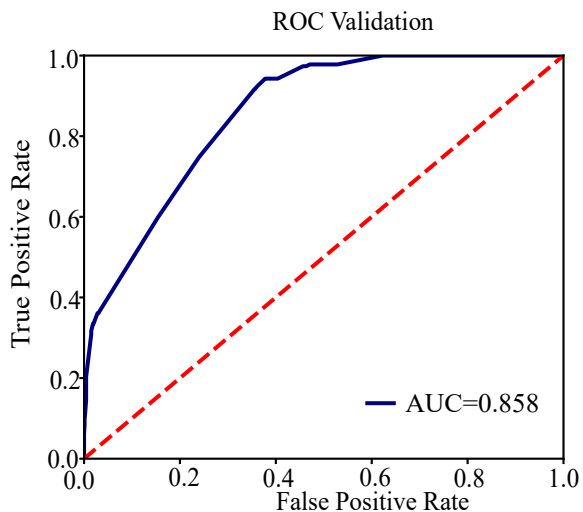

Test set

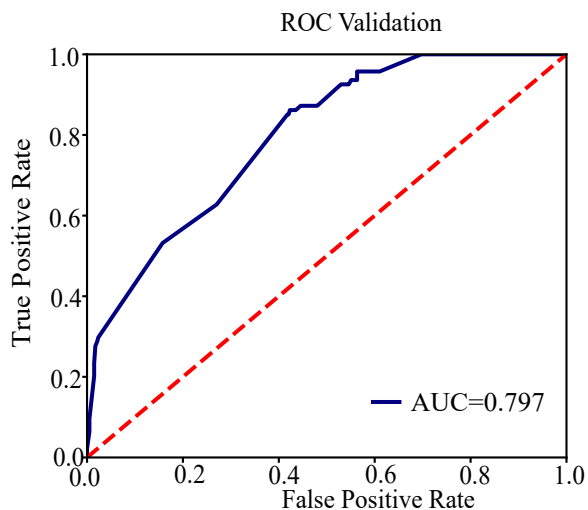

**B** Gradient Boosting Decision Tree  
Training set

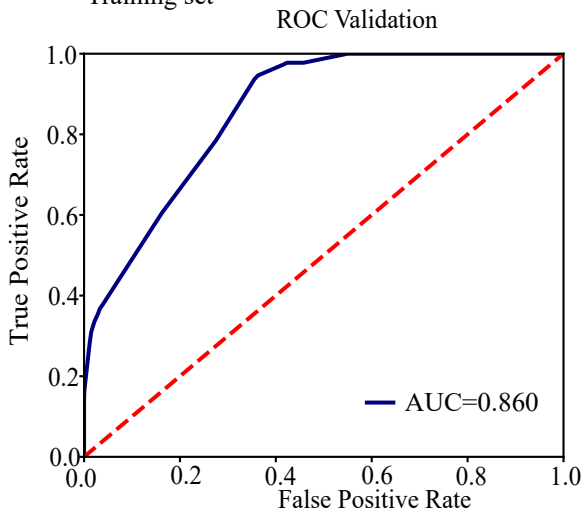

Test set

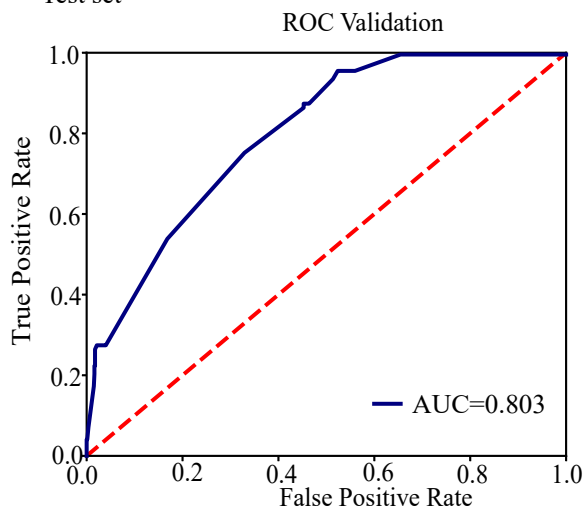

Supplement: Supplementary file 19 — Supplementary Material 19: Additional file 1: Fig. S18 [file 12864_2023_9788_MOESM19_ESM.pdf]
