# Supplementary material for: Association between two-component systems gene mutation and Mycobacterium tuberculosis transmission revealed by whole genome sequencing
Source: BMC Genomics. 2023 Nov 28;24:718. doi: 10.1186/s12864-023-09788-2 (PMC10683263; doi:10.1186/s12864-023-09788-2)
Supplement: Supplementary file 15 — Supplementary Material 15: Additional file 1: Fig. S13 [file 12864_2023_9788_MOESM15_ESM.pdf]

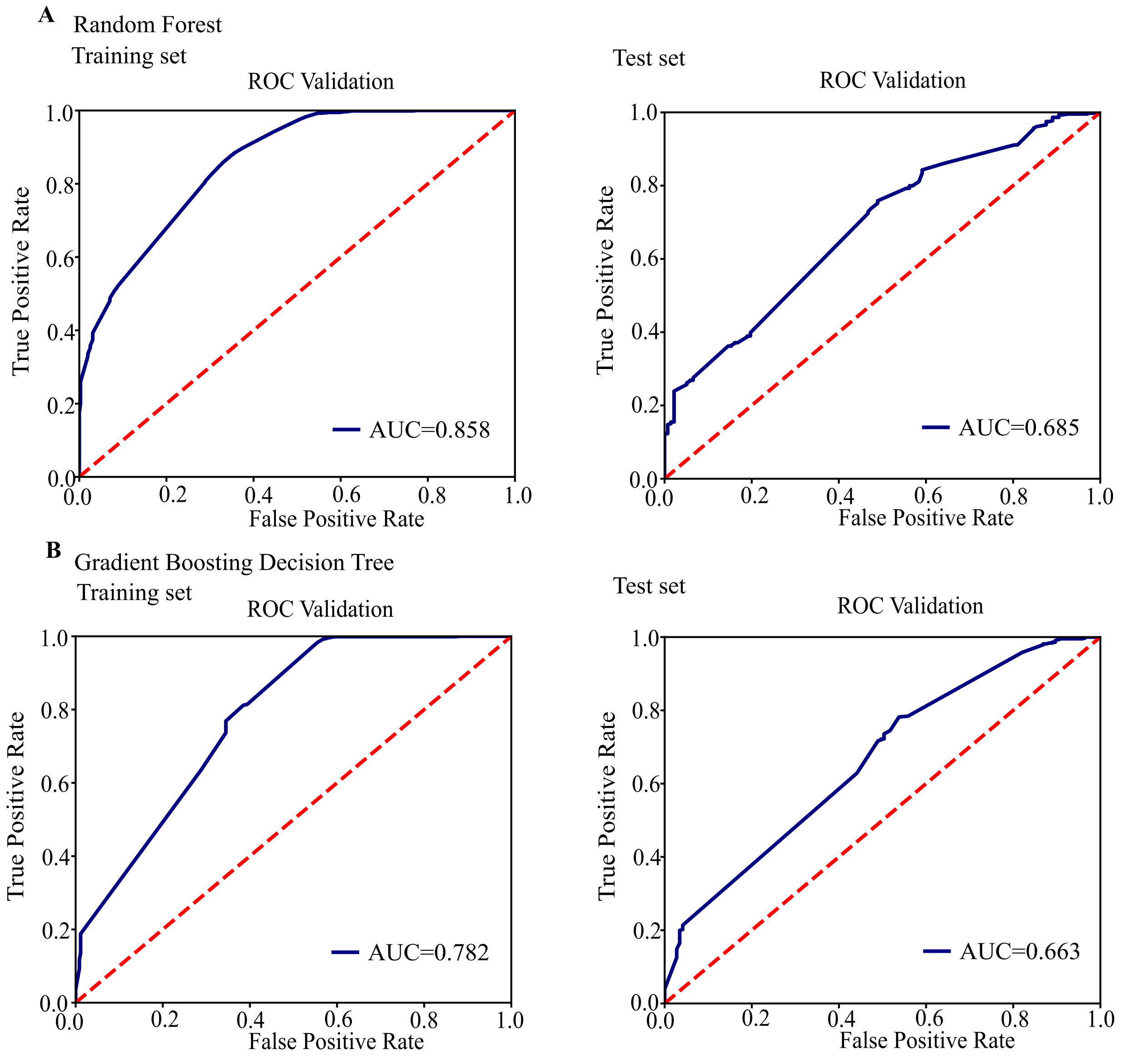
A
Random Forest
Training set
ROC Validation
True Positive Rate
False Positive Rate
AUC=0.858
Test set
ROC Validation
True Positive Rate
False Positive Rate
AUC=0.685
B
Gradient Boosting Decision Tree
Training set
ROC Validation
True Positive Rate
False Positive Rate
AUC=0.782
Test set
ROC Validation
True Positive Rate
False Positive Rate
AUC=0.663
0.0
0.2
0.4
0.6
0.8
1.0
