# Supplementary material for: Association between two-component systems gene mutation and Mycobacterium tuberculosis transmission revealed by whole genome sequencing
Source: BMC Genomics. 2023 Nov 28;24:718. doi: 10.1186/s12864-023-09788-2 (PMC10683263; doi:10.1186/s12864-023-09788-2)
Supplement: Supplementary file 22 — Supplementary Material 22: Additional file 2: Tables S9–S38 [file 12864_2023_9788_MOESM22_ESM.docx]

**Table S9.** The performance of various models for discriminating clustered strains from non-clustered strains in the lineage1 cohort.

| **Parameters** | **Training set**  **(n=595, 218 clustered strains,**  **377 non-clustered strains)** | | **Test set**  **(n=256, 101 clustered strains,**  **155 non-clustered strains)** | |
| --- | --- | --- | --- | --- |
|  | **Random Forest** | **Gradient Boosting Decision Tree** | **Random Forest** | **Gradient Boosting Decision Tree** |
| Kappa | 0.676 | 0.561 | 0.206 | 0.252 |
| AUC  (95% CI) | 0.929  (0.908, 0.950) | 0.848  (0.819, 0.877) | 0.652  (0.594, 0.710) | 0.644  (0.585, 0.703) |
| Sensitivity  (95% CI) | 0.718  (0.682, 0.754) | 0.550  (0.510, 0.590) | 0.424  (0.363, 0.485) | 0.323  (0.266, 0.380) |
| Specificity  (95% CI) | 0.935  (0.915, 0.955) | 0.965  (0.950, 0.980) | 0.774  (0.723, 0.825) | 0.904  (0.868, 0.940) |
| PPV  (95% CI) | 0.872  (0.845, 0.899) | 0.903  (0.879, 0.927) | 0.513  (0.452, 0.574) | 0.681  (0.624, 0.738) |
| NPV  (95% CI) | 0.843  (0.814, 0.872) | 0.785  (0.752, 0.818) | 0.706  (0.65, 0.762) | 0.679  (0.622, 0.736) |
| PLR  (95% CI) | 5.557  (5.507, 5.607) | 4.205  (4.131, 4.279) | 1.743  (1.659, 1.827) | 2.124  (2.015, 2.233) |
| NIR  (95% CI) | 0.180  (-0.031, 0.391) | 0.238  (-0.009, 0.485) | 0.574  (0.429, 0.719) | 0.471  (0.279, 0.663) |
| Accuracy  (95% CI) | 0.852  (0.823, 0.881) | 0.812  (0.781, 0.843) | 0.648  (0.589, 0.707) | 0.680  (0.623, 0.737) |

AUC, area under the curve; PPV, positive predictive value; NPV, negative predictive value; PLR, positive likelihood ratio; NLR, negative likelihood ratio; CI, confidence.

**Table S10.** The performance of various models for discriminating clustered strains from non-clustered strains in the lineage3 cohort.

| **Parameters** | **Training set**  **(n=679, 334 clustered strains,**  **345 non-clustered strains)** | | **Test set**  **(n=291, 134 clustered strains,**  **157 non-clustered strains)** | |
| --- | --- | --- | --- | --- |
|  | **Random Forest** | **Gradient Boosting Decision Tree** | **Random Forest** | **Gradient Boosting Decision Tree** |
| Kappa | 0.679 | 0.620 | 0.448 | 0.558 |
| AUC  (95% CI) | 0.922  (0.902, 0.942) | 0.873  (0.848, 0.898) | 0.811  (0.766, 0.856) | 0.814  (0.769, 0.859) |
| Sensitivity  (95% CI) | 0.879  (0.854, 0.904) | 0.818  (0.789, 0.847) | 0.819  (0.775, 0.863) | 0.754  (0.705, 0.803) |
| Specificity  (95% CI) | 0.802  (0.772, 0.832) | 0.802  (0.772, 0.832) | 0.634  (0.579, 0.689) | 0.804  (0.758, 0.850) |
| PPV  (95% CI) | 0.830  (0.802, 0.858) | 0.796  (0.766, 0.826) | 0.681  (0.627, 0.735) | 0.776  (0.728, 0.824) |
| NPV  (95% CI) | 0.875  (0.850, 0.90) | 0.824  (0.795, 0.853) | 0.795  (0.749, 0.841) | 0.783  (0.736, 0.830) |
| PLR  (95% CI) | 6.462  (6.428, 6.496) | 4.513  (4.468, 4.558) | 3.263  (3.197, 3.329) | 3.584  (3.504, 3.664) |
| NIR  (95% CI) | 0.155  (-0.002, 0.312) | 0.222  (0.07, 0.374) | 0.306  (0.132, 0.480) | 0.279  (0.058, 0.50) |
| Accuracy  (95% CI) | 0.839  (0.811, 0.867) | 0.810  (0.78, 0.84) | 0.722  (0.671, 0.773) | 0.780  (0.732, 0.828) |

AUC, area under the curve; PPV, positive predictive value; NPV, negative predictive value; PLR, positive likelihood ratio; NLR, negative likelihood ratio; CI, confidence.

**Table S11.** The performance of various models for discriminating clustered strains from non-clustered strains in the lineage4 cohort.

| **Parameters** | **Training set**  **(n=4547, 3188 clustered strains,**  **1359 non-clustered strains)** | | **Test set**  **(n=1950, 1386 clustered strains,**  **564 non-clustered strains)** | |
| --- | --- | --- | --- | --- |
|  | **Random Forest** | **Gradient Boosting Decision Tree** | **Random Forest** | **Gradient Boosting Decision Tree** |
| Kappa | 0.672 | 0.632 | 0.325 | 0.261 |
| AUC  (95% CI) | 0.928  (0.92, 0.936) | 0.889  (0.880, 0.898) | 0.798  (0.780, 0.816) | 0.785  (0.767, 0.803) |
| Sensitivity  (95% CI) | 0.985  (0.981, 0.989) | 0.991  (0.988, 0.994) | 0.968  (0.96, 0.976) | 0.977  (0.97, 0.984) |
| Specificity  (95% CI) | 0.621  (0.607, 0.635) | 0.576  (0.562, 0.59) | 0.297  (0.277, 0.317) | 0.262  (0.242, 0.282) |
| PPV  (95% CI) | 0.858  (0.848, 0.868) | 0.844  (0.833, 0.855) | 0.774  (0.755, 0.793) | 0.755  (0.736, 0.774) |
| NPV  (95% CI) | 0.947  (0.940, 0.954) | 0.965  (0.960, 0.970) | 0.787  (0.769, 0.805) | 0.747  (0.728, 0.766) |
| PLR  (95% CI) | 16.329  (16.321, 16.337) | 23.893  (23.887, 23.899) | 3.629  (3.599,3.659) | 2.988  (2.953, 3.023) |
| NIR  (95% CI) | 0.061  (-0.011, 0.133) | 0.042  (-0.026, 0.11) | 0.276  (0.191, 0.361) | 0.335  (0.254, 0.416) |
| Accuracy  (95% CI) | 0.876  (0.866, 0.886) | 0.865  (0.855, 0.875) | 0.775  (0.756, 0.794) | 0.754, (0.735, 0.773) |

AUC, area under the curve; PPV, positive predictive value; NPV, negative predictive value; PLR, positive likelihood ratio; NLR, negative likelihood ratio; CI, confidence.

**Table S12.** The performance of various models for discriminating clustered strains from non-clustered strains in the lineage2.2.1 cohort.

| **Parameters** | **Training set**  **(n=3382, 2031 clustered strains,**  **1351 non-clustered strains)** | | **Test set**  **(n=1450, 827 clustered strains,**  **623 non-clustered strains)** | |
| --- | --- | --- | --- | --- |
|  | **Random Forest** | **Gradient Boosting Decision Tree** | **Random Forest** | **Gradient Boosting Decision Tree** |
| Kappa | 0.611 | 0.602 | 0.487 | 0.433 |
| AUC  (95% CI) | 0.895  (0.885, 0.905) | 0.862  (0.85, 0.874) | 0.819  (0.799, 0.839) | 0.780  (0.759, 0.801) |
| Sensitivity  (95% CI) | 0.868  (0.857, 0.879) | 0.839  (0.827, 0.851) | 0.809  (0.789, 0.829) | 0.788  (0.767, 0.809) |
| Specificity  (95% CI) | 0.736  (0.721, 0.751) | 0.764  (0.750, 0.778) | 0.676  (0.652, 0.70) | 0.641  (0.616, 0.666) |
| PPV  (95% CI) | 0.823  (0.810, 0.836) | 0.841  (0.829, 0.853) | 0.794  (0.773, 0.815) | 0.748  (0.726, 0.770) |
| NPV  (95% CI) | 0.798  (0.784, 0.812) | 0.761  (0.747, 0.775) | 0.697  (0.673, 0.721) | 0.691  (0.667, 0.715) |
| PLR  (95% CI) | 4.066  (4.042, 4.090) | 3.516  (3.487, 3.545) | 2.617  (2.566, 2.668) | 2.418  (2.370, 2.466) |
| NIR  (95% CI) | 0.246  (0.172, 0.32) | 0.284  (0.205, 0.363) | 0.382  (0.278, 0.486) | 0.414  (0.321, 0.507) |
| Accuracy  (95% CI) | 0.813  (0.80, 0.826) | 0.809  (0.796, 0.822) | 0.757  (0.735, 0.779) | 0.726  (0.703, 0.749) |

AUC, area under the curve; PPV, positive predictive value; NPV, negative predictive value; PLR, positive likelihood ratio; NLR, negative likelihood ratio; CI, confidence.

**Table S13.** The performance of various models for discriminating clustered strains from non-clustered strains in the lineage2.2.2 cohort.

| **Parameters** | **Training set**  **(n=180, 91 clustered strains,**  **89 non-clustered strains)** | | **Test set**  **(n=78, 36 clustered strains,**  **42 non-clustered strains)** | |
| --- | --- | --- | --- | --- |
|  | **Random Forest** | **Gradient Boosting Decision Tree** | **Random Forest** | **Gradient Boosting Decision Tree** |
| Kappa | 0.833 | 0.811 | 0.692 | 0.667 |
| AUC  (95% CI) | 0.952  (0.921, 0.983) | 0.943  (0.909, 0.977) | 0.854  (0.776, 0.932) | 0.869  (0.794, 0.944) |
| Sensitivity  (95% CI) | 0.943  (0.909, 0.977) | 0.909  (0.867, 0.951) | 0.872  (0.798, 0.946) | 0.872  (0.798, 0.946) |
| Specificity  (95% CI) | 0.891  (0.845, 0.937) | 0.902  (0.859, 0.945) | 0.821  (0.736, 0.906) | 0.795  (0.705, 0.885) |
| PPV  (95% CI) | 0.892  (0.847, 0.937) | 0.899  (0.855, 0.943) | 0.829  (0.745, 0.913) | 0.810  (0.723, 0.897) |
| NPV  (95% CI) | 0.943  (0.909, 0.977) | 0.912  (0.871, 0.953) | 0.865  (0.789, 0.941) | 0.861  (0.784, 0.938) |
| PLR  (95% CI) | 15.529  (15.486,15.572) | 10.225  (10.165, 10.285) | 6.137  (6.025, 6.249) | 5.829  (5.718, 5.940) |
| NIR  (95% CI) | 0.064  (-0.359, 0.487) | 0.098  (-0.340, 0.536) | 0.163  (-0.334, 0.660) | 0.172  (-0.295, 0.639) |
| Accuracy  (95% CI) | 0.917  (0.877, 0.957) | 0.906  (0.863, 0.949) | 0.846  (0.766, 0.926) | 0.833  (0.750, 0.916) |

AUC, area under the curve; PPV, positive predictive value; NPV, negative predictive value; PLR, positive likelihood ratio; NLR, negative likelihood ratio; CI, confidence.

**Table S14.** The performance of various models for discriminating clustered strains from non-clustered strains in the lineage4.1 cohort.

| **Parameters** | **Training set**  **(n=1129, 845 clustered strains,**  **284 non-clustered strains)** | | **Test set**  **(n=485, 356 clustered strains,**  **129 non-clustered strains)** | |
| --- | --- | --- | --- | --- |
|  | **Random Forest** | **Gradient Boosting Decision Tree** | **Random Forest** | **Gradient Boosting Decision Tree** |
| Kappa | 0.624 | 0.673 | 0.223 | 0.223 |
| AUC  (95% CI) | 0.932  (0.917, 0.947) | 0.948  (0.935, 0.961) | 0.848  (0.816, 0.880) | 0.834  (0.801, 0.867) |
| Sensitivity  (95% CI) | 0.993  (0.988, 0.998) | 0.995  (0.991, 0.999) | 0.987  (0.977, 0.997) | 0.981  (0.969, 0.993) |
| Specificity  (95% CI) | 0.547  (0.518, 0.576) | 0.592  (0.563, 0.621) | 0.173  (0.139, 0.207) | 0.185  (0.150, 0.220) |
| PPV  (95% CI) | 0.853  (0.832, 0.874) | 0.874  (0.855, 0.893) | 0.814  (0.779, 0.849) | 0.787  (0.751, 0.823) |
| NPV  (95% CI) | 0.966  (0.955, 0.977) | 0.978  (0.969, 0.987) | 0.783  (0.746, 0.820) | 0.759  (0.721, 0.797) |
| PLR  (95% CI) | 24.886  (24.875,24.897) | 38.885  (38.876,38.894) | 3.744  (3.678, 3.810) | 3.262  (3.192, 3.332) |
| NIR  (95% CI) | 0.040  (-0.102, 0.182) | 0.026  (-0.129, 0.181) | 0.267  (0.077, 0.457) | 0.307  (0.131, 0.483) |
| Accuracy  (95% CI) | 0.871  (0.851, 0.891) | 0.890  (0.872, 0.908) | 0.812  (0.777, 0.847) | 0.786  (0.749, 0.823) |

AUC, area under the curve; PPV, positive predictive value; NPV, negative predictive value; PLR, positive likelihood ratio; NLR, negative likelihood ratio; CI, confidence.

**Table S15.** The performance of various models for discriminating clustered strains from non-clustered strains in the lineage4.2 cohort.

| **Parameters** | **Training set**  **(n=298, 182 clustered strains,**  **116 non-clustered strains)** | | **Test set**  **(n=129, 86 clustered strains,**  **43 non-clustered strains)** | |
| --- | --- | --- | --- | --- |
|  | **Random Forest** | **Gradient Boosting Decision Tree** | **Random Forest** | **Gradient Boosting Decision Tree** |
| Kappa | 0.713 | 0.704 | 0.332 | 0.436 |
| AUC  (95% CI) | 0.946  (0.920, 0.972) | 0.945  (0.919, 0.973) | 0.758  (0.684, 0.832) | 0.818  (0.751, 0.885) |
| Sensitivity  (95% CI) | 0.884  (0.848, 0.920) | 0.885  (0.849, 0.921) | 0.785  (0.714, 0.856) | 0.860  (0.80, 0.920) |
| Specificity  (95% CI) | 0.835  (0.793, 0.877) | 0.819  (0.775, 0.963) | 0.540  (0.454, 0.626`) | 0.558  (0.472, 0.644) |
| PPV  (95% CI) | 0.903  (0.869, 0.937) | 0.885  (0.849, 0.921) | 0.729  (0.652, 0.806) | 0.796  (0.726, 0.866) |
| NPV  (95% CI) | 0.805  (0.760, 0.850) | 0.819  (0.775, 0.863) | 0.614  (0.530, 0.698) | 0.667  (0.586, 0.748) |
| PLR  (95% CI) | 4.637  (4.542, 4.732) | 4.886  (4.802, 4.970) | 1.888  (1.687, 2.089) | 2.387  (2.197, 2.577) |
| NIR  (95% CI) | 0.216  (-0.132, 0.564) | 0.205  (-0.112, 0.522) | 0.530  (0.230, 0.830) | 0.419  (0.068, 0.770) |
| Accuracy  (95% CI) | 0.866  (0.827, 0.905) | 0.859  (0.819, 0.899) | 0.690  (0.610, 0.770) | 0.760  (0.686, 0.834) |

AUC, area under the curve; PPV, positive predictive value; NPV, negative predictive value; PLR, positive likelihood ratio; NLR, negative likelihood ratio; CI, confidence.

**Table S16.** The performance of various models for discriminating clustered strains from non-clustered strains in the lineage4.3 cohort.

| **Parameters** | **Training set**  **(n=1343, 992 clustered strains,**  **351 non-clustered strains)** | | **Test set**  **(n=576, 434 clustered strains,**  **142 non-clustered strains)** | |
| --- | --- | --- | --- | --- |
|  | **Random Forest** | **Gradient Boosting Decision Tree** | **Random Forest** | **Gradient Boosting Decision Tree** |
| Kappa | 0.538 | 0.505 | 0.125 | 0.125 |
| AUC  (95% CI) | 0.858  (0.839, 0.877) | 0.782  (0.760, 0.804) | 0.685  (0.647, 0.723) | 0.663  (0.624, 0.702) |
| Sensitivity  (95% CI) | 0.991  (0.986,0.996) | 0.998  (0.996,1.0) | 0.986  (0.976, 0.996) | 0.986  (0.976, 0.996) |
| Specificity  (95% CI) | 0.458  (0.431, 0.485) | 0.411  (0.385, 0.437) | 0.102  (0.077, 0.127) | 0.103  (0.078, 0.128) |
| PPV  (95% CI) | 0.835  (0.815, 0.855) | 0.829  (0.809, 0.849) | 0.779  (0.745, 0.813) | 0.766  (0.731, 0.801) |
| NPV  (95% CI) | 0.948  (0.936, 0.960) | 0.986  (0.980, 0.992) | 0.700  (0.663, 0.737) | 0.714  (0.677, 0.751) |
| PLR  (95% CI) | 15.961  (15.948,15.974) | 60.094  (60.088,60.10) | 2.596  (2.518, 2.674) | 2.680  (2.607, 2.753) |
| NIR  (95% CI) | 0.063  (-0.059, 0.185) | 0.017  (-0.102, 0.136) | 0.385  (0.227, 0.543) | 0.373  (0.220, 0.526) |
| Accuracy  (95% CI) | 0.850  (0.831, 0.869) | 0.846  (0.827, 0.865) | 0.776  (0.742, 0.810) | 0.764  (0.729, 0.799) |

AUC, area under the curve; PPV, positive predictive value; NPV, negative predictive value; PLR, positive likelihood ratio; NLR, negative likelihood ratio; CI, confidence.

**Table S17.** The performance of various models for discriminating clustered strains from non-clustered strains in the lineage4.4 cohort.

| **Parameters** | **Training set**  **(n=338, 267 clustered strains,**  **171 non-clustered strains)** | | **Test set**  **(n=188, 125 clustered strains,**  **63 non-clustered strains)** | |
| --- | --- | --- | --- | --- |
|  | **Random Forest** | **Gradient Boosting Decision Tree** | **Random Forest** | **Gradient Boosting Decision Tree** |
| Kappa | 0.760 | 0.818 | 0.367 | 0.390 |
| AUC  (95% CI) | 0.955  (0.936, 0.974) | 0.974  (0.959, 0.989) | 0.866  (0.817, 0.915) | 0.814  (0.758, 0.870) |
| Sensitivity  (95% CI) | 0.954  (0.934, 0.974) | 0.970  (0.954, 0.986) | 0.964  (0.937, 0.991) | 0.879  (0.832, 0.926) |
| Specificity  (95% CI) | 0.785  (0.747, 0.823) | 0.829  (0.794, 0.864) | 0.368  (0.299, 0.437) | 0.484  (0.413, 0.555) |
| PPV  (95% CI) | 0.887  (0.857, 0.917) | 0.900  (0.872, 0.928) | 0.692  (0.626, 0.758) | 0.768  (0.708, 0.828) |
| NPV  (95% CI) | 0.905  (0.878, 0.932) | 0.946  (0.925, 0.967) | 0.875  (0.828, 0.922) | 0.674  (0.607, 0.741) |
| PLR  (95% CI) | 9.348  (9.308,9.388) | 16.756  (16.729, 16.783) | 5.538  (5.481, 5.595) | 2.354  (2.207, 2.501) |
| NIR  (95% CI) | 0.107  (-0.157, 0.371) | 0.060  (-0.222, 0.342) | 0.181  (-0.045, 0.407) | 0.425  (-0.155, 0.695) |
| Accuracy  (95% CI) | 0.893  (0.864, 0.922) | 0.916  (0.890, 0.942) | 0.723  (0.659, 0.787) | 0.745  (0.683, 0.807) |

AUC, area under the curve; PPV, positive predictive value; NPV, negative predictive value; PLR, positive likelihood ratio; NLR, negative likelihood ratio; CI, confidence.

**Table S18.** The performance of various models for discriminating clustered strains from non-clustered strains in the lineage4.8 cohort.

| **Parameters** | **Training set**  **(n=760, 580 clustered strains,**  **180 non-clustered strains)** | | **Test set**  **(n=326, 248 clustered strains,**  **78 non-clustered strains)** | |
| --- | --- | --- | --- | --- |
|  | **Random Forest** | **Gradient Boosting Decision Tree** | **Random Forest** | **Gradient Boosting Decision Tree** |
| Kappa | 0.713 | 0.763 | 0.282 | 0.248 |
| AUC  (95% CI) | 0.956  (0.941, 0.971) | 0.968  (0.955, 0.981) | 0.869  (0.832, 0.906) | 0.867  (0.83, 0.904) |
| Sensitivity  (95% CI) | 0.991  (0.984, 0.998) | 0.998  (0.995, 1.001) | 0.979  (0.963, 0.995) | 0.971  (0.953, 0.989) |
| Specificity  (95% CI) | 0.638  (0.604, 0.672) | 0.682  (0.649, 0.715) | 0.238  (0.192, 0.284) | 0.220  (0.175, 0.265) |
| PPV  (95% CI) | 0.902  (0.881, 0.923) | 0.912  (0.892, 0.932) | 0.787  (0.743, 0.831) | 0.787  (0.743, 0.831) |
| NPV  (95% CI) | 0.957  (0.943, 0.971) | 0.992  (0.986, 110.403) | 0.80  (0.757, 0.843) | 0.720  (0.671, 0.769) |
| PLR  (95% CI) | 20.930  (20.913, 20.947) | 110.396  (110.389, 54.104) | 3.937  (3.866, 4.008) | 2.812  (2.714, 2.910) |
| NIR  (95% CI) | 0.048  (-0.169, 0.265) | 0.009  (-0.221, 0.239) | 0.254  (0.039, 0.469) | 0.356  (0.141, 0.571) |
| Accuracy  (95% CI) | 0.911  (0.891, 0.931) | 0.925  (0.906, 0.944) | 0.788  (0.744, 0.832) | 0.782  (0.737, 0.827) |

AUC, area under the curve; PPV, positive predictive value; NPV, negative predictive value; PLR, positive likelihood ratio; NLR, negative likelihood ratio; CI, confidence.

**Table S19.** The performance of various models for discriminating cluster size in the lineage2 cohort.

| **Parameters** | **Training set**  **(n=2099, 745 large cluster strains,**  **851 middle cluster strains,**  **503 small cluster strains)** | | **Test set**  **(n=2099, 327 large cluster strains,**  **344 middle cluster strains,**  **229 small cluster strains)** | |
| --- | --- | --- | --- | --- |
|  | **Random Forest** | **Gradient Boosting Decision Tree** | **Random Forest** | **Gradient Boosting Decision Tree** |
| Kappa | 0.551 | 0.545 | 0.467 | 0.483 |
| F1-score | 0.712 | 0.708 | 0.658 | 0.666 |
| Accuracy  (95% CI) | 0.712  (0.693, 0.731) | 0.708  (0.689, 0.727) | 0.657  (0.626, 0.688) | 0.664  (0.633, 0.695) |

**Table S20**. The performance of various models for discriminating cluster size in the lineage4 cohort.

| **Parameters** | **Training set**  **(n=2099, 745 large cluster strains,**  **851 middle cluster strains,**  **503 small cluster strains)** | | **Test set**  **(n=2099, 327 large cluster strains,**  **344 middle cluster strains,**  **229 small cluster strains)** | |
| --- | --- | --- | --- | --- |
|  | **Random Forest** | **Gradient Boosting Decision Tree** | **Random Forest** | **Gradient Boosting Decision Tree** |
| Kappa | 0.695 | 0.683 | 0.618 | 0.639 |
| F1-score | 0.827 | 0.796 | 0.754 | 0.765 |
| Accuracy  (95% CI) | 0.808  (0.794, 0.822) | 0.801  (0.787, 0.815) | 0.763  (0.741, 0.785) | 0.778  (0.756, 0.800) |

**Table S21.** The performance of various models for discriminating cross-country from within-country in the lineage2 cohort.

| **Parameters** | **Training set**  **(n=2099, 1858** **cross-country**  **strains, 241 within-country strains)** | | **Test set**  **(n=900, 89 cross-country**  **strains, 811 within-country strains)** | |
| --- | --- | --- | --- | --- |
|  | **Random Forest** | **Gradient Boosting Decision Tree** | **Random Forest** | **Gradient Boosting Decision Tree** |
| Kappa | 0.382 | 0.394 | 0.233 | 0.267 |
| AUC  (95% CI) | 0.852  (0.837, 0.867) | 0.832  (0.816, 0.848) | 0.778  (0.751, 0.805) | 0.783  (0.756, 0.810) |
| Sensitivity  (95% CI) | 0.294  (0.275, 0.313) | 0.303  (0.283, 0.323) | 0.174  (0.149, 0.199) | 0.211  (0.184, 0.238) |
| Specificity  (95% CI) | 0.987  (0.982, 0.992) | 0.988  (0.983, 0.993) | 0.986  (0.978, 0.994) | 0.981  (0.972, 0.99) |
| PPV  (95% CI) | 0.730  (0.711, 0.749) | 0.744  (0.725, 0.763) | 0.633  (0.602, 0.664) | 0.605  (0.573, 0.637) |
| NPV  (95% CI) | 0.922  (0.911, 0.933) | 0.923  (0.912, 0.934) | 0.897  (0.877, 0.917) | 0.90  (0.880, 0.920) |
| PLR  (95% CI) | 9.410  (9.397, 9.423) | 9.712  (9.699, 9.725) | 6.122  (6.101, 6.143) | 6.067  (6.047, 6.087) |
| NIR  (95% CI) | 0.106  (0.033, 0.179) | 0.103  (0.027, 0.179) | 0.163  (0.071, 0.255) | 0.165  (0.077, 0.253) |
| Accuracy  (95% CI) | 0.914  (0.902, 0.926) | 0.916  (0.904, 0.928) | 0.888  (0.867, 0.909) | 0.888  (0.867, 0.909) |

AUC, area under the curve; PPV, positive predictive value; NPV, negative predictive value; PLR, positive likelihood ratio; NLR, negative likelihood ratio; CI, confidence.

**Table S22.** The performance of various models for discriminating cross-country from within-country in the lineage4 cohort.

| **Parameters** | **Training set**  **(n=3201, 255 cross-country strains ,2946 within-country strains)** | | **Test set**  **(n=1373, 119 cross-country**  **strains,1254 within-country strains)** | |
| --- | --- | --- | --- | --- |
|  | **Random Forest** | **Gradient Boosting Decision Tree** | **Random Forest** | **Gradient Boosting Decision Tree** |
| Kappa | 0.708 | 0.605 | 0.455 | 0.456 |
| AUC  (95% CI) | 0.962  (0.955, 0.969) | 0.945  (0.937, 0.953) | 0.897  (0.881, 0.913) | 0.902  (0.886, 0.918) |
| Sensitivity  (95% CI) | 0.698  (0.682, 0.714) | 0.512  (0.495, 0.529) | 0.446  (0.420, 0.472) | 0.375  (0.349, 0.401) |
| Specificity  (95% CI) | 0.981  (0.976, 0.986) | 0.990  (0.987, 0.993) | 0.968  (0.959, 0.977) | 0.985  (0.979, 0.991) |
| PPV  (95% CI) | 0.766  (0.751, 0.781) | 0.818  (0.805, 0.813) | 0.556  (0.530, 0.582) | 0.703  (0.679, 0.727) |
| NPV  (95% CI) | 0.973  (0.967, 0.979) | 0.959  (0.952, 0.966) | 0.952  (0.941, 0.963) | 0.943  (0.931, 0.955) |
| PLR  (95% CI) | 28.709  (28.704,28.714) | 20.058  (20.051, 20.065) | 11.496  (11.486, 11.506) | 12.272  (12.260, 12.284) |
| NIR  (95% CI) | 0.035  (-0.029, 0.099) | 0.050  (-0.025, 0.125) | 0.087  (0.022, 0.152) | 0.081  (-0.004, 0.166) |
| Accuracy  (95% CI) | 0.958  (0.951, 0.965) | 0.952  (0.945, 0.959) | 0.926  (0.912, 0.940) | 0.932  (0.919, 0.945) |

AUC, area under the curve; PPV, positive predictive value; NPV, negative predictive value; PLR, positive likelihood ratio; NLR, negative likelihood ratio; CI, confidence.

**Table S23.** The performance of various models for discriminating cross-regional from within-regional in the lineage2 cohort.

| **Parameters** | **Training set**  **(n=2099, 231 cross-regional**  **strains ,1868 within-regional strains)** | | **Test set**  **(n=900, 90 cross-regional**  **strains,810 within-regional strains)** | |
| --- | --- | --- | --- | --- |
|  | **Random Forest** | **Gradient Boosting Decision Tree** | **Random Forest** | **Gradient Boosting Decision Tree** |
| Kappa | 0.396 | 0.389 | 0.279 | 0.265 |
| AUC  (95% CI) | 0.858  (0.843, 0.873) | 0.860  (0.845, 0.875) | 0.797  (0.771, 0.823) | 0.803  (0.777, 0.829) |
| Sensitivity  (95% CI) | 0.313  (0.293, 0.333) | 0.305  (0.285, 0.325) | 0.213  (0.186, 0.240) | 0.204  (0.178, 0.230) |
| Specificity  (95% CI) | 0.985  (0.980, 0.990) | 0.986  (0.981, 0.991) | 0.985  (0.977, 0.993) | 0.984  (0.976, 0.992) |
| PPV  (95% CI) | 0.717  (0.698, 0.736) | 0.716  (0.697, 0.735) | 0.625  (0.593, 0.657) | 0606  (0.574, 0.638) |
| NPV  (95% CI) | 0.922  (0.911, 0.933) | 0.923  (0.912, 0.934) | 0.915  (0.897, 0.933) | 0.910  (0.891, 0.929) |
| PLR  (95% CI) | 9.195  (9.183, 9.207) | 9.254  (9.242, 9.266) | 7.331  (7.313, 7.349) | 6.737  (6.718, 6.756) |
| NIR  (95% CI) | 0.109  (0.038, 0.180) | 0.108  (0.037, 0.179) | 0.136  (0.045, 0.227) | 0.148  (0.060, 0.236) |
| Accuracy  (95% CI) | 0.912  (0.90, 0.924) | 0.913  (0.901, 0.925) | 0.904  (0.885, 0.923) | 0.899  (0.879, 0.919) |

AUC, area under the curve; PPV, positive predictive value; NPV, negative predictive value; PLR, positive likelihood ratio; NLR, negative likelihood ratio; CI, confidence.

**Table S24.** Generalized linear mixed model analysis on clustered and non-clustered strains in the lineage1 cohort.

| **Gene** | **Position** | **SNP** | **Amino acid changes** | ***P* value** | **OR (95%CI)** |
| --- | --- | --- | --- | --- | --- |
| Rv3245c | 3625783 | C831T | Asp277Asp | <0.001 | 1.669 (1.486, 1.874) |
| Rv3245c | 3626158 | C456T | Asn152Asn | 0.157 | 2.226 (1.265, 3.916) |
| Rv0014c | 15647 | G1824A | Pro608Pro | 0.01 | 1.428 (1.244, 1.639) |
| Rv3246c | 3626758 | G592A | Val198Ile | 0.026 | 1.976 (1.455, 2.683) |
| Rv2027c | 2273756 | G753C | Leu251Phe | 0.083 | 0.829 (0.744, 0.923) |
| Rv0195 | 231268 | C370T | Pro124Ser | 0.004 | 2.697 (1.908, 3.811) |
| Rv1675c | 1900307 | G669A | Leu223Leu | 0.025 | 1.976 (1.456, 2.68) |
| Rv0844c | 940511 | T596G | Val199Gly | 0.025 | 1.976 (1.458, 2.678) |
| Rv1266c | 1414095 | T1746G | Ala582Ala | 0.026 | 1.976 (1.456, 2.68) |
| Rv1747 | 1974609 | C980T | Thr327Ile | 0.006 | 2.344 (1.723, 3.19) |
| Rv1813c | 2055888 | G225A | Gln75Gln | 0.95 | 1.005 (0.932, 1.083) |
| Rv1028c | 1151267 | C420T | Ile140Ile | 0.08 | 0.734 (0.615, 0.876) |
| Rv3132c | 3498418 | T848C | Ile283Thr | N/A | N/A |
| Rv1813c | 2055919 | C194G | Pro65Arg | 0.021 | 2.038 (1.496, 2.776) |
| Rv1690 | 1915717 | C191T | Thr64Ile | 0.031 | 1.968 (1.438, 2.694) |
| Rv1266c | 1414564 | T1277A | Ile426Asn | N/A | N/A |
| Rv3245c | 3626314 | G300T | Leu100Leu | 0.009 | 1.669 (1.37, 2.032) |
| Rv0982 | 1098523 | T1016A | Leu339His | 0.42 | 1.948 (0.853, 4.45) |
| Rv0758 | 852641 | C246T | Tyr82Tyr | 0.005 | 0.731 (0.654, 0.815) |
| Rv1028c | 1150250 | C1437T | Thr479Thr | 0.111 | 0.513 (0.338, 0.78) |
| Rv0014c | 16378 | G1093A | Val365Ile | 0.016 | 2.094 (1.542, 2.843) |
| Rv0195 | 231021 | C123A | Cys41* | 0.557 | 0.724 (0.418, 1.255) |
| Rv3220c | 3597204 | G331A | Val111Ile | 0.087 | 2.094 (1.359, 3.225) |
| Rv1675c | 1900349 | A627G | Pro209Pro | 0.026 | 1.976 (1.456, 2.68) |
| Rv1690 | 1915796 | G270A | Met90Ile | 0.006 | 2.344 (1.721, 3.193) |
| Rv3133c | 3499640 | T276C | Asp92Asp | 0.061 | 1.533 (1.221, 1.923) |
| Rv0490 | 580246 | C898T | Arg300Cys | 0.133 | 0.873 (0.797, 0.956) |
| Rv0014c | 16070 | C1401G | Val467Val | 0.025 | 1.976 (1.458, 2.678) |
| Rv0490 | 579424 | A76C | Met26Leu | 0.114 | 1.976 (1.284, 3.04) |
| Rv0758 | 853595 | C1200T | Ala400Ala | 0.113 | 1.976 (1.285, 3.037) |
| Rv1028c | 1149557 | C2130T | Tyr710Tyr | 0.013 | 1.618 (1.334, 1.962) |
| Rv1033c | 1158406 | G331A | Ala111Thr | 0.113 | 1.976 (1.285, 3.037) |
| Rv2027c | 2272809 | G1700A | Arg567Gln | 0.114 | 1.976 (1.284, 3.04) |
| Rv2027c | 2272859 | G1650A | Ala550Ala | 0.025 | 1.976 (1.457, 2.681) |
| Rv2027c | 2273285 | T1224C | Gly408Gly | 0.114 | 1.976 (1.284, 3.04) |
| Rv2027c | 2274359 | C150T | Ile50Ile | 0.114 | 1.975 (1.284, 3.04) |
| Rv3764c | 4209656 | T1354C | Ser452Pro | 0.025 | 1.975 (1.456, 2.68) |
| Rv1032c | 1157601 | T355G | Ser119Ala | 0.114 | 1.975 (1.284, 3.04) |
| Rv2027c | 2274254 | G255C | Gly85Gly | 0.022 | 2.038 (1.495, 2.779) |
| Rv0079 | 88574 | G371A | Arg124His | 0.008 | 2.054 (1.568, 2.691) |
| Rv0601c | 698826 | G169A | Val57Met | 0.114 | 1.976 (1.284, 3.04) |
| Rv1368 | 1541633 | G614A | Gly205Asp | 0.025 | 1.976 (1.456, 2.68) |
| Rv1813c | 2055964 | T149G | Leu50Trp | 0.113 | 1.976 (1.285, 3.037) |
| Rv0845 | 941743 | T554A | Phe185Tyr | <0.001 | 1.669 (1.499, 1.857) |
| Rv1675c | 1900603 | G373T | Ala125Ser | 0.011 | 1.461 (1.259, 1.696) |
| Rv1266c | 1414653 | G1188A | Pro396Pro | N/A | N/A |
| Rv0014c | 15935 | C1536G | Leu512Leu | 0.003 | 2.484 (1.831, 3.37) |
| Rv3220c | 3597307 | C228T | Ala76Ala | 0.006 | 2.344 (1.721, 3.193) |
| Rv1028c | 1150104 | C1583T | Ala528Val | 0.002 | 1.976 (1.589, 2.457) |
| Rv1057 | 1179572 | C177T | Asp59Asp | 0.046 | 1.539 (1.24, 1.91) |
| Rv1266c | 1415097 | G744C | Ala248Ala | 0.218 | 0.944 (0.9, 0.989) |
| Rv3246c | 3627311 | C39T | Asp13Asp | 0.007 | 1.968 (1.528, 2.535) |
| Rv3764c | 4209633 | G1377C | Ala459Ala | 0.021 | 2.038 (1.496, 2.776) |
| Rv1033c | 1158079 | C658A | Arg220Ser | 0.096 | 2.038 (1.328, 3.127) |
| Rv1221 | 1364766 | C354T | Ile118Ile | 0.059 | 0.733 (0.623, 0.864) |
| Rv1009 | 1128665 | T575C | Val192Ala | 0.008 | 1.327 (1.192, 1.477) |
| Rv3764c | 4210626 | G384A | Pro128Pro | 0.491 | 1.234 (0.909, 1.674) |
| Rv1028c | 1150591 | C1096T | Gln366* | 0.047 | 1.539 (1.24, 1.911) |
| Rv1028c | 1150283 | C1404T | Thr468Thr | 0.152 | 1.416 (1.111, 1.806) |
| Rv0844c | 940966 | G141A | Leu47Leu | 0.54 | 0.914 (0.789, 1.059) |

OR, odds ratio; CI, confidence interval.

**Table S25.** Generalized linear mixed model analysis on clustered and non-clustered strains in the lineage2 cohort.

| **Gene** | **Position** | **SNP** | **Amino acid changes** | ***P* value** | **OR (95%CI)** |
| --- | --- | --- | --- | --- | --- |
| Rv1221 | 1364706 | G294A | Leu98Leu | <0.001 | 1.111 (1.093, 1.129) |
| Rv0930 | 1037012 | T14C | Met5Thr | <0.001 | 1.452 (1.328, 1.587) |
| Rv0845 | 941722 | T533G | Ile178Ser | <0.001 | 1.177 (1.157, 1.197) |
| Rv0982 | 1098417 | G910A | Asp304Asn | <0.001 | 1.531 (1.443, 1.624) |
| Rv0844c | 940748 | A359C | Lys120Thr | <0.001 | 1.289 (1.25, 1.33) |
| Rv1368 | 1541798 | C779T | Ala260Val | <0.001 | 0.869 (0.841, 0.899) |
| Rv0902c | 1005093 | G749A | Arg250His | <0.001 | 1.857 (1.713, 2.014) |
| Rv2247 | 2521309 | G567A | Gly189Gly | <0.001 | 1.914 (1.744, 2.1) |
| Rv0758 | 852953 | G558T | Pro186Pro | <0.001 | 1.614 (1.511, 1.725) |
| Rv1027c | 1148930 | G178A | Gly60Ser | 0.002 | 1.076 (1.051, 1.101) |
| Rv1027c | 1148482 | C626A | Ser209* | 0.01 | 1.194 (1.114, 1.279) |
| Rv2027c | 2273888 | A621G | Ser207Ser | <0.001 | 1.335 (1.251, 1.425) |
| Rv0601c | 698968 | C27T | Gly9Gly | 0.106 | 1.045 (1.017, 1.074) |
| Rv2247 | 2521342 | T600C | Asp200Asp | 0.026 | 1.24 (1.126, 1.365) |
| Rv3132c | 3498851 | C415G | His139Asp | <0.001 | 1.445 (1.315, 1.587) |
| Rv1743 | 1970117 | C1114T | Pro372Ser | 0.001 | 1.759 (1.483, 2.088) |
| Rv1743 | 1969275 | T272C | Val91Ala | <0.001 | 1.209 (1.148, 1.274) |
| Rv2247 | 2521428 | A686G | Asp229Gly | 0.691 | 1.038 (0.945, 1.14) |
| Rv1028c | 1149234 | G2453A | Gly818Asp | <0.001 | 1.818 (1.533, 2.158) |
| Rv0930 | 1037664 | G666A | Leu222Leu | <0.001 | 1.824 (1.537, 2.164) |
| Rv1057 | 1180563 | C1168T | Pro390Ser | 0.002 | 1.791 (1.49, 2.153) |
| Rv0602c | 699274 | G526A | Glu176Lys | 0.001 | 1.747 (1.473, 2.073) |
| Rv0602c | 699386 | A414G | Ser138Ser | 0.003 | 1.737 (1.445, 2.088) |
| Rv2027c | 2273635 | T874C | Ser292Pro | 0.004 | 1.701 (1.415, 2.044) |
| Rv1028c | 1150585 | C1102T | Pro368Ser | <0.001 | 0.591 (0.55, 0.634) |
| Rv3245c | 3625065 | A1549C | Met517Leu | 0.221 | 1.122 (1.021, 1.232) |
| Rv0079 | 88852 | A649G | Thr217Ala | 0.007 | 1.837 (1.467, 2.3) |
| Rv3245c | 3625643 | A971G | His324Arg | <0.001 | 1.702 (1.502, 1.929) |
| Rv2027c | 2273930 | G579C | Ala193Ala | 0.028 | 0.566 (0.436, 0.733) |
| Rv0844c | 940570 | A537G | Leu179Leu | 0.015 | 0.717 (0.626, 0.823) |
| Rv1743 | 1969405 | G402A | Gly134Gly | 0.019 | 1.545 (1.283, 1.861) |
| Rv0079 | 88700 | C497T | Thr166Ile | <0.001 | 1.169 (1.133, 1.206) |
| Rv2984 | 3341602 | A1749C | Ala583Ala | 0.354 | 1.15 (0.989, 1.338) |
| Rv3220c | 3597249 | G286C | Gly96Arg | 0.3 | 0.919 (0.846, 0.997) |
| Rv3245c | 3625504 | G1110A | Ala370Ala | 0.04 | 1.59 (1.269, 1.994) |
| Rv1747 | 1974325 | C696A | Ile232Ile | 0.003 | 1.711 (1.423, 2.056) |
| Rv1675c | 1900781 | G195A | Gly65Gly | <0.001 | 1.383 (1.269, 1.507) |
| Rv1368 | 1541442 | C423T | Val141Val | 0.003 | 0.466 (0.36, 0.605) |
| Rv0602c | 699610 | G190A | Gly64Ser | 0.162 | 1.269 (1.07, 1.504) |
| Rv1747 | 1974731 | T1102C | Tyr368His | 0.039 | 1.709 (1.318, 2.217) |
| Rv1032c | 1157092 | G864A | Glu288Glu | 0.045 | 0.406 (0.259, 0.636) |
| Rv2247 | 2520964 | G222A | Thr74Thr | 0.078 | 0.45 (0.287, 0.708) |
| Rv2984 | 3340293 | T440C | Val147Ala | 0.031 | 1.606 (1.29, 1.999) |
| Rv1009 | 1128466 | G376A | Val126Ile | 0.086 | 0.463 (0.295, 0.725) |
| Rv1266c | 1414295 | C1546T | His516Tyr | 0.079 | 0.454 (0.29, 0.712) |
| Rv2247 | 2520772 | C30T | Gly10Gly | 0.025 | 0.636 (0.52, 0.778) |
| Rv0844c | 940596 | A511C | Ser171Arg | 0.088 | 0.582 (0.423, 0.799) |
| Rv0845 | 941475 | T286G | Phe96Val | 0.833 | 0.982 (0.904, 1.067) |
| Rv1028c | 1149956 | A1731G | Glu577Glu | 0.074 | 0.567 (0.412, 0.779) |
| Rv3132c | 3497569 | G1697A | Ser566Asn | 0.067 | 0.558 (0.406, 0.767) |
| Rv3764c | 4210726 | C284A | Thr95Lys | 0.035 | 1.956 (1.422, 2.691) |
| Rv1266c | 1415295 | G546A | Met182Ile | 0.069 | 0.622 (0.479, 0.807) |
| Rv1743 | 1969837 | G834A | Ala278Ala | <0.001 | 1.528 (1.354, 1.725) |
| Rv1743 | 1970565 | T1562G | Leu521Arg | 0.024 | 1.383 (1.197, 1.597) |
| Rv0758 | 853215 | C820G | Leu274Val | 0.008 | 1.817 (1.451, 2.275) |
| Rv0758 | 852994 | G599T | Gly200Val | 0.048 | 0.409 (0.261, 0.643) |
| Rv3765c | 4211492 | G293C | Gly98Ala | <0.001 | 1.582 (1.388, 1.804) |
| Rv2027c | 2274003 | A506C | Tyr169Ser | <0.001 | 1.861 (1.57, 2.205) |
| Rv3245c | 3626562 | C52T | Pro18Ser | 0.317 | 0.965 (0.931, 1) |
| Rv3764c | 4210274 | T736C | Cys246Arg | 0.01 | 0.807 (0.743, 0.877) |

OR, odds ratio; CI, confidence interval.

**Table S26.** Generalized linear mixed model analysis on clustered and non-clustered strains in the lineage3 cohort.

| **Gene** | **Position** | **SNP** | **Amino acid changes** | ***P* value** | **OR (95%CI)** |
| --- | --- | --- | --- | --- | --- |
| Rv1032c | 1157010 | T946C | Tyr316His | <0.001 | 1.428 (1.369, 1.489) |
| Rv3849 | 4323831 | C333T | Ser111Ser | <0.001 | 1.906 (1.756, 2.069) |
| Rv3133c | 3499497 | C419G | Ala140Gly | 0.191 | 0.909 (0.845, 0.978) |
| Rv3132c | 3497580 | A1686G | Val562Val | 0.018 | 1.151 (1.084, 1.223) |
| Rv0491 | 581170 | A362C | Asp121Ala | <0.001 | 1.817 (1.575, 2.096) |
| Rv0195 | 231127 | G229T | Val77Phe | 0.003 | 1.309 (1.195, 1.433) |
| Rv1266c | 1415114 | C727T | Pro243Ser | 0.02 | 2.01 (1.489, 2.713) |
| Rv1057 | 1179581 | G186A | Ser62Ser | 0.022 | 1.986 (1.47, 2.683) |
| Rv1747 | 1973740 | G111C | Ala37Ala | 0.016 | 2.044 (1.519, 2.751) |
| Rv3245c | 3625065 | A1549C | Met517Leu | 0.085 | 1.339 (1.131, 1.586) |
| Rv0845 | 941610 | C421T | Pro141Ser | 0.033 | 0.404 (0.264, 0.619) |
| Rv0014c | 15917 | C1554T | Thr518Thr | 0.015 | 1.441 (1.24, 1.674) |
| Rv0982 | 1098984 | G1477A | Val493Ile | <0.001 | 1.236 (1.164, 1.313) |
| Rv3245c | 3626590 | C24T | Arg8Arg | 0.006 | 2.273 (1.687, 3.062) |
| Rv0930 | 1037012 | T14C | Met5Thr | 0.098 | 1.287 (1.105, 1.498) |
| Rv2984 | 3340015 | G162A | Leu54Leu | 0.007 | 2.221 (1.652, 2.986) |
| Rv1747 | 1975817 | G2188A | Val730Ile | 0.002 | 1.954 (1.568, 2.435) |
| Rv0930 | 1037643 | C645G | Gly215Gly | 0.006 | 1.702 (1.402, 2.067) |
| Rv0195 | 231446 | A548C | Lys183Thr | <0.001 | 2.065 (1.689, 2.524) |
| Rv1027c | 1149046 | T62C | Leu21Pro | 0.076 | 0.475 (0.312, 0.723) |
| Rv1675c | 1900401 | G575T | Arg192Leu | 0.005 | 2.298 (1.706, 3.096) |
| Rv1032c | 1157761 | G195A | Ala65Ala | 0.044 | 0.425 (0.277, 0.651) |
| Rv2710 | 3023168 | C708T | Asp236Asp | 0.976 | 1.003 (0.903, 1.114) |
| Rv1747 | 1974100 | C471G | Ala157Ala | 0.783 | 0.987 (0.94, 1.037) |
| Rv1747 | 1974089 | C460T | Arg154Trp | 0.009 | 2.179 (1.618, 2.936) |
| Rv0758 | 852843 | G448T | Val150Phe | 0.008 | 2.184 (1.624, 2.936) |
| Rv0758 | 852988 | G593A | Gly198Asp | 0.063 | 2.173 (1.43, 3.3) |
| Rv0758 | 853452 | A1057G | Thr353Ala | 0.09 | 2.03 (1.336, 3.083) |
| Rv1027c | 1149063 | C45T | Arg15Arg | 0.001 | 2.199 (1.726, 2.801) |
| Rv3764c | 4210732 | C278T | Ala93Val | 0.012 | 2.106 (1.567, 2.832) |
| Rv1368 | 1541799 | G780A | Ala260Ala | 0.025 | 1.956 (1.452, 2.635) |
| Rv2247 | 2521342 | T600C | Asp200Asp | 0.466 | 1.141 (0.952, 1.368) |
| Rv3765c | 4211370 | G415A | Val139Met | <0.001 | 2.171 (1.799, 2.62) |
| Rv1028c | 1149307 | G2380T | Gly794Trp | 0.009 | 0.528 (0.414, 0.674) |
| Rv0195 | 231517 | C619G | Arg207Gly | 0.026 | 1.958 (1.446, 2.651) |
| Rv0758 | 853285 | T890C | Met297Thr | 0.108 | 1.96 (1.289, 2.98) |
| Rv3246c | 3627101 | C249T | Thr83Thr | 0.054 | 2.237 (1.473, 3.397) |
| Rv0758 | 853089 | G694T | Ala232Ser | 0.049 | 2.275 (1.498, 3.456) |
| Rv0844c | 940680 | G427A | Glu143Lys | 0.009 | 2.168 (1.613, 2.915) |
| Rv0930 | 1037354 | C356A | Thr119Asn | 0.006 | 1.605 (1.35, 1.908) |
| Rv0844c | 941093 | A14G | Gln5Arg | 0.06 | 0.799 (0.71, 0.9) |
| Rv2247 | 2522039 | G1297T | Asp433Tyr | 0.066 | 1.473 (1.192, 1.818) |
| Rv3246c | 3626789 | C561T | His187His | 0.006 | 2.275 (1.69, 3.062) |
| Rv0758 | 852775 | T380C | Leu127Ser | 0.117 | 1.234 (1.079, 1.411) |
| Rv1266c | 1415630 | T211C | Leu71Leu | 0.206 | 0.805 (0.678, 0.956) |
| Rv1266c | 1414201 | G1640C | Gly547Ala | 0.015 | 1.921 (1.471, 2.509) |
| Rv0758 | 852728 | C333T | Val111Val | 0.142 | 0.533 (0.347, 0.818) |
| Rv1032c | 1157496 | G460T | Ala154Ser | 0.245 | 1.215 (1.027, 1.438) |
| Rv1266c | 1414045 | T1796C | Val599Ala | 0.005 | 2.305 (1.711, 3.105) |
| Rv1028c | 1149551 | G2136A | Glu712Glu | 0.551 | 0.85 (0.646, 1.117) |
| Rv0758 | 853461 | G1066T | Val356Phe | 0.137 | 0.537 (0.353, 0.815) |
| Rv0195 | 231114 | C216G | Ala72Ala | 0.773 | 1.057 (0.872, 1.28) |
| Rv0014c | 17307 | G164A | Arg55His | 0.146 | 0.544 (0.358, 0.827) |
| Rv0081 | 89749 | C175A | Arg59Ser | 0.132 | 0.533 (0.351, 0.809) |
| Rv0195 | 231352 | G454T | Ala152Ser | 0.104 | 0.507 (0.334, 0.77) |
| Rv1028c | 1150199 | C1488A | Gly496Gly | 0.148 | 0.546 (0.36, 0.829) |
| Rv1266c | 1415550 | G291A | Glu97Glu | 0.131 | 0.532 (0.35, 0.807) |
| Rv1368 | 1541150 | C131T | Thr44Ile | 0.191 | 0.578 (0.38, 0.879) |
| Rv1266c | 1414719 | G1122A | Gln374Gln | 0.09 | 2.05 (1.343, 3.13) |
| Rv1028c | 1150585 | C1102T | Pro368Ser | 0.692 | 1.081 (0.888, 1.317) |

OR, odds ratio; CI, confidence interval.

**Table S27.** Generalized linear mixed model analysis on clustered and non-clustered strains in the lineage4 cohort.

| **Gene** | **Position** | **SNP** | **Amino acid changes** | ***P* value** | **OR (95%CI)** |
| --- | --- | --- | --- | --- | --- |
| Rv2247 | 2520761 | G19A | Glu7Lys | <0.001 | 0.723 (0.703, 0.744) |
| Rv3764c | 4209859 | G1151T | Arg384Leu | <0.001 | 1.422 (1.391, 1.454) |
| Rv1028c | 1149433 | T2254G | Leu752Val | <0.001 | 0.76 (0.731, 0.79) |
| Rv2710 | 3023036 | G576A | Glu192Glu | <0.001 | 1.634 (1.581, 1.689) |
| Rv0930 | 1037355 | T357C | Thr119Thr | <0.001 | 1.099 (1.081, 1.116) |
| Rv2247 | 2521699 | G957A | Val319Val | <0.001 | 1.56 (1.492, 1.632) |
| Rv0758 | 852577 | C182A | Thr61Lys | <0.001 | 1.527 (1.459, 1.597) |
| Rv0600c | 698144 | C267T | Asp89Asp | <0.001 | 2.858 (2.363, 3.456) |
| Rv1368 | 1541356 | A337G | Arg113Gly | <0.001 | 1.584 (1.495, 1.679) |
| Rv1028c | 1150585 | C1102T | Pro368Ser | 0.274 | 1.107 (1.009, 1.215) |
| Rv1033c | 1158549 | A188G | Asp63Gly | <0.001 | 1.582 (1.511, 1.657) |
| Rv0602c | 699466 | G334A | Gly112Ser | <0.001 | 1.335 (1.264, 1.411) |
| Rv2984 | 3340328 | G475T | Val159Phe | <0.001 | 0.928 (0.909, 0.946) |
| Rv0014c | 16055 | G1416T | Pro472Pro | <0.001 | 1.28 (1.24, 1.322) |
| Rv1747 | 1973649 | C20T | Ala7Val | <0.001 | 1.383 (1.311, 1.458) |
| Rv0930 | 1037911 | C913T | Arg305* | 0.015 | 1.551 (1.296, 1.857) |
| Rv2247 | 2521342 | T600C | Asp200Asp | 0.187 | 1.14 (1.033, 1.259) |
| Rv1032c | 1156979 | G977T | Arg326Leu | <0.001 | 1.264 (1.189, 1.343) |
| Rv1028c | 1151473 | A214G | Thr72Ala | <0.001 | 1.429 (1.334, 1.531) |
| Rv0981 | 1097206 | G385A | Ala129Thr | 0.004 | 0.588 (0.49, 0.706) |
| Rv1009 | 1128567 | C477A | Val159Val | <0.001 | 2.121 (1.723, 2.612) |
| Rv0758 | 853579 | C1184G | Ala395Gly | <0.001 | 1.172 (1.124, 1.223) |
| Rv0490 | 579456 | C108T | Arg36Arg | 0.002 | 1.285 (1.183, 1.397) |
| Rv0601c | 698968 | C27T | Gly9Gly | 0.036 | 0.82 (0.746, 0.902) |
| Rv0014c | 16439 | C1032T | Val344Val | 0.001 | 0.54 (0.45, 0.649) |
| Rv1057 | 1180217 | C822T | Ile274Ile | 0.005 | 0.561 (0.457, 0.689) |
| Rv2247 | 2520833 | G91A | Val31Met | <0.001 | 0.535 (0.458, 0.624) |
| Rv1266c | 1414040 | G1801A | Val601Ile | <0.001 | 0.519 (0.444, 0.606) |
| Rv1057 | 1179980 | C585A | Gly195Gly | <0.001 | 1.426 (1.342, 1.516) |
| Rv0014c | 16226 | G1245A | Glu415Glu | 0.04 | 1.33 (1.157, 1.528) |
| Rv2984 | 3340300 | C447T | Pro149Pro | <0.001 | 1.54 (1.419, 1.672) |
| Rv0903c | 1006538 | A25C | Thr9Pro | 0.001 | 0.543 (0.452, 0.652) |
| Rv0081 | 89674 | C100T | Arg34Cys | 0.006 | 0.525 (0.414, 0.664) |
| Rv0758 | 853057 | C662T | Ala221Val | <0.001 | 1.56 (1.432, 1.701) |
| Rv1028c | 1150779 | A908C | Gln303Pro | 0.012 | 0.551 (0.435, 0.698) |
| Rv1028c | 1151398 | C289A | Leu97Ile | <0.001 | 1.293 (1.203, 1.39) |
| Rv1266c | 1414551 | G1290A | Pro430Pro | 0.008 | 0.462 (0.345, 0.618) |
| Rv3765c | 4211659 | G126T | Ser42Ser | 0.016 | 0.566 (0.447, 0.716) |
| Rv1028c | 1149367 | C2320T | Arg774Trp | 0.032 | 1.554 (1.265, 1.91) |
| Rv1028c | 1150971 | A716C | Glu239Ala | 0.003 | 0.582 (0.485, 0.699) |
| Rv3220c | 3596416 | G1119C | Val373Val | 0.001 | 1.23 (1.156, 1.309) |
| Rv0758 | 853153 | T758G | Phe253Cys | 0.001 | 1.358 (1.244, 1.483) |
| Rv1266c | 1414640 | C1201T | Pro401Ser | 0.008 | 0.58 (0.473, 0.712) |
| Rv3220c | 3596506 | C1029T | Ala343Ala | 0.003 | 1.318 (1.202, 1.445) |
| Rv0014c | 16652 | G819C | Leu273Leu | 0.008 | 0.465 (0.348, 0.621) |
| Rv1266c | 1414609 | T1232C | Val411Ala | 0.004 | 0.635 (0.543, 0.743) |
| Rv1743 | 1969269 | T266G | Leu89Arg | 0.022 | 0.732 (0.638, 0.839) |
| Rv3246c | 3626927 | G423A | Pro141Pro | 0.008 | 0.466 (0.349, 0.623) |
| Rv1221 | 1364592 | G180A | Val60Val | 0.546 | 1.132 (0.922, 1.39) |
| Rv1813c | 2055762 | C351T | Thr117Thr | 0.008 | 1.446 (1.26, 1.66) |
| Rv3765c | 4211740 | C45G | Asp15Glu | 0.005 | 1.948 (1.536, 2.472) |
| Rv0845 | 941601 | A412C | Ile138Leu | <0.001 | 0.656 (0.582, 0.739) |
| Rv3132c | 3499187 | C79T | Leu27Leu | 0.001 | 1.481 (1.309, 1.677) |
| Rv0014c | 15647 | G1824A | Pro608Pro | 0.016 | 0.566 (0.447, 0.716) |
| Rv0014c | 15886 | C1585A | Arg529Ser | 0.002 | 0.534 (0.435, 0.656) |
| Rv1033c | 1158283 | C454T | His152Tyr | 0.049 | 0.566 (0.424, 0.755) |
| Rv1266c | 1415029 | C812T | Ala271Val | 0.048 | 0.565 (0.423, 0.754) |
| Rv2984 | 3341404 | C1551T | Asn517Asn | 0.276 | 1.114 (1.009, 1.23) |
| Rv3245c | 3626581 | T33C | Gly11Gly | 0.002 | 1.605 (1.373, 1.876) |
| Rv3764c | 4210164 | C846T | Val282Val | 0.065 | 0.638 (0.5, 0.815) |

OR, odds ratio; CI, confidence interval.

**Table S28.** Generalized linear mixed model analysis on clustered and non-clustered strains in the lineage2.2.1 cohort.

| **Gene** | **Position** | **SNP** | **Amino acid changes** | ***P* value** | **OR(95%CI)** |
| --- | --- | --- | --- | --- | --- |
| Rv1221 | 1364706 | G294A | Leu98Leu | <0.001 | 1.11(1.092,1.127) |
| Rv0845 | 941722 | T533G | Ile178Ser | <0.001 | 1.185(1.165,1.206) |
| Rv0844c | 940748 | A359C | Lys120Thr | <0.001 | 1.294(1.255,1.335) |
| Rv0079 | 88700 | C497T | Thr166Ile | <0.001 | 1.172(1.137,1.209) |
| Rv0902c | 1005093 | G749A | Arg250His | <0.001 | 1.879(1.733,2.038) |
| Rv1368 | 1541798 | C779T | Ala260Val | 0.908 | 0.994(0.946,1.045) |
| Rv1027c | 1148930 | G178A | Gly60Ser | 0.036 | 1.055(1.028,1.083) |
| Rv0758 | 852953 | G558T | Pro186Pro | <0.001 | 1.631(1.527,1.742) |
| Rv2247 | 2521309 | G567A | Gly189Gly | <0.001 | 1.9(1.73,2.088) |
| Rv1027c | 1148482 | C626A | Ser209* | <0.001 | 1.811(1.632,2.01) |
| Rv0601c | 698968 | C27T | Gly9Gly | 0.385 | 1.023(0.996,1.051) |
| Rv2247 | 2521342 | T600C | Asp200Asp | 0.328 | 1.093(0.998,1.197) |
| Rv0930 | 1037012 | T14C | Met5Thr | 0.006 | 1.221(1.135,1.314) |
| Rv2247 | 2521428 | A686G | Asp229Gly | 0.003 | 0.764(0.698,0.837) |
| Rv3132c | 3498851 | C415G | His139Asp | <0.001 | 1.456(1.324,1.602) |
| Rv0930 | 1037664 | G666A | Leu222Leu | 0.001 | 1.804(1.517,2.145) |
| Rv2027c | 2273888 | A621G | Ser207Ser | <0.001 | 1.344(1.259,1.436) |
| Rv1743 | 1969275 | T272C | Val91Ala | <0.001 | 1.225(1.163,1.29) |
| Rv0602c | 699386 | A414G | Ser138Ser | 0.002 | 1.752(1.456,2.109) |
| Rv1028c | 1149234 | G2453A | Gly818Asp | 0.001 | 1.799(1.513,2.138) |
| Rv2984 | 3341602 | A1749C | Ala583Ala | 0.325 | 1.162(0.998,1.353) |
| Rv3765c | 4211492 | G293C | Gly98Ala | <0.001 | 1.592(1.395,1.817) |
| Rv3764c | 4210296 | G714A | Ser238Ser | 0.002 | 0.559(0.464,0.672) |
| Rv1743 | 1969495 | C492T | Thr164Thr | <0.001 | 0.593(0.518,0.68) |
| Rv3764c | 4210838 | A172G | Arg58Gly | 0.213 | 1.07(1.014,1.13) |
| Rv1743 | 1969837 | G834A | Ala278Ala | <0.001 | 1.568(1.388,1.772) |
| Rv1028c | 1149316 | G2371A | Glu791Lys | 0.131 | 1.095(1.031,1.163) |
| Rv3132c | 3497859 | C1407T | Asp469Asp | 0.153 | 1.14(1.04,1.25) |
| Rv1743 | 1970117 | C1114T | Pro372Ser | 0.001 | 1.737(1.461,2.065) |
| Rv1057 | 1180563 | C1168T | Pro390Ser | 0.001 | 1.809(1.504,2.177) |
| Rv1028c | 1151648 | G39A | Thr13Thr | 0.015 | 0.459(0.333,0.633) |
| Rv1368 | 1541442 | C423T | Val141Val | 0.004 | 0.468(0.36,0.608) |
| Rv3764c | 4210321 | T689G | Leu230Arg | 0.012 | 1.765(1.408,2.212) |
| Rv3245c | 3625643 | A971G | His324Arg | <0.001 | 1.718(1.514,1.948) |
| Rv2984 | 3340293 | T440C | Val147Ala | 0.031 | 1.611(1.293,2.008) |
| Rv0601c | 698737 | C258A | Pro86Pro | 0.096 | 1.162(1.062,1.271) |
| Rv0845 | 942196 | G1007T | Gly336Val | 0.056 | 0.542(0.394,0.747) |
| Rv0081 | 89670 | G96C | Arg32Arg | 0.013 | 1.212(1.122,1.309) |
| Rv0844c | 940570 | A537G | Leu179Leu | 0.017 | 0.72(0.627,0.826) |
| Rv1028c | 1149956 | A1731G | Glu577Glu | 0.079 | 0.569(0.414,0.784) |
| Rv0757 | 852013 | C406G | Leu136Val | 0.116 | 1.288(1.096,1.513) |
| Rv1266c | 1415026 | C815T | Ala272Val | 0.049 | 0.533(0.387,0.734) |
| Rv0014c | 17322 | C149A | Pro50His | 0.093 | 0.584(0.424,0.804) |
| Rv0758 | 852946 | T551G | Leu184Arg | 0.094 | 0.469(0.298,0.737) |
| Rv1032c | 1157092 | G864A | Glu288Glu | 0.047 | 0.407(0.258,0.64) |
| Rv0758 | 853200 | C805T | Arg269Cys | 0.04 | 0.519(0.377,0.715) |
| Rv1747 | 1974043 | C414A | Gly138Gly | 0.094 | 0.469(0.298,0.737) |
| Rv0758 | 853020 | T625G | Trp209Gly | 0.092 | 0.583(0.423,0.803) |
| Rv3765c | 4211221 | C564T | Arg188Arg | <0.001 | 1.397(1.281,1.522) |
| Rv3220c | 3596908 | G627T | Glu209Asp | 0.086 | 0.459(0.292,0.723) |
| Rv0602c | 699274 | G526A | Glu176Lys | 0.002 | 1.725(1.451,2.05) |
| Rv3245c | 3625065 | A1549C | Met517Leu | <0.001 | 0.783(0.75,0.819) |
| Rv3133c | 3499723 | C193T | Leu65Leu | 0.019 | 1.495(1.26,1.774) |
| Rv2027c | 2273635 | T874C | Ser292Pro | 0.003 | 1.718(1.428,2.067) |
| Rv0758 | 852468 | A73C | Thr25Pro | 0.147 | 0.803(0.689,0.934) |
| Rv0079 | 88852 | A649G | Thr217Ala | 0.006 | 1.859(1.481,2.333) |
| Rv1057 | 1179424 | C29G | Ala10Gly | 0.005 | 1.879(1.498,2.358) |
| Rv0981 | 1097478 | G657A | Val219Val | 0.11 | 1.344(1.117,1.618) |
| Rv1009 | 1128400 | G310A | Asp104Asn | 0.051 | 1.868(1.357,2.573) |
| Rv2247 | 2522044 | T1302C | Ser434Ser | 0.095 | 1.706(1.239,2.349) |

OR, odds ratio; CI, confidence interval.

**Table S29.** Generalized linear mixed model analysis on clustered and non-clustered strains in the lineage4.1 cohort.

| **Gene** | **Position** | **SNP** | **Amino acid changes** | ***P* value** | **OR(95%CI)** |
| --- | --- | --- | --- | --- | --- |
| Rv1266c | 1414528 | C1313T | Ala438Val | <0.001 | 1.659(1.608,1.711) |
| Rv2984 | 3340328 | G475T | Val159Phe | <0.001 | 0.777(0.757,0.798) |
| Rv2247 | 2521699 | G957A | Val319Val | <0.001 | 1.571(1.505,1.64) |
| Rv1266c | 1414040 | G1801A | Val601Ile | <0.001 | 0.463(0.402,0.532) |
| Rv1368 | 1541356 | A337G | Arg113Gly | <0.001 | 1.361(1.26,1.47) |
| Rv0600c | 698144 | C267T | Asp89Asp | <0.001 | 2.782(2.344,3.3) |
| Rv1266c | 1414640 | C1201T | Pro401Ser | 0.006 | 0.602(0.501,0.724) |
| Rv2247 | 2521342 | T600C | Asp200Asp | <0.001 | 2.555(1.964,3.323) |
| Rv1626 | 1828455 | C276A | Thr92Thr | <0.001 | 1.372(1.267,1.484) |
| Rv1028c | 1150744 | G943A | Gly315Ser | <0.001 | 1.64(1.439,1.87) |
| Rv0601c | 698968 | C27T | Gly9Gly | <0.001 | 2.219(1.844,2.67) |
| Rv1747 | 1973649 | C20T | Ala7Val | <0.001 | 1.225(1.162,1.292) |
| Rv1813c | 2055762 | C351T | Thr117Thr | <0.001 | 1.6(1.413,1.811) |
| Rv1028c | 1151647 | C40G | Pro14Ala | <0.001 | 0.473(0.383,0.584) |
| Rv2984 | 3340300 | C447T | Pro149Pro | <0.001 | 1.634(1.516,1.761) |
| Rv1009 | 1128258 | C168T | Val56Val | 0.006 | 0.48(0.368,0.625) |
| Rv0930 | 1037012 | T14C | Met5Thr | 0.019 | 1.719(1.363,2.168) |
| Rv3245c | 3626581 | T33C | Gly11Gly | <0.001 | 1.644(1.429,1.891) |
| Rv1221 | 1364757 | G345A | Glu115Glu | <0.001 | 1.707(1.467,1.988) |
| Rv0758 | 852575 | C180T | Ile60Ile | 0.034 | 0.458(0.318,0.662) |
| Rv2984 | 3340102 | G249A | Lys83Lys | 0.14 | 0.833(0.736,0.943) |
| Rv0930 | 1037745 | C747T | Phe249Phe | 0.014 | 0.595(0.48,0.736) |
| Rv0758 | 853460 | G1065C | Glu355Asp | 0.006 | 0.49(0.377,0.636) |
| Rv0081 | 89674 | C100T | Arg34Cys | <0.001 | 0.464(0.375,0.573) |
| Rv1028c | 1149551 | G2136A | Glu712Glu | 0.044 | 1.775(1.335,2.361) |
| Rv0490 | 579578 | A230G | Glu77Gly | 0.05 | 0.486(0.337,0.702) |
| Rv0903c | 1006080 | C483T | Val161Val | 0.638 | 0.973(0.919,1.031) |
| Rv1032c | 1157458 | A498C | Arg166Arg | 0.08 | 1.259(1.104,1.435) |
| Rv0758 | 853015 | C620G | Pro207Arg | 0.038 | 0.467(0.323,0.674) |
| Rv0490 | 579456 | C108T | Arg36Arg | 0.029 | 1.225(1.116,1.344) |
| Rv0903c | 1006538 | A25C | Thr9Pro | <0.001 | 0.472(0.4,0.557) |
| Rv3132c | 3498295 | C971T | Ala324Val | 0.021 | 1.632(1.32,2.018) |
| Rv0601c | 698685 | C310T | Leu104Leu | 0.038 | 0.467(0.324,0.674) |
| Rv0490 | 580081 | C733T | His245Tyr | <0.001 | 0.467(0.378,0.578) |
| Rv1027c | 1149035 | G73A | Gly25Ser | 0.162 | 0.599(0.415,0.864) |
| Rv0491 | 581167 | G359T | Gly120Val | 0.039 | 0.468(0.324,0.676) |
| Rv3849 | 4323873 | G375A | Arg125Arg | 0.005 | 1.446(1.269,1.649) |
| Rv0195 | 231300 | C402T | Arg134Arg | 0.001 | 0.489(0.393,0.609) |
| Rv3245c | 3626025 | C589T | Leu197Leu | 0.047 | 0.483(0.335,0.697) |
| Rv0981 | 1097010 | C189T | Cys63Cys | 0.156 | 0.594(0.411,0.857) |
| Rv0602c | 699344 | C456A | Gly152Gly | 0.152 | 0.591(0.409,0.853) |
| Rv0930 | 1037073 | A75C | Arg25Arg | 0.208 | 0.629(0.436,0.909) |
| Rv0490 | 580006 | C658A | Leu220Ile | 0.008 | 1.418(1.244,1.616) |
| Rv3764c | 4210509 | G501A | Ala167Ala | 0.035 | 0.462(0.32,0.667) |
| Rv0982 | 1098599 | G1092A | Met364Ile | 0.164 | 0.6(0.416,0.867) |
| Rv1028c | 1150906 | G781A | Val261Met | 0.005 | 0.476(0.365,0.62) |
| Rv0758 | 853672 | C1277T | Ala426Val | 0.09 | 1.178(1.069,1.298) |
| Rv1027c | 1148451 | G657A | Gly219Gly | 0.04 | 0.47(0.326,0.679) |
| Rv0195 | 231390 | C492A | Asp164Glu | 0.198 | 0.623(0.432,0.899) |
| Rv0758 | 852816 | A421G | Thr141Ala | 0.035 | 0.461(0.319,0.665) |
| Rv1027c | 1148475 | C633T | Pro211Pro | 0.001 | 0.709(0.637,0.789) |
| Rv2027c | 2273873 | G636C | Ala212Ala | 0.06 | 0.497(0.343,0.72) |
| Rv2027c | 2273253 | G1256A | Arg419Gln | 0.004 | 1.697(1.411,2.042) |
| Rv3220c | 3597305 | C230T | Ala77Val | 0.039 | 0.469(0.325,0.676) |
| Rv1032c | 1157099 | G857A | Ser286Asn | 0.024 | 1.204(1.11,1.307) |
| Rv0014c | 16345 | A1126G | Thr376Ala | 0.175 | 0.608(0.421,0.877) |
| Rv0601c | 698702 | A293T | Asp98Val | 0.16 | 0.597(0.414,0.862) |
| Rv1027c | 1148682 | G426T | Lys142Asn | 0.037 | 0.465(0.322,0.671) |
| Rv3245c | 3625005 | G1609A | Val537Ile | 0.162 | 0.599(0.415,0.864) |
| Rv0014c | 17046 | C425G | Ala142Gly | 0.031 | 1.91(1.416,2.575) |
| Rv1266c | 1415618 | C223T | His75Tyr | 0.06 | 0.498(0.343,0.721) |

OR, odds ratio; CI, confidence interval.

**Table S30.** Generalized linear mixed model analysis on clustered and non-clustered strains in the lineage4.2 cohort.

| **Gene** | **Position** | **SNP** | **Amino acid changes** | ***P* value** | **OR(95%CI)** |
| --- | --- | --- | --- | --- | --- |
| Rv0758 | 852577 | C182A | Thr61Lys | <0.001 | 1.855(1.696,2.03) |
| Rv3220c | 3596416 | G1119C | Val373Val | <0.001 | 1.652(1.527,1.788) |
| Rv1032c | 1156962 | G994A | Val332Ile | 0.055 | 1.176(1.081,1.279) |
| Rv3132c | 3498364 | A902G | Asn301Ser | 0.951 | 0.983(0.749,1.29) |
| Rv0014c | 16439 | C1032T | Val344Val | 0.005 | 0.594(0.494,0.715) |
| Rv1028c | 1151004 | T683C | Leu228Pro | 0.438 | 1.234(0.941,1.618) |
| Rv3245c | 3625501 | C1113A | Val371Val | 0.001 | 1.592(1.388,1.826) |
| Rv1032c | 1156813 | G1143A | Glu381Glu | 0.182 | 1.15(1.036,1.278) |
| Rv3132c | 3499086 | G180T | Ala60Ala | 0.771 | 0.963(0.845,1.097) |
| Rv3849 | 4323821 | G323A | Gly108Glu | 0.133 | 0.664(0.506,0.872) |
| Rv1221 | 1364841 | G429A | Leu143Leu | 0.015 | 0.373(0.25,0.559) |
| Rv0600c | 698292 | G119C | Arg40Pro | 0.033 | 0.44(0.299,0.647) |
| Rv0981 | 1097412 | C591T | Val197Val | 0.031 | 0.435(0.296,0.64) |
| Rv0758 | 853361 | C966G | Asp322Glu | 0.118 | 0.653(0.497,0.858) |
| Rv0930 | 1037893 | G895T | Val299Phe | 0.003 | 1.587(1.358,1.855) |
| Rv0758 | 853360 | A965G | Asp322Gly | 0.156 | 0.58(0.395,0.851) |
| Rv0930 | 1037911 | C913T | Arg305* | <0.001 | 1.692(1.587,1.804) |
| Rv0601c | 698787 | G208A | Val70Ile | 0.115 | 1.449(1.146,1.833) |
| Rv3132c | 3498003 | G1263A | Val421Val | 0.103 | 1.446(1.154,1.813) |
| Rv0758 | 852958 | C563T | Ala188Val | 0.079 | 0.51(0.347,0.748) |
| Rv0014c | 16440 | T1031C | Val344Ala | 0.127 | 0.554(0.376,0.816) |
| Rv2984 | 3340560 | C707T | Ala236Val | 0.28 | 0.658(0.447,0.969) |
| Rv3765c | 4211659 | G126T | Ser42Ser | 0.188 | 0.745(0.596,0.931) |
| Rv2984 | 3341757 | G1904A | Gly635Asp | 0.003 | 2.264(1.721,2.977) |
| Rv0758 | 852711 | C316T | Pro106Ser | 0.643 | 0.954(0.863,1.054) |
| Rv2710 | 3022887 | A427G | Thr143Ala | 0.163 | 0.585(0.399,0.859) |
| Rv3132c | 3497724 | C1542T | Thr514Thr | 0.346 | 0.698(0.476,1.022) |
| Rv1626 | 1828626 | G447C | Leu149Phe | 0.112 | 1.247(1.085,1.433) |
| Rv0014c | 16583 | G888C | Leu296Leu | 0.737 | 0.879(0.599,1.29) |
| Rv1266c | 1414719 | G1122A | Gln374Gln | 0.238 | 0.637(0.434,0.934) |
| Rv1009 | 1128108 | C18T | Val6Val | 0.468 | 0.758(0.517,1.111) |
| Rv1690 | 1915605 | G79A | Gly27Arg | 0.31 | 0.678(0.463,0.994) |
| Rv0014c | 16331 | G1140A | Arg380Arg | 0.53 | 1.137(0.927,1.394) |
| Rv0845 | 941311 | G122T | Arg41Leu | 0.021 | 1.674(1.338,2.094) |
| Rv1032c | 1156733 | A1223G | Gln408Arg | 0.531 | 0.787(0.537,1.154) |
| Rv2710 | 3023106 | G646C | Val216Leu | 0.146 | 0.572(0.389,0.84) |
| Rv2710 | 3022655 | G195T | Arg65Arg | 0.34 | 0.695(0.474,1.018) |
| Rv2710 | 3022730 | G270A | Leu90Leu | 0.559 | 1.123(0.921,1.369) |
| Rv2984 | 3340930 | C1077T | Ala359Ala | 0.866 | 0.975(0.843,1.129) |
| Rv2247 | 2521228 | C486T | Pro162Pro | 0.052 | 1.468(1.204,1.79) |
| Rv2027c | 2273890 | T619G | Ser207Ala | 0.295 | 0.67(0.457,0.982) |
| Rv1266c | 1414365 | G1476C | Pro492Pro | 0.441 | 0.745(0.509,1.092) |
| Rv0758 | 853443 | G1048A | Gly350Ser | 0.114 | 1.829(1.249,2.68) |
| Rv0758 | 852885 | A490C | Ile164Leu | 0.135 | 0.497(0.311,0.794) |
| Rv3246c | 3627271 | G79A | Gly27Arg | 0.07 | 2.024(1.372,2.986) |
| Rv1028c | 1150449 | T1238C | Ile413Thr | 0.73 | 1.081(0.862,1.355) |
| Rv1743 | 1970413 | C1410T | Leu470Leu | 0.162 | 1.477(1.117,1.952) |
| Rv2710 | 3022751 | G291T | Leu97Leu | 0.114 | 0.477(0.298,0.763) |
| Rv0981 | 1097217 | C396T | Phe132Phe | 0.13 | 1.797(1.22,2.646) |
| Rv1743 | 1969133 | C130G | Leu44Val | 0.499 | 0.773(0.527,1.132) |
| Rv0014c | 16667 | G804A | Glu268Glu | 0.431 | 0.74(0.505,1.084) |
| Rv1033c | 1157973 | C764A | Pro255Gln | 0.746 | 0.882(0.601,1.296) |
| Rv1028c | 1149446 | G2241C | Met747Ile | 0.75 | 0.884(0.602,1.298) |
| Rv3132c | 3498666 | C600T | Val200Val | 0.262 | 0.645(0.436,0.954) |
| Rv1028c | 1150078 | A1609G | Thr537Ala | 0.305 | 0.676(0.461,0.99) |
| Rv1675c | 1900257 | G719T | Arg240Leu | 0.444 | 0.747(0.51,1.094) |
| Rv0600c | 698181 | C230T | Pro77Leu | 0.297 | 0.672(0.458,0.984) |
| Rv2027c | 2273816 | C693T | Ala231Ala | 0.428 | 0.739(0.504,1.082) |
| Rv2710 | 3023354 | T894G | Val298Val | 0.989 | 1.003(0.8,1.257) |
| Rv3245c | 3625403 | T1211C | Val404Ala | 0.336 | 1.454(0.985,2.145) |

OR, odds ratio; CI, confidence interval.

**Table S31.** Generalized linear mixed model analysis on clustered and non-clustered strains in the lineage4.4 cohort.

| **Gene** | **Position** | **SNP** | **Amino acid changes** | ***P* value** | **OR(95%CI)** |
| --- | --- | --- | --- | --- | --- |
| Rv1028c | 1149433 | T2254G | Leu752Val | <0.001 | 0.656(0.625,0.688) |
| Rv1033c | 1158549 | A188G | Asp63Gly | <0.001 | 1.531(1.455,1.611) |
| Rv1057 | 1179980 | C585A | Gly195Gly | <0.001 | 1.399(1.311,1.493) |
| Rv0758 | 853057 | C662T | Ala221Val | <0.001 | 1.527(1.406,1.657) |
| Rv0758 | 853180 | G785A | Arg262His | 0.881 | 1.017(0.906,1.142) |
| Rv0982 | 1098408 | G901A | Asp301Asn | <0.001 | 1.528(1.405,1.662) |
| Rv0981 | 1097206 | G385A | Ala129Thr | 0.001 | 0.57(0.484,0.671) |
| Rv1028c | 1150585 | C1102T | Pro368Ser | <0.001 | 2.27(1.944,2.651) |
| Rv3132c | 3499187 | C79T | Leu27Leu | <0.001 | 1.465(1.313,1.636) |
| Rv0758 | 853153 | T758G | Phe253Cys | <0.001 | 1.42(1.31,1.54) |
| Rv1743 | 1969628 | T625G | Leu209Val | 0.001 | 2.382(1.852,3.065) |
| Rv1009 | 1128567 | C477A | Val159Val | <0.001 | 2.387(1.992,2.861) |
| Rv1266c | 1415029 | C812T | Ala271Val | 0.016 | 0.546(0.424,0.701) |
| Rv0758 | 852975 | G580A | Ala194Thr | 0.01 | 0.397(0.277,0.568) |
| Rv1028c | 1150041 | G1646T | Arg549Leu | 0.005 | 0.364(0.254,0.522) |
| Rv0602c | 699262 | C538T | Leu180Leu | <0.001 | 2.337(1.974,2.768) |
| Rv0902c | 1005056 | C786A | Pro262Pro | 0.026 | 0.571(0.443,0.735) |
| Rv3245c | 3625531 | C1083T | Ala361Ala | 0.001 | 2.246(1.744,2.892) |
| Rv3220c | 3597053 | C482G | Thr161Ser | 0.156 | 0.664(0.497,0.886) |
| Rv1221 | 1364557 | T145C | Ser49Pro | 0.648 | 0.921(0.77,1.102) |
| Rv1266c | 1415549 | G292A | Gly98Ser | 0.001 | 2.316(1.8,2.98) |
| Rv1028c | 1150819 | G868A | Val290Ile | 0.016 | 0.546(0.424,0.701) |
| Rv1028c | 1149551 | G2136A | Glu712Glu | <0.001 | 1.835(1.689,1.994) |
| Rv0195 | 231287 | A389G | Tyr130Cys | 0.088 | 0.547(0.384,0.78) |
| Rv1266c | 1415259 | C582T | Tyr194Tyr | 0.076 | 0.533(0.374,0.76) |
| Rv3764c | 4210183 | G827T | Gly276Val | 0.098 | 0.557(0.391,0.793) |
| Rv1266c | 1415347 | C494T | Thr165Ile | 0.155 | 0.664(0.498,0.885) |
| Rv1747 | 1975213 | C1584T | Gly528Gly | 0.085 | 0.544(0.382,0.775) |
| Rv0902c | 1004811 | G1031T | Arg344Leu | 0.022 | 0.562(0.437,0.723) |
| Rv0600c | 698319 | T92C | Val31Ala | 0.079 | 0.537(0.377,0.766) |
| Rv1747 | 1974659 | C1030G | Pro344Ala | 0.08 | 0.538(0.378,0.766) |
| Rv1028c | 1150345 | G1342A | Ala448Thr | 0.075 | 0.533(0.374,0.759) |
| Rv0982 | 1097737 | G230C | Gly77Ala | 0.003 | 1.944(1.55,2.44) |
| Rv1368 | 1541442 | C423T | Val141Val | 0.113 | 0.57(0.4,0.813) |
| Rv0758 | 852736 | A341C | His114Pro | 0.002 | 1.326(1.208,1.455) |
| Rv1747 | 1974321 | G692A | Arg231Lys | 0.079 | 0.537(0.377,0.766) |
| Rv1032c | 1156560 | G1396A | Gly466Ser | 0.116 | 0.572(0.401,0.816) |
| Rv1057 | 1180281 | C886T | Arg296Trp | 0.079 | 0.536(0.376,0.764) |
| Rv1033c | 1158453 | G284A | Arg95Gln | 0.016 | 0.546(0.425,0.702) |
| Rv1747 | 1975703 | A2074G | Thr692Ala | 0.082 | 0.541(0.379,0.77) |
| Rv3132c | 3498219 | T1047G | Ala349Ala | 0.005 | 0.751(0.678,0.83) |
| Rv0758 | 852450 | A55G | Thr19Ala | 0.074 | 0.532(0.373,0.757) |
| Rv3220c | 3597474 | C61T | His21Tyr | 0.13 | 1.462(1.138,1.88) |
| Rv1266c | 1414242 | G1599T | Ala533Ala | 0.015 | 1.476(1.257,1.732) |
| Rv2984 | 3341608 | T1755C | Gly585Gly | 0.03 | 0.577(0.448,0.744) |
| Rv0079 | 88707 | C504A | Ala168Ala | 0.93 | 0.957(0.58,1.578) |
| Rv0490 | 580054 | A706C | Thr236Pro | 0.484 | 1.133(0.947,1.355) |
| Rv0758 | 852942 | A547G | Ser183Gly | 0.085 | 0.544(0.382,0.776) |
| Rv1813c | 2056068 | C45T | Gly15Gly | 0.034 | 1.943(1.419,2.659) |
| Rv1675c | 1900738 | A238G | Ile80Val | 0.12 | 0.576(0.403,0.822) |
| Rv0845 | 941436 | T247G | Trp83Gly | 0.078 | 0.536(0.376,0.764) |
| Rv0902c | 1005404 | G438A | Leu146Leu | 0.018 | 0.361(0.234,0.555) |
| Rv1626 | 1828317 | C138T | Asp46Asp | 0.015 | 2.375(1.665,3.387) |
| Rv1028c | 1149312 | G2375A | Gly792Asp | 0.083 | 0.541(0.38,0.771) |
| Rv1266c | 1415319 | C522T | Thr174Thr | 0.082 | 0.54(0.379,0.77) |
| Rv0758 | 853163 | C768A | Asp256Glu | 0.216 | 1.551(1.088,2.212) |
| Rv0600c | 698043 | C368G | Ala123Gly | 0.121 | 0.576(0.403,0.822) |
| Rv0757 | 851696 | C89A | Ala30Asp | 0.082 | 0.54(0.379,0.77) |
| Rv0758 | 852460 | T65G | Leu22Arg | 0.116 | 0.572(0.401,0.816) |

OR, odds ratio; CI, confidence interval.

**Table S32.** Generalized linear mixed model analysis on clustered and non-clustered strains in the lineage4.8 cohort.

| **Gene** | **Position** | **SNP** | **Amino acid changes** | ***P* value** | **OR(95%CI)** |
| --- | --- | --- | --- | --- | --- |
| Rv3764c | 4209859 | G1151T | Arg384Leu | <0.001 | 1.481(1.445,1.519) |
| Rv0014c | 15886 | C1585A | Arg529Ser | <0.001 | 0.549(0.465,0.649) |
| Rv1027c | 1148758 | G350A | Arg117Gln | <0.001 | 0.55(0.466,0.651) |
| Rv0014c | 17108 | C363T | Asp121Asp | 0.009 | 0.732(0.649,0.825) |
| Rv1028c | 1150779 | A908C | Gln303Pro | 0.002 | 0.553(0.456,0.67) |
| Rv0014c | 16807 | G664A | Val222Ile | 0.946 | 1.002(0.967,1.039) |
| Rv3132c | 3498326 | G940A | Gly314Ser | 0.051 | 0.766(0.668,0.878) |
| Rv0602c | 699466 | G334A | Gly112Ser | <0.001 | 1.443(1.365,1.527) |
| Rv3220c | 3596506 | C1029T | Ala343Ala | <0.001 | 1.411(1.3,1.531) |
| Rv3132c | 3499008 | G258A | Met86Ile | <0.001 | 0.369(0.287,0.474) |
| Rv0903c | 1005917 | G646A | Ala216Thr | <0.001 | 0.604(0.543,0.672) |
| Rv3133c | 3499910 | A6G | Val2Val | 0.001 | 1.318(1.21,1.435) |
| Rv0757 | 851825 | T218C | Met73Thr | 0.006 | 0.392(0.279,0.551) |
| Rv0079 | 88472 | C269T | Thr90Ile | 0.077 | 0.555(0.398,0.774) |
| Rv2247 | 2521342 | T600C | Asp200Asp | 0.001 | 1.343(1.232,1.464) |
| Rv2984 | 3340504 | G651A | Gln217Gln | 0.064 | 0.54(0.386,0.754) |
| Rv2027c | 2274235 | A274C | Arg92Arg | 0.075 | 0.553(0.396,0.773) |
| Rv1032c | 1157568 | G388A | Val130Ile | 0.066 | 0.542(0.389,0.759) |
| Rv0758 | 853529 | C1134T | Thr378Thr | 0.01 | 0.547(0.433,0.692) |
| Rv3764c | 4210899 | A111G | Lys37Lys | 0.076 | 0.554(0.397,0.773) |
| Rv0601c | 698797 | G198A | Ala66Ala | 0.003 | 2.737(1.941,3.861) |
| Rv1009 | 1128297 | G207A | Gln69Gln | 0.064 | 0.54(0.386,0.754) |
| Rv3246c | 3627272 | G78A | Arg26Arg | 0.076 | 0.554(0.397,0.773) |
| Rv3765c | 4211713 | G72A | Val24Val | 0.074 | 0.552(0.396,0.77) |
| Rv0601c | 698587 | G408C | Pro136Pro | 0.077 | 0.555(0.398,0.775) |
| Rv3220c | 3597121 | A414G | Arg138Arg | 0.076 | 0.553(0.397,0.772) |
| Rv1032c | 1156569 | T1387C | Ser463Pro | 0.075 | 0.551(0.395,0.77) |
| Rv3245c | 3626119 | C495T | Tyr165Tyr | 0.067 | 0.544(0.39,0.759) |
| Rv1057 | 1179501 | G106A | Gly36Ser | 0.064 | 0.54(0.387,0.754) |
| Rv2984 | 3341404 | C1551T | Asn517Asn | 0.065 | 0.542(0.388,0.756) |
| Rv0902c | 1004680 | G1162A | Gly388Ser | 0.073 | 0.551(0.395,0.769) |
| Rv0758 | 852537 | C142T | Arg48Trp | 0.072 | 0.549(0.394,0.767) |
| Rv3246c | 3627126 | C224T | Ser75Phe | 0.075 | 0.553(0.396,0.771) |
| Rv1027c | 1148952 | C156G | Asp52Glu | 0.077 | 0.555(0.398,0.775) |
| Rv3132c | 3498353 | C913A | Arg305Arg | 0.001 | 1.388(1.257,1.533) |
| Rv0757 | 851676 | C69G | Val23Val | 0.074 | 0.551(0.395,0.77) |
| Rv2027c | 2273397 | C1112T | Ala371Val | 0.067 | 0.543(0.389,0.758) |
| Rv3132c | 3498439 | T827G | Val276Gly | 0.074 | 0.552(0.395,0.77) |
| Rv1028c | 1150150 | T1537C | Leu513Leu | 0.073 | 0.549(0.393,0.767) |
| Rv1032c | 1156581 | C1375G | Arg459Gly | 0.001 | 1.895(1.57,2.286) |
| Rv1266c | 1415227 | T614C | Leu205Pro | 0.077 | 0.555(0.398,0.775) |
| Rv3132c | 3498758 | C508T | Leu170Leu | 0.073 | 0.55(0.395,0.768) |
| Rv1266c | 1415134 | G707C | Ser236Thr | 0.065 | 0.541(0.388,0.755) |
| Rv1747 | 1975441 | A1812G | Arg604Arg | 0.065 | 0.54(0.387,0.754) |
| Rv3765c | 4211323 | C462G | Arg154Arg | 0.075 | 0.553(0.396,0.771) |
| Rv0758 | 852774 | T379C | Leu127Leu | 0.006 | 0.78(0.712,0.855) |
| Rv3764c | 4210825 | C185G | Ala62Gly | 0.073 | 0.551(0.395,0.769) |
| Rv0902c | 1004542 | G1300A | Gly434Ser | 0.066 | 0.542(0.388,0.756) |
| Rv0902c | 1005752 | A90C | Val30Val | 0.074 | 0.552(0.395,0.77) |
| Rv1266c | 1415105 | T736C | Phe246Leu | 0.009 | 1.483(1.274,1.726) |
| Rv1027c | 1148611 | G497A | Arg166His | 0.073 | 0.551(0.395,0.769) |
| Rv0758 | 852543 | T148G | Leu50Val | 0.001 | 2.672(1.972,3.622) |
| Rv2027c | 2273725 | G784C | Ala262Pro | 0.015 | 1.504(1.271,1.779) |
| Rv0758 | 853697 | G1302A | Leu434Leu | 0.002 | 0.547(0.451,0.664) |
| Rv1057 | 1180217 | C822T | Ile274Ile | <0.001 | 0.553(0.468,0.654) |
| Rv0982 | 1098939 | G1432A | Gly478Ser | 0.16 | 0.703(0.547,0.903) |
| Rv1032c | 1156831 | G1125C | Val375Val | 0.709 | 1.075(0.886,1.303) |
| Rv0602c | 699202 | T598G | Cys200Gly | <0.001 | 2.489(2.113,2.933) |
| Rv1743 | 1969728 | C725T | Pro242Leu | 0.076 | 0.553(0.396,0.773) |
| Rv0195 | 231139 | G241A | Glu81Lys | <0.001 | 2.683(2.065,3.487) |
| Rv1028c | 1151044 | C643T | Arg215Cys | 0.009 | 1.578(1.326,1.878) |
| Rv1033c | 1158306 | G431A | Arg144His | 0.009 | 2.499(1.763,3.543) |

OR, odds ratio; CI, confidence interval.

**Table S33.** Generalized linear mixed model analysis on cluster size in the lineage2 cohort.

| **Gene** | **Position** | **SNP** | **Amino acid changes** | ***P* value** | **OR(95%CI)** |
| --- | --- | --- | --- | --- | --- |
| Rv0079 | 88700 | C497T | Thr166Ile | <0.001 | 1.972(1.885,2.063) |
| Rv1221 | 1364706 | G294A | Leu98Leu | <0.001 | 1.134(1.108,1.161) |
| Rv0902c | 1005093 | G749A | Arg250His | <0.001 | 4.764(4.259,5.328) |
| Rv1027c | 1148482 | C626A | Ser209* | <0.001 | 3.114(2.757,3.518) |
| Rv2247 | 2521309 | G567A | Gly189Gly | <0.001 | 5.207(4.545,5.966) |
| Rv0982 | 1098417 | G910A | Asp304Asn | <0.001 | 3.497(3.105,3.939) |
| Rv1028c | 1151304 | G383T | Arg128Leu | 0.007 | 4.821(2.694,8.628) |
| Rv0844c | 940748 | A359C | Lys120Thr | <0.001 | 2.038(1.931,2.151) |
| Rv0758 | 852953 | G558T | Pro186Pro | <0.001 | 2.452(2.23,2.697) |
| Rv2027c | 2273888 | A621G | Ser207Ser | <0.001 | 1.863(1.667,2.081) |
| Rv1027c | 1148930 | G178A | Gly60Ser | 0.266 | 0.94(0.889,0.994) |
| Rv1743 | 1969275 | T272C | Val91Ala | 0.029 | 1.224(1.115,1.343) |
| Rv2027c | 2274354 | C155T | Ala52Val | 0.636 | 0.922(0.776,1.095) |
| Rv3132c | 3498851 | C415G | His139Asp | 0.016 | 1.411(1.223,1.627) |
| Rv3765c | 4211492 | G293C | Gly98Ala | 0.057 | 1.406(1.176,1.682) |
| Rv1027c | 1149097 | T11C | Val4Ala | 0.621 | 1.107(0.902,1.359) |
| Rv3132c | 3498198 | G1068A | Glu356Glu | 0.3 | 1.381(1.011,1.887) |
| Rv2027c | 2273012 | C1497T | Ser499Ser | <0.001 | 1.701(1.516,1.908) |
| Rv1747 | 1974325 | C696A | Ile232Ile | 0.022 | 1.779(1.384,2.286) |
| Rv0845 | 941722 | T533G | Ile178Ser | 0.041 | 0.305(0.17,0.544) |
| Rv0602c | 699663 | G137A | Gly46Asp | 0.067 | 0.76(0.655,0.882) |
| Rv2984 | 3341602 | A1749C | Ala583Ala | 0.004 | 1.644(1.385,1.95) |
| Rv0757 | 852013 | C406G | Leu136Val | 0.082 | 1.55(1.204,1.994) |
| Rv0981 | 1097478 | G657A | Val219Val | 0.019 | 2.052(1.51,2.79) |
| Rv0982 | 1098287 | C780T | Thr260Thr | 0.009 | 2.052(1.559,2.702) |
| Rv0844c | 940538 | T569C | Val190Ala | 0.137 | 1.425(1.123,1.808) |
| Rv1057 | 1180048 | C653T | Ser218Leu | 0.011 | 2.477(1.733,3.54) |
| Rv1675c | 1900557 | A419C | Glu140Ala | 0.202 | 1.379(1.073,1.772) |
| Rv0490 | 580448 | G1100C | Gly367Ala | <0.001 | 1.629(1.438,1.846) |
| Rv0014c | 15890 | C1581T | Ser527Ser | 0.124 | 0.415(0.234,0.736) |
| Rv3245c | 3624954 | A1660G | Lys554Glu | 0.004 | 2.05(1.595,2.635) |
| Rv3132c | 3499257 | A9G | Thr3Thr | 0.186 | 1.598(1.122,2.277) |
| Rv0602c | 699386 | A414G | Ser138Ser | 0.019 | 1.804(1.404,2.319) |
| Rv0758 | 853215 | C820G | Leu274Val | 0.028 | 1.964(1.445,2.67) |
| Rv3245c | 3626417 | C197T | Thr66Ile | 0.053 | 1.811(1.332,2.462) |
| Rv3245c | 3626562 | C52T | Pro18Ser | 0.033 | 0.428(0.287,0.637) |
| Rv0903c | 1006262 | T301C | Leu101Leu | 0.186 | 1.598(1.122,2.277) |
| Rv1028c | 1150369 | T1318G | Leu440Val | 0.005 | 0.616(0.518,0.731) |
| Rv0845 | 941475 | T286G | Phe96Val | 0.064 | 0.749(0.641,0.875) |
| Rv1626 | 1828320 | C141T | Gly47Gly | 0.001 | 2.557(1.917,3.411) |
| Rv1743 | 1970117 | C1114T | Pro372Ser | 0.659 | 1.111(0.876,1.408) |
| Rv1009 | 1129079 | T989G | Leu330Arg | 0.045 | 0.76(0.662,0.871) |
| Rv0081 | 89670 | G96C | Arg32Arg | 0.17 | 1.209(1.052,1.381) |
| Rv1028c | 1150004 | C1683G | Val561Val | 0.818 | 1.08(0.779,1.492) |
| Rv1743 | 1969728 | C725T | Pro242Leu | 0.248 | 1.336(1.04,1.718) |
| Rv2027c | 2274003 | A506C | Tyr169Ser | 0.002 | 2.061(1.632,2.601) |
| Rv0014c | 16541 | T930A | Asp310Glu | 0.023 | 0.373(0.242,0.575) |
| Rv3132c | 3497859 | C1407T | Asp469Asp | 0.354 | 1.141(0.99,1.307) |
| Rv0490 | 580093 | G745A | Ala249Thr | 0.035 | 0.401(0.26,0.618) |
| Rv0079 | 88926 | C723G | Asp241Glu | 0.022 | 0.371(0.241,0.572) |
| Rv0195 | 230998 | G100A | Val34Met | 0.101 | 1.662(1.219,2.266) |
| Rv2984 | 3340930 | C1077T | Ala359Ala | 0.013 | 0.338(0.219,0.523) |
| Rv1032c | 1156982 | C974A | Ala325Glu | 0.025 | 0.379(0.246,0.584) |
| Rv3765c | 4211237 | C548G | Ala183Gly | 0.019 | 0.362(0.235,0.558) |
| Rv1266c | 1414365 | G1476C | Pro492Pro | 0.392 | 1.123(0.981,1.285) |
| Rv1032c | 1156754 | C1202T | Thr401Ile | 0.042 | 2.054(1.442,2.927) |
| Rv3245c | 3625328 | A1286G | Glu429Gly | 0.191 | 1.59(1.115,2.268) |
| Rv1027c | 1148591 | G517A | Gly173Ser | 0.023 | 0.374(0.243,0.577) |
| Rv1057 | 1180212 | G817T | Val273Phe | 0.109 | 1.765(1.239,2.514) |
| Rv0758 | 853066 | G671T | Gly224Val | 0.756 | 1.079(0.846,1.376) |
| Rv1266c | 1415827 | A14G | Gln5Arg | 0.118 | 0.56(0.387,0.812) |
| Rv1033c | 1158252 | G485T | Arg162Leu | 0.09 | 1.462(1.169,1.829) |
| Rv1032c | 1157070 | A886G | Ile296Val | 0.049 | 0.547(0.403,0.744) |
| Rv0758 | 852910 | C515T | Pro172Leu | 0.089 | 0.956(0.931,0.982) |
| Rv0845 | 941203 | G14C | Gly5Ala | 0.088 | 1.829(1.284,2.606) |
| Rv1747 | 1974050 | G421A | Gly141Ser | 0.351 | 1.365(0.776,2.399) |

OR, odds ratio; CI, confidence interval.

**Table S34.** Generalized linear mixed model analysis on cluster size in the lineage4 cohort.

| **Gene** | **Position** | **SNP** | **Amino acid changes** | ***P* value** | **OR(95%CI)** |
| --- | --- | --- | --- | --- | --- |
| Rv2710 | 3023036 | G576A | Glu192Glu | <0.001 | 4.807(4.591,5.033) |
| Rv3764c | 4209859 | G1151T | Arg384Leu | <0.001 | 2.656(2.547,2.77) |
| Rv0758 | 852577 | C182A | Thr61Lys | <0.001 | 2.702(2.555,2.858) |
| Rv0930 | 1037012 | T14C | Met5Thr | 0.073 | 1.562(1.218,2.004) |
| Rv1368 | 1541356 | A337G | Arg113Gly | <0.001 | 3.846(3.582,4.129) |
| Rv2247 | 2521699 | G957A | Val319Val | <0.001 | 2.54(2.389,2.699) |
| Rv1032c | 1156979 | G977T | Arg326Leu | <0.001 | 2.312(2.151,2.484) |
| Rv1028c | 1151473 | A214G | Thr72Ala | <0.001 | 4.28(3.885,4.716) |
| Rv1747 | 1973649 | C20T | Ala7Val | <0.001 | 2.221(2.075,2.377) |
| Rv1032c | 1156511 | C1445T | Ser482Leu | <0.001 | 3.149(2.954,3.357) |
| Rv3245c | 3625836 | A778G | Met260Val | <0.001 | 7.338(5.16,10.433) |
| Rv2984 | 3340328 | G475T | Val159Phe | <0.001 | 0.779(0.752,0.807) |
| Rv0195 | 231114 | C216G | Ala72Ala | <0.001 | 0.805(0.763,0.85) |
| Rv3132c | 3497874 | A1392C | Val464Val | <0.001 | 0.449(0.387,0.522) |
| Rv1028c | 1149433 | T2254G | Leu752Val | <0.001 | 0.619(0.563,0.68) |
| Rv0602c | 699466 | G334A | Gly112Ser | 0.086 | 0.55(0.388,0.78) |
| Rv2247 | 2520761 | G19A | Glu7Lys | <0.001 | 0.651(0.611,0.692) |
| Rv1028c | 1149551 | G2136A | Glu712Glu | 0.178 | 0.72(0.564,0.919) |
| Rv0014c | 16807 | G664A | Val222Ile | 0.003 | 0.844(0.797,0.893) |
| Rv0601c | 698968 | C27T | Gly9Gly | 0.001 | 0.513(0.422,0.623) |
| Rv1028c | 1150585 | C1102T | Pro368Ser | 0.055 | 1.455(1.196,1.77) |
| Rv0758 | 852782 | C387A | Arg129Arg | 0.032 | 1.273(1.138,1.423) |
| Rv0014c | 16055 | G1416T | Pro472Pro | 0.653 | 1.029(0.965,1.099) |
| Rv0600c | 698144 | C267T | Asp89Asp | <0.001 | 2.689(2.517,2.872) |
| Rv3765c | 4211448 | A337C | Ile113Leu | <0.001 | 4.349(3.924,4.821) |
| Rv2247 | 2521342 | T600C | Asp200Asp | <0.001 | 3.463(2.45,4.894) |
| Rv0930 | 1037911 | C913T | Arg305* | 0.001 | 1.948(1.606,2.363) |
| Rv1057 | 1179980 | C585A | Gly195Gly | 0.013 | 1.235(1.134,1.344) |
| Rv0758 | 853579 | C1184G | Ala395Gly | 0.001 | 1.181(1.122,1.242) |
| Rv3132c | 3498353 | C913A | Arg305Arg | 0.105 | 1.279(1.099,1.489) |
| Rv0982 | 1098698 | C1191G | Gly397Gly | 0.003 | 1.198(1.126,1.275) |
| Rv0982 | 1098408 | G901A | Asp301Asn | 0.001 | 1.415(1.269,1.578) |
| Rv1009 | 1128567 | C477A | Val159Val | 0.001 | 2.293(1.766,2.977) |
| Rv1626 | 1828455 | C276A | Thr92Thr | <0.001 | 1.614(1.436,1.815) |
| Rv0844c | 940838 | C269G | Ala90Gly | 0.003 | 0.552(0.451,0.676) |
| Rv0758 | 853057 | C662T | Ala221Val | 0.001 | 1.413(1.274,1.568) |
| Rv1813c | 2055950 | A163G | Ile55Val | <0.001 | 0.449(0.364,0.553) |
| Rv1813c | 2055762 | C351T | Thr117Thr | 0.001 | 1.774(1.501,2.096) |
| Rv1027c | 1148762 | G346C | Val116Leu | 0.021 | 0.665(0.558,0.793) |
| Rv0758 | 852910 | C515T | Pro172Leu | 0.002 | 0.306(0.207,0.452) |
| Rv2984 | 3339968 | G115A | Ala39Thr | <0.001 | 0.513(0.432,0.61) |
| Rv3132c | 3499086 | G180T | Ala60Ala | 0.001 | 0.511(0.418,0.624) |
| Rv3132c | 3498714 | C552G | Asp184Glu | 0.001 | 2.737(2.028,3.695) |
| Rv0758 | 852736 | A341C | His114Pro | 0.79 | 1.038(0.905,1.19) |
| Rv0902c | 1004729 | G1113A | Arg371Arg | 0.001 | 0.513(0.42,0.627) |
| Rv1028c | 1150744 | G943A | Gly315Ser | 0.001 | 1.8(1.51,2.147) |
| Rv0758 | 853180 | G785A | Arg262His | 0.25 | 1.158(1.019,1.317) |
| Rv1221 | 1364757 | G345A | Glu115Glu | 0.003 | 1.818(1.484,2.228) |
| Rv0601c | 698797 | G198A | Ala66Ala | 0.096 | 1.262(1.097,1.452) |
| Rv1032c | 1157099 | G857A | Ser286Asn | 0.758 | 1.035(0.927,1.155) |
| Rv3765c | 4211695 | T90C | Tyr30Tyr | <0.001 | 2.022(1.706,2.396) |
| Rv1057 | 1180375 | C980T | Thr327Met | <0.001 | 0.513(0.445,0.591) |
| Rv1368 | 1541074 | A55C | Ser19Arg | 0.058 | 0.519(0.367,0.733) |
| Rv1028c | 1149705 | A1982T | Glu661Val | 0.007 | 2.179(1.632,2.91) |
| Rv1028c | 1149367 | C2320T | Arg774Trp | 0.009 | 2.136(1.6,2.852) |
| Rv0600c | 698096 | C315T | Leu105Leu | 0.009 | 2.134(1.598,2.849) |
| Rv0490 | 580506 | C1158T | Asp386Asp | 0.052 | 0.51(0.361,0.721) |
| Rv0758 | 852774 | T379C | Leu127Leu | 0.57 | 1.083(0.942,1.246) |
| Rv2710 | 3023354 | T894G | Val298Val | 0.051 | 0.509(0.36,0.72) |
| Rv0014c | 15780 | C1691T | Pro564Leu | 0.06 | 0.521(0.369,0.736) |
| Rv3765c | 4211206 | C579T | Asp193Asp | 0.056 | 1.198(1.09,1.318) |

OR, odds ratio; CI, confidence interval.

**Table S35.** Generalized linear mixed model analysis on cross-country strains in the lineage2 cohort.

| **Gene** | **Position** | **SNP** | **Amino acid changes** | ***P* value** | **OR(95%CI)** |
| --- | --- | --- | --- | --- | --- |
| Rv0982 | 1098417 | G910A | Asp304Asn | <0.001 | 1.592 (1.53, 1.657) |
| Rv0982 | 1098824 | C1317G | Gly439Gly | <0.001 | 1.536 (1.426, 1.654) |
| Rv1057 | 1180212 | G817T | Val273Phe | <0.001 | 2.452 (2.083, 2.886) |
| Rv1743 | 1969837 | G834A | Ala278Ala | <0.001 | 1.692 (1.557, 1.839) |
| Rv3132c | 3497859 | C1407T | Asp469Asp | <0.001 | 1.297 (1.209, 1.391) |
| Rv0079 | 88700 | C497T | Thr166Ile | <0.001 | 0.885 (0.867, 0.904) |
| Rv1028c | 1151304 | G383T | Arg128Leu | <0.001 | 2.46 (1.994, 3.034) |
| Rv1747 | 1974002 | T373G | Ser125Ala | 0.001 | 2.529 (1.908, 3.353) |
| Rv0014c | 16978 | G493C | Asp165His | 0.004 | 2.23 (1.682, 2.956) |
| Rv1266c | 1414479 | C1362A | Ala454Ala | <0.001 | 2.399 (1.966, 2.927) |
| Rv0845 | 941722 | T533G | Ile178Ser | <0.001 | 0.426 (0.344, 0.526) |
| Rv0903c | 1006549 | A14G | Asp5Gly | 0.001 | 2.535 (1.912, 3.36) |
| Rv0758 | 852910 | C515T | Pro172Leu | 0.704 | 0.995 (0.983, 1.007) |
| Rv1028c | 1150441 | G1246C | Ala416Pro | 0.004 | 2.241 (1.69, 2.971) |
| Rv0758 | 852970 | A575C | Gln192Pro | 0.004 | 2.241 (1.689, 2.974) |
| Rv0490 | 580456 | T1108G | Ser370Ala | 0.002 | 2.37 (1.788, 3.142) |
| Rv1221 | 1364706 | G294A | Leu98Leu | 0.003 | 0.951 (0.935, 0.968) |
| Rv0902c | 1004606 | G1236A | Leu412Leu | <0.001 | 2.635 (2.158, 3.219) |
| Rv1743 | 1970117 | C1114T | Pro372Ser | 0.072 | 1.214 (1.09, 1.353) |
| Rv0079 | 88493 | G290T | Arg97Leu | 0.001 | 2.568 (1.937, 3.404) |
| Rv0758 | 853090 | C695T | Ala232Val | 0.052 | 1.758 (1.315, 2.349) |
| Rv0602c | 699644 | T156C | Val52Val | 0.161 | 1.502 (1.123, 2.01) |
| Rv1675c | 1900273 | G703C | Asp235His | 0.001 | 2.512 (1.895, 3.33) |
| Rv0490 | 580448 | G1100C | Gly367Ala | 0.107 | 1.097 (1.036, 1.163) |
| Rv1027c | 1148930 | G178A | Gly60Ser | <0.001 | 1.076 (1.055, 1.096) |
| Rv1690 | 1915737 | T211C | Ser71Pro | <0.001 | 2.721 (2.052, 3.607) |
| Rv0758 | 853066 | G671T | Gly224Val | 0.144 | 1.178 (1.053, 1.318) |
| Rv0758 | 852468 | A73C | Thr25Pro | 0.101 | 0.76 (0.643, 0.899) |
| Rv3764c | 4210086 | T924C | Arg308Arg | 0.521 | 0.88 (0.721, 1.074) |
| Rv0845 | 942054 | C865T | Leu289Leu | 0.206 | 1.547 (1.095, 2.184) |
| Rv2710 | 3023201 | C741G | Ile247Met | 0.102 | 0.621 (0.464, 0.831) |
| Rv2027c | 2273012 | C1497T | Ser499Ser | 0.001 | 0.848 (0.806, 0.892) |
| Rv1747 | 1975876 | C2247A | Ala749Ala | 0.084 | 1.654 (1.236, 2.212) |
| Rv3220c | 3596942 | T593G | Leu198Arg | 0.114 | 1.565 (1.178, 2.079) |
| Rv1368 | 1541798 | C779T | Ala260Val | 0.9 | 0.997 (0.973, 1.021) |
| Rv0757 | 851982 | G375T | Leu125Phe | 0.097 | 0.717 (0.587, 0.876) |
| Rv0014c | 16987 | G484A | Ala162Thr | 0.039 | 0.558 (0.42, 0.741) |
| Rv1027c | 1148482 | C626A | Ser209* | 0.451 | 0.96 (0.908, 1.014) |
| Rv2027c | 2273888 | A621G | Ser207Ser | 0.018 | 0.892 (0.85, 0.936) |
| Rv1032c | 1157543 | T413C | Val138Ala | 0.041 | 0.65 (0.526, 0.803) |
| Rv0758 | 852953 | G558T | Pro186Pro | 0.054 | 0.919 (0.88, 0.961) |
| Rv3132c | 3499134 | C132T | Gly44Gly | <0.001 | 0.892 (0.875, 0.909) |
| Rv1028c | 1150369 | T1318G | Leu440Val | 0.233 | 0.91 (0.841, 0.985) |
| Rv3764c | 4210838 | A172G | Arg58Gly | 0.045 | 0.909 (0.868, 0.953) |
| Rv3765c | 4211492 | G293C | Gly98Ala | 0.436 | 0.938 (0.864, 1.018) |
| Rv0902c | 1005093 | G749A | Arg250His | 0.658 | 0.977 (0.929, 1.028) |
| Rv1027c | 1149097 | T11C | Val4Ala | 0.042 | 0.825 (0.751, 0.908) |
| Rv2027c | 2274354 | C155T | Ala52Val | 0.029 | 0.841 (0.777, 0.91) |
| Rv1009 | 1128883 | G793A | Val265Met | 0.296 | 1.046 (1.002, 1.092) |
| Rv2984 | 3340333 | C480T | Thr160Thr | 0.447 | 0.931 (0.846, 1.023) |
| Rv3220c | 3596614 | C921T | Asn307Asn | 0.042 | 1.502 (1.23, 1.835) |
| Rv0845 | 941475 | T286G | Phe96Val | 0.494 | 0.951 (0.884, 1.023) |
| Rv0844c | 940748 | A359C | Lys120Thr | 0.378 | 0.96 (0.916, 1.006) |
| Rv3132c | 3498851 | C415G | His139Asp | 0.093 | 0.896 (0.839, 0.956) |
| Rv1675c | 1900557 | A419C | Glu140Ala | 0.516 | 0.928 (0.826, 1.042) |
| Rv0902c | 1004823 | C1019T | Pro340Leu | <0.001 | 0.326 (0.238, 0.447) |
| Rv0490 | 579740 | G392A | Arg131Gln | 0.419 | 0.932 (0.856, 1.016) |
| Rv0081 | 89670 | G96C | Arg32Arg | 0.194 | 0.908 (0.844, 0.978) |
| Rv1747 | 1974050 | G421A | Gly141Ser | 0.386 | 0.896 (0.789, 1.017) |
| Rv1747 | 1975225 | G1596C | Ala532Ala | 0.584 | 0.926 (0.804, 1.066) |
| Rv0490 | 579964 | C616A | Arg206Arg | 0.225 | 0.842 (0.731, 0.969) |
| Rv1690 | 1915768 | G242C | Gly81Ala | 0.475 | 0.956 (0.899, 1.017) |
| Rv0844c | 940570 | A537G | Leu179Leu | 0.221 | 0.819 (0.696, 0.964) |

OR, odds ratio; CI, confidence interval.

**Table S36.** Generalized linear mixed model analysis on cross-country strains in the lineage4 cohort.

| **Gene** | **Position** | **SNP** | **Amino acid changes** | ***P* value** | **OR(95%CI)** |
| --- | --- | --- | --- | --- | --- |
| Rv0758 | 852782 | C387A | Arg129Arg | <0.001 | 2.164(2.056,2.277) |
| Rv3764c | 4209859 | G1151T | Arg384Leu | <0.001 | 0.79(0.775,0.805) |
| Rv1032c | 1157768 | T188G | Leu63Arg | <0.001 | 1.598(1.534,1.665) |
| Rv0758 | 853153 | T758G | Phe253Cys | <0.001 | 1.826(1.735,1.921) |
| Rv0981 | 1097136 | C315T | Leu105Leu | <0.001 | 2.612(2.401,2.841) |
| Rv1057 | 1180375 | C980T | Thr327Met | <0.001 | 1.713(1.56,1.879) |
| Rv3765c | 4211695 | T90C | Tyr30Tyr | 0.011 | 0.795(0.725,0.87) |
| Rv0081 | 89767 | G193T | Ala65Ser | 0.014 | 1.146(1.083,1.212) |
| Rv0758 | 852910 | C515T | Pro172Leu | <0.001 | 0.39(0.327,0.465) |
| Rv1368 | 1541635 | A616C | Lys206Gln | <0.001 | 2.217(1.946,2.524) |
| Rv1032c | 1156979 | G977T | Arg326Leu | <0.001 | 1.255(1.214,1.297) |
| Rv2027c | 2273748 | T761C | Val254Ala | <0.001 | 2.578(2.203,3.016) |
| Rv2247 | 2521342 | T600C | Asp200Asp | 0.996 | 0.999(0.854,1.169) |
| Rv3132c | 3498295 | C971T | Ala324Val | <0.001 | 2.617(2.303,2.974) |
| Rv1032c | 1157385 | G571A | Val191Ile | <0.001 | 1.896(1.761,2.042) |
| Rv0195 | 231114 | C216G | Ala72Ala | <0.001 | 0.856(0.836,0.875) |
| Rv1266c | 1414119 | G1722A | Thr574Thr | <0.001 | 1.581(1.462,1.709) |
| Rv0982 | 1098830 | G1323A | Leu441Leu | <0.001 | 1.96(1.802,2.132) |
| Rv0014c | 16807 | G664A | Val222Ile | <0.001 | 0.89(0.867,0.913) |
| Rv0930 | 1037739 | C741T | Asp247Asp | <0.001 | 2.522(2.153,2.954) |
| Rv3245c | 3625065 | A1549C | Met517Leu | 0.127 | 0.965(0.942,0.988) |
| Rv0602c | 699466 | G334A | Gly112Ser | 0.012 | 0.912(0.879,0.946) |
| Rv1028c | 1149932 | G1755A | Met585Ile | <0.001 | 2.065(1.941,2.197) |
| Rv0758 | 852722 | T327G | Asn109Lys | <0.001 | 2.641(2.117,3.294) |
| Rv2984 | 3340615 | C762G | Phe254Leu | 0.071 | 0.946(0.917,0.975) |
| Rv1626 | 1828204 | G25A | Asp9Asn | <0.001 | 2.588(2.075,3.228) |
| Rv2247 | 2520923 | G181A | Gly61Ser | <0.001 | 2.651(2.125,3.307) |
| Rv1027c | 1148727 | G381A | Gln127Gln | <0.001 | 2.588(2.075,3.228) |
| Rv0844c | 940948 | T159C | His53His | <0.001 | 2.646(2.121,3.3) |
| Rv3132c | 3498853 | C413A | Ala138Glu | <0.001 | 2.633(2.252,3.077) |
| Rv0757 | 851634 | G27C | Thr9Thr | <0.001 | 2.612(2.234,3.053) |
| Rv0079 | 88797 | A594G | Ser198Ser | <0.001 | 0.361(0.305,0.428) |
| Rv0758 | 852679 | C284G | Ala95Gly | <0.001 | 2.588(2.075,3.228) |
| Rv1266c | 1415347 | C494T | Thr165Ile | <0.001 | 2.625(2.349,2.933) |
| Rv0902c | 1005116 | G726A | Leu242Leu | <0.001 | 2.575(2.201,3.013) |
| Rv1747 | 1975741 | C2112T | Thr704Thr | <0.001 | 2.643(2.261,3.089) |
| Rv0014c | 16435 | G1036C | Val346Leu | 0.014 | 0.799(0.728,0.875) |
| Rv0902c | 1005068 | C774A | Thr258Thr | <0.001 | 2.612(2.094,3.258) |
| Rv0930 | 1037355 | T357C | Thr119Thr | 0.05 | 0.981(0.971,0.991) |
| Rv3132c | 3498353 | C913A | Arg305Arg | 0.001 | 0.8(0.747,0.857) |
| Rv0601c | 698656 | C339T | Thr113Thr | <0.001 | 1.603(1.422,1.808) |
| Rv1028c | 1149551 | G2136A | Glu712Glu | 0.971 | 0.999(0.969,1.03) |
| Rv0902c | 1004729 | G1113A | Arg371Arg | 0.001 | 1.365(1.246,1.495) |
| Rv3764c | 4210274 | T736C | Cys246Arg | <0.001 | 1.194(1.164,1.224) |
| Rv1033c | 1158306 | G431A | Arg144His | <0.001 | 2.177(1.744,2.718) |
| Rv2984 | 3341308 | C1455T | Leu485Leu | 0.002 | 1.207(1.135,1.283) |
| Rv1028c | 1151062 | G625C | Ala209Pro | <0.001 | 2.627(2.248,3.071) |
| Rv2247 | 2521442 | C700T | Leu234Leu | 0.016 | 1.714(1.372,2.143) |
| Rv3245c | 3626260 | T354C | Ala118Ala | 0.015 | 1.721(1.377,2.151) |
| Rv2247 | 2520778 | G36A | Ser12Ser | 0.015 | 1.716(1.373,2.145) |
| Rv3245c | 3625603 | G1011A | Glu337Glu | 0.015 | 1.718(1.374,2.147) |
| Rv2027c | 2273725 | G784C | Ala262Pro | 0.041 | 0.796(0.712,0.89) |
| Rv1266c | 1415509 | T332G | Leu111Arg | 0.016 | 1.714(1.372,2.143) |
| Rv2027c | 2274184 | A325G | Ile109Val | <0.001 | 0.469(0.393,0.558) |
| Rv0014c | 15899 | G1572C | Ser524Ser | <0.001 | 0.466(0.391,0.555) |
| Rv0014c | 17378 | C93T | Leu31Leu | <0.001 | 2.158(1.844,2.524) |
| Rv0845 | 941190 | G1C | Val1? | <0.001 | 0.634(0.556,0.723) |
| Rv3849 | 4323513 | C15T | Phe5Phe | 0.015 | 1.718(1.374,2.147) |
| Rv0982 | 1098089 | C582T | Arg194Arg | 0.005 | 0.639(0.545,0.749) |
| Rv1266c | 1415105 | T736C | Phe246Leu | 0.006 | 1.32(1.194,1.461) |

OR, odds ratio; CI, confidence interval.

**Table S37.** Generalized linear mixed model analysis on cross-regional strains in the lineage2 cohort.

| **Gene** | **Position** | **SNP** | **Amino acid changes** | ***P* value** | **OR(95%CI)** |
| --- | --- | --- | --- | --- | --- |
| Rv0982 | 1098417 | G910A | Asp304Asn | <0.001 | 1.567(1.507,1.629) |
| Rv0982 | 1098824 | C1317G | Gly439Gly | <0.001 | 1.587(1.48,1.702) |
| Rv0902c | 1004606 | G1236A | Leu412Leu | <0.001 | 2.633(2.166,3.2) |
| Rv1032c | 1157070 | A886G | Ile296Val | 0.002 | 1.527(1.328,1.754) |
| Rv1057 | 1180212 | G817T | Val273Phe | <0.001 | 2.452(2.09,2.878) |
| Rv1221 | 1364706 | G294A | Leu98Leu | 0.009 | 0.955(0.938,0.972) |
| Rv1032c | 1157208 | G748A | Val250Ile | <0.001 | 2.309(1.898,2.809) |
| Rv3132c | 3497859 | C1407T | Asp469Asp | <0.001 | 1.256(1.177,1.34) |
| Rv3132c | 3498420 | C846T | Ser282Ser | <0.001 | 1.652(1.523,1.791) |
| Rv0079 | 88700 | C497T | Thr166Ile | <0.001 | 0.885(0.867,0.904) |
| Rv1028c | 1151304 | G383T | Arg128Leu | <0.001 | 2.457(2.319,2.604) |
| Rv1057 | 1180531 | A1136G | Gln379Arg | 0.001 | 2.583(1.958,3.408) |
| Rv0845 | 941722 | T533G | Ile178Ser | <0.001 | 0.421(0.396,0.448) |
| Rv0490 | 580023 | G675C | Arg225Arg | <0.001 | 2.68(2.032,3.536) |
| Rv1747 | 1974002 | T373G | Ser125Ala | 0.001 | 2.601(1.97,3.435) |
| Rv0014c | 16978 | G493C | Asp165His | 0.004 | 2.232(1.692,2.945) |
| Rv0079 | 88493 | G290T | Arg97Leu | 0.001 | 2.588(1.962,3.414) |
| Rv3132c | 3497979 | A1287C | Val429Val | 0.003 | 2.255(1.709,2.974) |
| Rv3133c | 3499512 | C404T | Ala135Val | 0.001 | 2.418(1.833,3.19) |
| Rv2710 | 3022835 | A375C | Arg125Arg | 0.034 | 1.514(1.245,1.842) |
| Rv1690 | 1915737 | T211C | Ser71Pro | 0.382 | 1.115(0.985,1.262) |
| Rv1368 | 1541798 | C779T | Ala260Val | 0.973 | 0.999(0.974,1.024) |
| Rv2027c | 2273166 | C1343T | Ser448Phe | 0.003 | 2.277(1.725,3.007) |
| Rv1027c | 1148930 | G178A | Gly60Ser | <0.001 | 1.075(1.053,1.096) |
| Rv3132c | 3497670 | C1596T | Arg532Arg | 0.651 | 1.194(0.807,1.766) |
| Rv0757 | 851982 | G375T | Leu125Phe | 0.102 | 0.725(0.596,0.883) |
| Rv0490 | 580448 | G1100C | Gly367Ala | 0.081 | 1.105(1.044,1.17) |
| Rv2710 | 3023201 | C741G | Ile247Met | 0.074 | 0.6(0.452,0.799) |
| Rv1009 | 1128610 | G520A | Asp174Asn | 0.962 | 1.019(0.689,1.508) |
| Rv2027c | 2273012 | C1497T | Ser499Ser | 0.001 | 0.853(0.811,0.897) |
| Rv1009 | 1128883 | G793A | Val265Met | 0.243 | 0.975(0.954,0.997) |
| Rv1027c | 1148547 | G561A | Ala187Ala | 0.001 | 2.484(1.883,3.277) |
| Rv0758 | 852780 | C385A | Arg129Ser | 0.046 | 1.763(1.327,2.342) |
| Rv0845 | 941475 | T286G | Phe96Val | 0.407 | 0.943(0.878,1.012) |
| Rv1028c | 1149316 | G2371A | Glu791Lys | 0.577 | 1.029(0.977,1.084) |
| Rv0845 | 942054 | C865T | Leu289Leu | 0.197 | 1.548(1.103,2.173) |
| Rv1028c | 1150369 | T1318G | Leu440Val | 0.27 | 0.918(0.849,0.992) |
| Rv0758 | 852529 | G134A | Arg45Gln | 0.097 | 1.59(1.203,2.102) |
| Rv2984 | 3340333 | C480T | Thr160Thr | 0.494 | 0.938(0.855,1.029) |
| Rv3220c | 3596942 | T593G | Leu198Arg | 0.096 | 1.592(1.204,2.104) |
| Rv0844c | 940522 | G585A | Glu195Glu | 0.241 | 1.398(1.05,1.861) |
| Rv3765c | 4211492 | G293C | Gly98Ala | 0.49 | 0.946(0.872,1.025) |
| Rv0014c | 16089 | C1382G | Pro461Arg | <0.001 | 0.896(0.877,0.915) |
| Rv0490 | 579740 | G392A | Arg131Gln | 0.468 | 0.941(0.864,1.024) |
| Rv2027c | 2274354 | C155T | Ala52Val | 0.033 | 0.847(0.783,0.916) |
| Rv3764c | 4210838 | A172G | Arg58Gly | 0.04 | 0.908(0.867,0.952) |
| Rv0758 | 852953 | G558T | Pro186Pro | 0.048 | 0.919(0.88,0.959) |
| Rv2027c | 2273888 | A621G | Ser207Ser | 0.016 | 0.892(0.85,0.936) |
| Rv3764c | 4210086 | T924C | Arg308Arg | 0.033 | 1.383(1.188,1.61) |
| Rv0902c | 1005093 | G749A | Arg250His | 0.623 | 0.975(0.928,1.025) |
| Rv1747 | 1974050 | G421A | Gly141Ser | 0.418 | 0.904(0.799,1.023) |
| Rv1027c | 1148482 | C626A | Ser209* | 0.428 | 0.958(0.908,1.011) |
| Rv1675c | 1900557 | A419C | Glu140Ala | 0.557 | 0.935(0.834,1.048) |
| Rv0758 | 853066 | G671T | Gly224Val | 0.102 | 1.197(1.073,1.336) |
| Rv1690 | 1915768 | G242C | Gly81Ala | 0.451 | 0.955(0.899,1.015) |
| Rv3132c | 3498851 | C415G | His139Asp | 0.087 | 0.896(0.84,0.955) |
| Rv0081 | 89670 | G96C | Arg32Arg | 0.66 | 0.972(0.912,1.037) |
| Rv1747 | 1975708 | C2079T | Thr693Thr | 0.699 | 0.94(0.801,1.103) |
| Rv0844c | 940848 | C259T | Leu87Phe | 0.244 | 0.865(0.764,0.979) |

OR, odds ratio; CI, confidence interval.

**Table S38.** Generalized linear mixed model analysis on cross-regional strains in the lineage4 cohort.

| **Gene** | **Position** | **SNP** | **Amino acid changes** | ***P* value** | **OR(95%CI)** |
| --- | --- | --- | --- | --- | --- |
| Rv0758 | 852782 | C387A | Arg129Arg | <0.001 | 2.071 (1.974, 2.173) |
| Rv3764c | 4209859 | G1151T | Arg384Leu | <0.001 | 0.758 (0.745, 0.772) |
| Rv0758 | 853153 | T758G | Phe253Cys | <0.001 | 1.84 (1.754, 1.931) |
| Rv1032c | 1157768 | T188G | Leu63Arg | <0.001 | 1.634 (1.573, 1.697) |
| Rv1028c | 1149932 | G1755A | Met585Ile | <0.001 | 2.096 (1.978, 2.221) |
| Rv2247 | 2521342 | T600C | Asp200Asp | 0.997 | 1 (0.897, 1.115) |
| Rv1266c | 1414119 | G1722A | Thr574Thr | <0.001 | 1.603 (1.489, 1.726) |
| Rv0758 | 852910 | C515T | Pro172Leu | <0.001 | 0.39 (0.361, 0.421) |
| Rv0981 | 1097136 | C315T | Leu105Leu | <0.001 | 2.675 (2.472, 2.895) |
| Rv0195 | 231114 | C216G | Ala72Ala | <0.001 | 0.815 (0.799, 0.83) |
| Rv3765c | 4211695 | T90C | Tyr30Tyr | 0.122 | 1.131 (1.045, 1.224) |
| Rv1057 | 1180375 | C980T | Thr327Met | 0.005 | 1.26 (1.161, 1.368) |
| Rv1032c | 1157385 | G571A | Val191Ile | <0.001 | 1.91 (1.781, 2.048) |
| Rv0014c | 16807 | G664A | Val222Ile | <0.001 | 0.86 (0.838, 0.882) |
| Rv1032c | 1156979 | G977T | Arg326Leu | <0.001 | 1.251 (1.213, 1.29) |
| Rv2027c | 2273748 | T761C | Val254Ala | <0.001 | 2.627 (2.266, 3.047) |
| Rv1028c | 1150421 | G1266C | Met422Ile | <0.001 | 2.649 (2.284, 3.071) |
| Rv1368 | 1541635 | A616C | Lys206Gln | <0.001 | 2.147 (1.9, 2.425) |
| Rv2984 | 3340615 | C762G | Phe254Leu | 0.131 | 0.956 (0.928, 0.985) |
| Rv0757 | 851634 | G27C | Thr9Thr | <0.001 | 2.675 (2.307, 3.102) |
| Rv1028c | 1151062 | G625C | Ala209Pro | <0.001 | 2.686 (2.316, 3.114) |
| Rv2984 | 3341291 | C1438T | His480Tyr | <0.001 | 2.664 (2.298, 3.089) |
| Rv0602c | 699466 | G334A | Gly112Ser | <0.001 | 2.394 (2.063, 2.779) |
| Rv0081 | 89767 | G193T | Ala65Ser | 0.058 | 1.102 (1.047, 1.16) |
| Rv3132c | 3498353 | C913A | Arg305Arg | <0.001 | 0.764 (0.716, 0.815) |
| Rv1626 | 1828204 | G25A | Asp9Asn | <0.001 | 2.633 (2.136, 3.245) |
| Rv0902c | 1004729 | G1113A | Arg371Arg | <0.001 | 1.449 (1.319, 1.592) |
| Rv1027c | 1148727 | G381A | Gln127Gln | <0.001 | 2.633 (2.136, 3.245) |
| Rv1747 | 1975741 | C2112T | Thr704Thr | <0.001 | 2.408 (2.075, 2.795) |
| Rv3245c | 3625065 | A1549C | Met517Leu | 0.004 | 0.957 (0.943, 0.971) |
| Rv0758 | 853200 | C805T | Arg269Cys | <0.001 | 2.627 (2.132, 3.238) |
| Rv2710 | 3023035 | A575C | Glu192Ala | <0.001 | 2.63 (2.134, 3.241) |
| Rv1813c | 2055684 | C429T | Asn143Asn | <0.001 | 2.662 (2.16, 3.281) |
| Rv0014c | 16904 | G567A | Arg189Arg | <0.001 | 2.392 (1.939, 2.951) |
| Rv0014c | 16435 | G1036C | Val346Leu | 0.002 | 0.763 (0.699, 0.832) |
| Rv0758 | 852689 | C294A | Asp98Glu | <0.001 | 2.284 (1.842, 2.832) |
| Rv3245c | 3626224 | G390A | Val130Val | <0.001 | 2.625 (2.264, 3.043) |
| Rv2247 | 2520778 | G36A | Ser12Ser | 0.013 | 1.685 (1.365, 2.081) |
| Rv2984 | 3340328 | G475T | Val159Phe | 0.247 | 0.989 (0.979, 0.999) |
| Rv1033c | 1158306 | G431A | Arg144His | <0.001 | 2.077 (1.684, 2.563) |
| Rv3764c | 4210274 | T736C | Cys246Arg | <0.001 | 1.245 (1.215, 1.275) |
| Rv1743 | 1970394 | G1391A | Gly464Glu | 0.834 | 0.974 (0.862, 1.101) |
| Rv1266c | 1415509 | T332G | Leu111Arg | 0.013 | 1.685 (1.365, 2.081) |
| Rv0601c | 698656 | C339T | Thr113Thr | <0.001 | 1.606 (1.435, 1.799) |
| Rv1368 | 1541171 | C152T | Pro51Leu | 0.057 | 0.754 (0.649, 0.875) |
| Rv0758 | 853579 | C1184G | Ala395Gly | <0.001 | 1.094 (1.07, 1.119) |
| Rv0014c | 15899 | G1572C | Ser524Ser | 0.012 | 0.666 (0.566, 0.783) |
| Rv0014c | 16244 | G1227T | Ser409Ser | 0.001 | 0.619 (0.533, 0.719) |
| Rv2984 | 3341308 | C1455T | Leu485Leu | <0.001 | 1.215 (1.149, 1.285) |
| Rv0758 | 852774 | T379C | Leu127Leu | <0.001 | 0.758 (0.714, 0.805) |
| Rv0844c | 940682 | G425A | Gly142Glu | <0.001 | 1.774 (1.614, 1.948) |
| Rv0758 | 852582 | C187T | Pro63Ser | <0.001 | 0.366 (0.315, 0.426) |
| Rv3220c | 3596506 | C1029T | Ala343Ala | 0.02 | 0.882 (0.836, 0.931) |
| Rv0601c | 698797 | G198A | Ala66Ala | <0.001 | 0.757 (0.713, 0.804) |
| Rv1675c | 1900242 | G734A | Ter245Ter | 0.082 | 0.672 (0.534, 0.845) |
| Rv3246c | 3627011 | T339C | Val113Val | 0.188 | 0.759 (0.615, 0.936) |
| Rv0601c | 698763 | C232G | Gln78Glu | 0.024 | 0.621 (0.503, 0.767) |
| Rv0982 | 1097733 | C226T | Pro76Ser | 0.001 | 0.761 (0.703, 0.824) |
| Rv0014c | 17108 | C363T | Asp121Asp | 0.056 | 0.753 (0.649, 0.874) |
| Rv1266c | 1415184 | C657T | Ser219Ser | 0.192 | 0.761 (0.617, 0.939) |

OR, odds ratio; CI, confidence interval.
