# Supplementary material for: Association between two-component systems gene mutation and Mycobacterium tuberculosis transmission revealed by whole genome sequencing
Source: BMC Genomics. 2023 Nov 28;24:718. doi: 10.1186/s12864-023-09788-2 (PMC10683263; doi:10.1186/s12864-023-09788-2)
Supplement: Supplementary file 23 — Supplementary Material 23: Legends of Additional files [file 12864_2023_9788_MOESM23_ESM.docx]

**Supplementary Information**

**Additional file 1:** **Fig. S1.** The phylogenetic tree analysis of lineage4.1. **Fig. S2.** The phylogenetic tree analysis of lineage4.2. **Fig. S3.** The phylogenetic tree analysis of lineage4.3. **Fig. S4.** The phylogenetic tree analysis of lineage4.4. **Fig. S5.** The phylogenetic tree analysis of lineage4.8. **Fig. S6.** ROC curve analysis was conducted to evaluate the performance of models for cluster analysis within lineage 1. (A) ROC analysis showing the performance of the random forest model. (B) ROC analysis showing the performance of the gradient boosting decision tree. **Fig. S7.** ROC curve analysis was conducted to evaluate the performance of models for cluster analysis within lineage 3. (A) ROC analysis showing the performance of the random forest model. (B) ROC analysis showing the performance of the gradient boosting decision tree. **Fig. S8.** ROC curve analysis was conducted to evaluate the performance of models for cluster analysis within lineage 4. (A) ROC analysis showing the performance of the random forest model. (B) ROC analysis showing the performance of the gradient boosting decision tree. **Fig. S9.** ROC curve analysis was conducted to evaluate the performance of models for cluster analysis within lineage 2.2.1. (A) ROC analysis showing the performance of the random forest model. (B) ROC analysis showing the performance of the gradient boosting decision tree. **Fig. S10.** ROC curve analysis was conducted to evaluate the performance of models for cluster analysis within lineage 2.2.2. (A) ROC analysis showing the performance of the random forest model. (B) ROC analysis showing the performance of the gradient boosting decision tree. **Fig. S11.** ROC curve analysis was conducted to evaluate the performance of models for cluster analysis within lineage 4.1. (A) ROC analysis showing the performance of the random forest model. (B) ROC analysis showing the performance of the gradient boosting decision tree. **Fig. S12.** ROC curve analysis was conducted to evaluate the performance of models for cluster analysis within lineage 4.2. (A) ROC analysis showing the performance of the random forest model. (B) ROC analysis showing the performance of the gradient boosting decision tree. **Fig. S13.** ROC curve analysis was conducted to evaluate the performance of models for cluster analysis within lineage 4.3. (A) ROC analysis showing the performance of the random forest model. (B) ROC analysis showing the performance of the gradient boosting decision tree. **Fig. S14.** ROC curve analysis was conducted to evaluate the performance of models for cluster analysis within lineage 4.4. (A) ROC analysis showing the performance of the random forest model. (B) ROC analysis showing the performance of the gradient boosting decision tree. **Fig. S15.** ROC curve analysis was conducted to evaluate the performance of models for cluster analysis within lineage 4.8. (A) ROC analysis showing the performance of the random forest model. (B) ROC analysis showing the performance of the gradient boosting decision tree. **Fig. S16.** The ROC curve analysis was conducted to evaluate the performance of models for cross-country analysis within lineage 2. (A) ROC analysis showing the performance of the random forest model. (B) ROC analysis showing the performance of the gradient boosting decision tree. **Fig. S17.** The ROC curve analysis was conducted to evaluate the performance of models for cross-country analysis within lineage 4. (A) ROC analysis showing the performance of the random forest model. (B) ROC analysis showing the performance of the gradient boosting decision tree. **Fig. S18.** The ROC curve analysis was conducted to evaluate the performance of models for cross-regional analysis within lineage 2. (A) ROC analysis showing the performance of the random forest model. (B) ROC analysis showing the performance of the gradient boosting decision tree. **Fig. S19.** The ROC curve analysis was conducted to evaluate the performance of models for cross-regional analysis within lineage 4. (A) ROC analysis showing the performance of the random forest model. (B) ROC analysis showing the performance of the gradient boosting decision tree.

**Additional file 2:** **Table S1.** Information of 1445 strains of mycobacterium tuberculosis. **Table S2.** Information of 12086 strains of mycobacterium tuberculosis. **Table S3.** Information of gene mutations in two-component systems. **Table S4.** Important scores of each feature of the random forest model in cluster. **Table S5.** Important scores of each feature of the gradient boosting decision tree in cluster. **Table S6.** Important scores of each feature of various models in cluster size.**Table S7.** Important scores of each feature of various models in cross country. **Table S8.** Important scores of each feature of various models in cross regional. **Table S9.** The performance of various models for discriminating clustered strains from non-clustered strains in the lineage1 cohort. **Table S10.** The performance of various models for discriminating clustered strains from non-clustered strains in the lineage3 cohort. **Table S11.** The performance of various models for discriminating clustered strains from non-clustered strains in the lineage4 cohort. **Table S12.** The performance of various models for discriminating clustered strains from non-clustered strains in the lineage2.2.1 cohort. **Table S13.** The performance of various models for discriminating clustered strains from non-clustered strains in the lineage2.2.2 cohort. **Table S14.** The performance of various models for discriminating clustered strains from non-clustered strains in the lineage4.1 cohort. **Table S15.** The performance of various models for discriminating clustered strains from non-clustered strains in the lineage4.2 cohort. **Table S16.** The performance of various models for discriminating clustered strains from non-clustered strains in the lineage4.3 cohort. **Table S17.** The performance of various models for discriminating clustered strains from non-clustered strains in the lineage4.4 cohort. **Table S18.** The performance of various models for discriminating clustered strains from non-clustered strains in the lineage4.8 cohort. **Table S19.** The performance of various models for discriminating cluster size in the lineage2 cohort. **Table S20**. The performance of various models for discriminating cluster size in the lineage4 cohort. **Table S21.** The performance of various models for discriminating cross-country from within-country in the lineage2 cohort. **Table S22.** The performance of various models for discriminating cross-country from within-country in the lineage4 cohort. **Table S23.** The performance of various models for discriminating cross-regional from within-regional in the lineage2 cohort. **Table S24.** Generalized linear mixed model analysis on clustered and non-clustered strains in the lineage1 cohort. **Table S25.** Generalized linear mixed model analysis on clustered and non-clustered strains in the lineage2 cohort. **Table S26.** Generalized linear mixed model analysis on clustered and non-clustered strains in the lineage3 cohort. **Table S27.** Generalized linear mixed model analysis on clustered and non-clustered strains in the lineage4 cohort. **Table S28.** Generalized linear mixed model analysis on clustered and non-clustered strains in the lineage2.2.1 cohort. **Table S29.** Generalized linear mixed model analysis on clustered and non-clustered strains in the lineage4.1 cohort. **Table S30.** Generalized linear mixed model analysis on clustered and non-clustered strains in the lineage4.2 cohort. **Table S31.** Generalized linear mixed model analysis on clustered and non-clustered strains in the lineage4.4 cohort. **Table S32.** Generalized linear mixed model analysis on clustered and non-clustered strains in the lineage4.8 cohort. **Table S33.** Generalized linear mixed model analysis on cluster size in the lineage4.8 cohort. **Table S34.** Generalized linear mixed model analysis on cluster size in the lineage4 cohort. **Table S35.** Generalized linear mixed model analysis on cross-country strains in the lineage2 cohort. **Table S36.** Generalized linear mixed model analysis on cross-country strains in the lineage4 cohort. **Table S37.** Generalized linear mixed model analysis on cross-regional strains in the lineage2 cohort. **Table S38.** Generalized linear mixed model analysis on cross-regional strains in the lineage4 cohort.
